# Safety and Efficacy of Ephedrine Alkaloids-Free Ephedra Herb Extract (EFE) for Mild COVID-19: A Double-Blind, Placebo-Controlled, Randomized Comparative Trial

**DOI:** 10.3390/microorganisms13030641

**Published:** 2025-03-12

**Authors:** Hiroshi Odaguchi, Sumiko Hyuga, Mariko Sekine, Hirofumi Michimae, Masashi Hyuga, Nahoko Uchiyama, Masashi Uema, Yuji Kumagai, Yusuke Suzuki, Shigeki Nabeshima, Norio Omagari, Yohei Doi, Kunihiro Yamaoka, Koji Miyazaki, Susumu Fuji, Yoshihiro Umezawa, Shiho Kodera, Hirotaka Nagashima, Wataru Hirose, Yukihiro Goda

**Affiliations:** 1 Oriental Medicine Research Center, School of Pharmacy, Kitasato University, Tokyo108-8641, Japan; odaguchi@insti.kitasato-u.ac.jp; 2 Kitasato University Kitasato Institute Hospital, Shirokane Campus, Tokyo 108-8642, Japan; m.sekine@insti.kitasato-u.ac.jp (M.S.); kuma-guy@za2.so-net.ne.jp (Y.K.); yusuke-s@insti.kitasato-u.ac.jp (Y.S.); 3 Department of Clinical Medicine (Biostatistics), School of Pharmacy, Kitasato University, Tokyo 108-8641, Japan; michimaeh@pharm.kitasato-u.ac.jp; 4 National Institute of Health Sciences, Kawasaki 210-9501, Japan; mhyuga@nihs.go.jp (M.H.); nuchiyama@nihs.go.jp (N.U.); m-uema@nihs.go.jp (M.U.); goda@nihs.go.jp (Y.G.); 5 Department of General Medicine, Faculty of Medicine, Fukuoka University, Fukuoka 814-0180, Japan; snabeshi@fukuoka-u.ac.jp; 6 Disease Control and Prevention Center, National Center for Global Health and Medicine, Tokyo 162-8655, Japan; nohmagari@hosp.ncgm.go.jp; 7 School of Medicine, Fujita Health University, Toyoake 470-1192, Japan; yoheidoi@fujita-hu.ac.jp; 8 Department of Rheumatology and Infectious Diseases, Kitasato University Hospital, Sagamihara 252-0375, Japan; yamaoka@med.kitasato-u.ac.jp; 9 Department of General Internal Medicine, Tokai University Hachioji Hospital, Tokyo 192-0032, Japan; kmiyazaki@tsc.u-tokai.ac.jp; 10 Ogikubo Hospital, Tokyo 167-0035, Japan; susumuf3@yahoo.co.jp; 11 Denenchofu Family Clinic, Tokyo 145-0071, Japan; plum@abelia.ocn.ne.jp; 12 Tokyo Metropolitan Ebara Hospital, Tokyo 145-0065, Japan; shihokode@yahoo.co.jp; 13 Tokyo Center Clinic, Tokyo 192-0397, Japan; nagashima_hirotaka@tc-clinic.jp; 14 Hirose Clinic, Tokorozawa 359-1111, Japan; hirose.rheum@gmail.com

**Keywords:** SARS-CoV-2, COVID-19, EFE, DB-RCT, Kampo medicine, crude drug, Ephedra Herb, maoto, side effects

## Abstract

Several Ephedra Herb-containing Kampo medicines are common initial treatments for various infections; however, the ephedrine alkaloids in Ephedra Herb can cause side effects by stimulating adrenergic receptors. Accordingly, an ephedrine alkaloids-free Ephedra Herb Extract (EFE) has been developed. This study aimed to evaluate whether EFE can be used effectively and safely in patients with mild coronavirus disease 2019 (COVID-19). We randomized patients with mild COVID-19 to receive EFE equivalent to 6 g of Ephedra Herb per day or a placebo for 14 days. The primary efficacy endpoint was the non-aggravation rate up to Day 15. We allocated 41 and 40 patients to the EFE and placebo groups, respectively. All participants were included in the mITT and safety analysis populations [male ratio, mean age: 31.7%, 42.0 years (EFE); 17.5%, 43.2 years (placebo)]. The non-aggravation rate up to Day 15 for the primary endpoint was 100.0% and 94.6% in the EFE and placebo group, respectively, with no between-group difference. The number of days to the improvement in nausea symptoms was significantly shorter in the EFE group. One patient in the placebo group discontinued the trial due to a side effect. Although EFE demonstrated safety in patients with mild COVID-19, it did not show superior efficacy compared to placebo for symptoms other than nausea.

## 1. Introduction

The global pandemic due to Coronavirus disease 2019 (COVID-19), a viral infection caused by severe acute respiratory syndrome coronavirus 2 (SARS-CoV-2), began in 2020 [1]. Many patients with COVID-19 are asymptomatic or only experience mild upper respiratory inflammation. However, some patients, particularly the elderly and those with underlying pre-existing conditions, are at a high risk of developing severe diseases, including pneumonia, respiratory failure, and death [1,2]. Although vaccines and oral antiviral drugs are now available [3,4], high-risk patients may still develop severe disease even after vaccination [5]. Further, the antiviral drugs are expensive, with studies raising concerns about their effectiveness against certain variants [6,7]. Since COVID-19 has become a common viral infection, there is a need for affordable, accessible, and safe treatment options for high-risk patients in the early stages of infection.

Kampo medicine refers to traditional Japanese medicine and is widely used in daily clinical practice in Japan [8,9,10]. Kampo medicines generally consist of multiple crude drugs and are used based on the theories of Kampo medicine. For the initial treatment of various infections, several Kampo medicines containing Ephedra Herb are often used based on these theories. The representative Kampo medicine containing Ephedra is maoto. Maoto has been reported to have effects equivalent to neuraminidase inhibitors for seasonal influenza [11]; accordingly, it has been useful for children with seasonal influenza who have low sensitivity to neuraminidase inhibitors [12]. Additionally, Ephedra Herb, one of the crude drugs in maoto, has demonstrated anti-influenza virus activity [13]. However, the ephedrine alkaloids in Ephedra Herb can cause side effects such as insomnia, agitation, palpitations, arrhythmia, hypertension, and dysuria due to the stimulation of adrenergic receptors [14]. Accordingly, to eliminate the side effects of Ephedra Herb, an ephedrine alkaloids-free Ephedra Herb Extract (EFE) was developed [15]. Various pharmacological effects of EFE have been reported, including analgesic, anticancer, and anti-influenza activities [16,17]. Moreover, EFE has not been associated with the adverse events typically induced by Ephedra Herb Extract [18] and has been shown to be safe for healthy adults [19]. Additionally, recent in vitro studies have demonstrated that EFE has antiviral effects against SARS-CoV-2 [20].

This clinical trial aimed to verify whether EFE can be used effectively and safely in patients with mild COVID-19.

## 2. Materials and Methods

### 2.1. Study Design

This clinical trial consisted of two parts, Part 1 and Part 2. The primary focus was on Part 2; however, Part 1 was established as a phase to carefully observe and verify the safety and efficacy of EFE in patients with COVID-19 under hospitalization to ensure the safety of EFE in patients with COVID-19 before moving to Part 2.

Part 1 was conducted from June to December 2021 as a single-arm, multicenter collaborative trial under hospitalization, where EFE preparation was administered to five patients. In December 2021, the Data and Safety Monitoring Board determined that there were no safety issues with EFE preparation in these patients with COVID-19, approving the transition to Part 2.

Part 2 was conducted from February 2022 to March 2023 as a double-blind, randomized, placebo-controlled, multicenter collaborative comparative trial under either non-hospitalized or hospitalized conditions. A total of 81 patients were enrolled across six trial sites.

This clinical trial design was determined under the guidance of Pharmaceuticals and Medical Devices Agency (PMDA) in Japan, and the trial was registered with the Japan Registry of Clinical Trials (jRCT: https://jrct.niph.go.jp/, accessed on 30 April 2021) under the identifier jRCT2031210063.

### 2.2. Patients

We included patients aged 20 to <80 years at the time of enrollment who tested positive for SARS-CoV-2 by PCR or antigen test within 3 days prior to enrollment and had COVID-19 symptoms (including fever or upper respiratory inflammation symptoms like cough) within 7 days that were classified as mild (upper respiratory symptoms such as cough but no respiratory symptoms like dyspnea; resting arterial oxygen saturation [SpO_2_] ≥ 96%). The study doctor had to determine the appropriateness of participation based on medical examination, vital signs, clinical laboratory results, and chest radiographic findings. Written informed consent was obtained from all participants. Patients were excluded if they had household members participating in this trial or who had previously participated in this trial. This was because EFE is derived from crude drugs, and its distinctive smell and taste could not be easily replicated by the placebo. Therefore, trial-experienced household members could potentially allow the identification of the active drug versus the placebo based on smell or color. The exclusion criteria for this trial are listed in Supplemental Information S1.

### 2.3. Study Drugs

The raw material, Ephedra Herb, was defined in the eighteenth edition of the Japanese Pharmacopoeia (JP). EFE preparations were manufactured by removing alkaloids from a decoction of Ephedra Herb using cation exchange column chromatography, followed by freeze-drying and the addition of excipients. The EFE preparation was prepared from 6 g of Ephedra Herb, with added excipients (dextrin) to make 1.5 g, which was designated as the daily dose.

The placebo was made by adding colorants to the excipients to match the color of the EFE preparation. The smell and taste were not adjusted. The daily placebo dose was also set at 1.5 g. Both EFE preparation and placebo were provided in packets of 750 mg each, with a daily dose consisting of two packets taken twice a day.

### 2.4. Placebo

Since this clinical trial is a double-blind test, it is necessary to color the placebo to make it difficult to distinguish it from the active drug. In this case, we decided to use “Aluminum Lake of Tartrazine” and “Aluminum Lake of Brilliant Blue FCF”, which are tar dyes that can be used in medicines and other products as specified by the Ministry of Health, Labor and Welfare (MHLW), as well as “Caramel Color” and “Iron Oxide Red”, which were specified in advance in the investigator’s brochure, and identification tests were conducted. Notably, these additives are also used in food, and we use them within the permitted usage range.

### 2.5. Treatment

#### 2.5.1. Part 1

All participants were given EFE preparations for 14 days. One packet each of EFE preparation was given twice per day, after awaking up and before sleeping.

#### 2.5.2. Part 2

Participants were randomly assigned in a 1:1 ratio to the EFE preparation or placebo group. The trial was blinded for patients, study doctors, trial collaborators, study drug managers, and all other personnel involved in the study data, including monitoring personnel, by not disclosing the type of study drug administered.

Participants received EFE preparations or placebo for 14 days according to their assigned group. EFE preparation or placebo was administered twice daily, one packet each in the morning upon waking and one packet before sleeping.

During the trial period, participants were allowed to continue taking their regular medications and supplements for pre-existing conditions (e.g., antihypertensive drugs). Moreover, they were allowed to take antipyretic analgesics, cough suppressants, and expectorants for symptom relief. However, the use of COVID-19 treatments, unapproved drugs expected to be effective against COVID-19, and corticosteroids (oral or inhaled) was prohibited.

### 2.6. Outcomes

The primary efficacy endpoint was the non-aggravation rate up to Day 15. In this trial, severe progression was defined as a resting SpO_2_ <96% and a ≥2% decrease from the pre-screening average SpO_2_. The non-aggravation rate was defined as the proportion of patients without severe progression.

The secondary efficacy endpoints included the SARS-CoV-2 PCR negative conversion rate on Day 15; the frequency of use of antipyretic analgesics, cough suppressants, and expectorants; the number of days until the body temperature was below 37.0 °C twice consecutively without the use of antipyretic analgesics; the degree of improvement in symptoms and pain symptoms, as determined by the Face Scale (FS) for malaise, cough, nasal discharge, nausea, and pain such as joint pain and headache (Supplemental Figure S1); the number of days until the FS score for symptoms and pain symptoms improved to 0 or 1; the non-aggravation rate up to Day 21; the proportion of patients not progressing to severe disease (requiring management with a ventilator or ECMO) by Day 15 and Day 21; and the disease status score on a six-point ordinal scale on Day 7, Day 15 or at the time of discontinuation, and Day 21 or 1 week after discontinuation (Supplemental Information S2).

Safety assessment included monitoring adverse events, the incidence of side effects, and clinical laboratory results along with vital signs. The severity of adverse events was evaluated using the Common Terminology Criteria for Adverse Events version 5.0 (https://ctep.cancer.gov/protocolDevelopment/electronic_applications/ctc.htm#ctc_50, accessed on 23 October 2020). Side effects were defined as any adverse event with a potential causal relationship with the study drug.

### 2.7. Statistical Analysis

#### 2.7.1. Part 1

The target number of patients was set at six to determine whether a safe trial with EFE preparation in an outpatient setting was feasible and sufficient. The analysis population comprised patients who received the study drug at least once and were observed during the study.

For safety analysis, adverse events and side effects were assessed by calculating the number of occurrences, incidence rates, and their 95% confidence intervals (CIs). CIs were computed using the Clopper–Pearson method. Similar analyses were conducted with stratification according to severity and seriousness of adverse events. Summary statistics for clinical laboratory results and vital signs were calculated according to measurement periods. Additionally, the summary statistics of changes from baseline were computed for different time points after the initiation of treatment.

The analysis for efficacy and safety followed the methodology used in Part 2 of the trial. Since Part 1 was conducted as a single-group study, comparisons between treatment groups were not performed.

#### 2.7.2. Part 2

The rationale for setting the target number of cases was as follows: Based on the *Clinical Management of Patients with COVID-19: A guide for front-line healthcare workers Version 2.1*, which was issued by the Japanese government research team in 2020 (https://www.niph.go.jp/h-crisis/wp-content/uploads/2020/07/20200706103735_content_000646531.pdf, accessed on 12 July 2020), it was assumed that 15% of patients with mild COVID-19 would have severe progression, which served as the predicted aggravation rate for the placebo group. For the EFE preparation treatment group, a predicted aggravation rate of 2% was assumed. To detect a 13% reduction in aggravation due to EFE preparation treatment with 80% power and a significance level of 5% (two-sided chi-square test), it was calculated that each group would require 144 patients. By anticipating a dropout rate of ≈5% in each group, a total of 150 patients (75 per group) were considered necessary for enrollment.

We defined two analysis populations for assessing efficacy: the modified Intent-To-Treat (mITT) population and the Per-Protocol Set (PPS) population according to the protocol. The mITT population included all enrolled patients who received the study drug at least once, and it served as the primary population for efficacy analysis in this trial. The PPS population comprised the mITT population excluding patients who may affect efficacy evaluation due to non-compliance with inclusion criteria. Exclusion criteria for the PPS population corresponds to the violation of exclusion criteria, which corresponds to the violation of prohibited concomitant drug use, and so on (Supplemental Information S3).

For safety analysis, we considered the population of patients who received the study drug at least once and were observed during the study.

Regarding the primary efficacy endpoint analysis, we evaluated the number and proportion of patients who did not progress to severe illness by Day 15 (non-aggravation rate) and their 95% CIs within each treatment group using the mITT population. We performed between-group comparisons of the non-aggravation rates using the chi-square test. The same analyses were conducted for the PPS population.

In the analysis of secondary endpoints regarding effectiveness, the analysis was conducted targeting the mITT population. Regarding the SARS-CoV-2 negativity rate at Day 15, the number of patients confirmed to have tested negative for SARS-CoV-2 by Day 15, their proportions, and the 95% CIs were calculated for each treatment group, with between-group comparisons performed using a chi-squared test. The method used to confirm negativity is described in Table S1.

Regarding the frequency of use of symptomatic relief medication, the number of patients who used symptomatic relief medication during the trial, their proportions, and the 95% CIs were calculated for each treatment group, with between-group comparisons using a chi-squared test. Furthermore, the summary statistics of the total number of uses of symptomatic relief medication during the trial period were calculated for each treatment group, with between-group comparisons analyzed using the Wilcoxon rank-sum test.

Regarding the number of days until the body temperature was below 37.0 °C for two consecutive times (defined as an “event”), between-group comparisons of the time until this event were performed using the Log-rank test. Additionally, using the Kaplan–Meier method, the cumulative incidence rates of events at each time point along with their 95% CIs were calculated for each group. Kaplan–Meier plots of the cumulative incidence rates were also generated for each group.

Regarding the improvement in FS score related to symptoms/pain symptoms, the summary statistics of FS observed daily from Day 1 to Day 15 were calculated for each group. At each time point, the summary statistics of the change in the FS score from Day 1 were also calculated for each group. Between-group comparisons were conducted using the Wilcoxon rank-sum test. For the number of days until the FS score for symptoms/pain symptoms improved to 0 or 1, each symptom/pain symptom was considered an “event” when the FS score reached 0 or 1. The time until this event was confirmed, with between-group comparisons using the Log-rank test. Furthermore, using the Kaplan–Meier method, the cumulative incidence rates of events at each time point and their 95% CIs were calculated for each group. Kaplan–Meier plots of the cumulative incidence rates were also generated.

For the non-aggravation rate by Day 21, the number of patients who did not show severe progression by Day 21, their proportions (non-aggravation rate), and 95% CIs were calculated for each treatment group. These non-aggravation rates were compared between the treatment groups using a chi-squared test.

Regarding the proportion of patients who did not develop severe symptoms by Day 15 and Day 21, the number of patients who remained non-severe by each observation day, their proportions, and 95% CIs were calculated for each treatment group. Between-group comparisons of these proportions were performed using a chi-squared test.

For the disease status score based on the six-point ordinal scale at Day 7, Day 15/at discontinuation, and Day 21/1 week post-discontinuation, the number of patients and their proportions for each severity score at each time point were calculated for each treatment group. Group comparisons were conducted using the Wilcoxon rank-sum test. Additionally, analyses were performed when combining data from upon discontinuation and Day 15, as well as when combining data from 1-week post-discontinuation and Day 21.

During efficacy analysis, if concomitant prohibited medications were used during the trial period, efficacy assessment data measured after the start date of concomitant use were not included in the analysis.

For adverse events and side effects, the number of occurrences, the number of cases, the incidence rate, and their 95% CIs were calculated. The Clopper–Pearson method was used to calculate the CIs. Similar analyses were conducted based on the severity and seriousness of the events. For clinical laboratory results and vital signs, the summary statistics of the measurements were calculated for each time point. Additionally, the summary statistics of the changes from baseline were calculated for each time point after the start of administration.

When testing, the significance level is set at 5% two-sided.

### 2.8. Ethical Considerations

This trial was conducted in accordance with the “Declaration of Helsinki (Ethical Principles for Medical Research Involving Human Participants)”, the standards specified in Articles 14-3 and 80-2 of the “Pharmaceuticals and Medical Devices Act”, and the “Ministerial Ordinance on Good Clinical Practice (GCP).” Furthermore, the trial was conducted based on the content approved by the Institutional Review Board (IRB in Center Hospital of the National Center for Global Health and Medicine; approval code: I-016-20a; approval date: 3 March 2021). Written informed consent was obtained from all patients.

## 3. Results

### 3.1. Part 1

Between June and December 2021, five individuals were enrolled in the study. EFE preparation was administered to all participants. In all cases, drug administration was completed without any deviations from the protocol, resulting in a 100% medication adherence rate.

Among the included patients, there was one male (20.0%) and four females (80.0%). The age [mean ± standard deviation, minimum to maximum (same hereinafter)] was 35.0 ± 15.7 years, with the male being 28.0 years old and the age of females ranging from 27 to 63 years. The mean BMI was 21.96 ± 3.28 kg/m^2^, with the male having a BMI of 20.70 kg/m^2^ and the females’ BMI ranging from 19.1 to 27.5 kg/m^2^. The mean body temperature was 37.06 ± 0.56 °C, with the male’s temperature being 37.20 °C and the females’ temperatures ranging from 36.3 to 37.7 °C. The mean SpO_2_ was 98.30 ± 1.15%, with the male’s SpO_2_ at 98.50% and the females’ SpO_2_ ranging from 96.5 to 99.5% (Table S2).

All five patients were included in the Part 1 analysis population. Regarding the primary endpoint related to the efficacy of EFE, none of the five patients progressed to severe disease by Day 15.

Regarding safety, adverse events were reported in two out of five cases (40.0%), with a total of six incidents. Side effects were observed in one (20.0%) patient, with three incidents reported. No adverse events led to trial discontinuation. Regarding the severity of adverse events, Grade 1 events occurred six times in two out of five patients. No adverse events of Grade 2 or higher were observed (Table 1).

The Data and Safety Monitoring Committee reviewed these results in December 2021. The committee determined that administering EFE preparation to patients with COVID-19 outside of a hospital setting posed no safety concerns; accordingly, they granted approval to proceed to Part 2 of the trial.

### 3.2. Part 2

#### 3.2.1. Participant Composition

**Figure 1:**
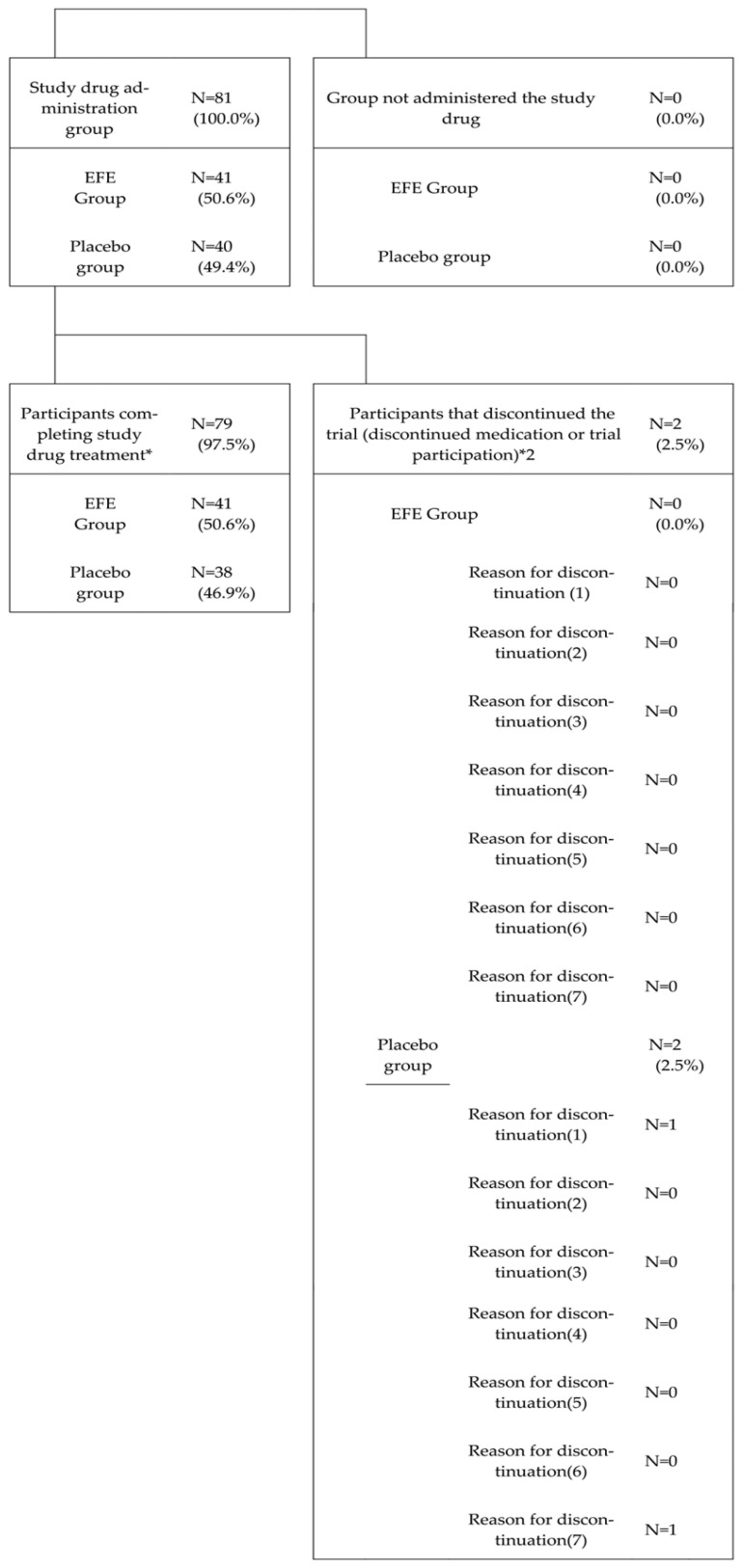
Part 2: Participant composition (participants giving consent to Part 2). *: Participants completing Day1–Day14 treatments *2: Reason for termination: (1) If the patient requests to withdraw from the study or revokes their consent. (2) If it is discovered after enrollment that the patient does not meet the inclusion criteria or violates the exclusion criteria. (3) If prohibited concomitant medications are used. (4) If it becomes difficult to continue the study due to adverse events. (5) If the patient’s symptoms worsen to moderate or severe during the study period. (6) If the entire study is discontinued. (7) If the study doctor deems it appropriate to discontinue the study for any other reason.

Between February 2022 and March 2023, we enrolled 81 individuals to Part 2 of the trial. The trial ended before reaching the target of 150 participants. The included patients were randomly assigned to either the EFE group (41 [50.6%]) or the placebo group (40 [49.4%]) (same order hereinafter). In both treatment groups, all participants received the study drug. The dropout rates were 0 out of 41 cases (0.0%) for the EFE group and 2 out of 81 cases (5%) for the placebo group. The completion rates of the study drug administration were 41 out of 41 cases (100%) for the EFE group and 38 out of 40 cases (95.0%) for the placebo group (Figure 1). The reasons for discontinuation in the two cases from the placebo group were as follows: one patient withdrew consent, and the study doctor determined that it could not be ruled out that the adverse events in the other case were related to the study drug.

#### 3.2.2. Patient Background and Physical Findings

There were no between-group differences in patient backgrounds and physical findings. In terms of sex, the EFE group included 13 males (31.7%) and 28 females (68.3%), while the placebo group included 7 males (17.5%) and 33 females (82.5%). There was a higher proportion of females than males in both groups. The age [mean ± standard deviation (median, minimum to maximum) (same hereinafter)] was 42.0 ± 11.8 years (42.0 years, range 21–69 years) in the EFE group and 43.2 ± 11.2 years (41.5 years, range 22–73 years) (same order hereinafter) in the placebo group. The BMI was 22.33 ± 3.53 kg/m^2^ (21.40 kg/m^2^, range 17.0–32.4 kg/m^2^) in the EFE group and 22.20 ± 2.98 kg/m^2^ (21.55 kg/m^2^, range 16.8–28.6 kg/m^2^) in the placebo group. The body temperature was 36.66 ± 0.35 °C (36.70 °C, range 35.6–37.6 °C) in the EFE group and 36.78 ± 0.77 °C (36.65 °C, range 35.6–39.9 °C) in the placebo group. The SpO_2_ was 98.23 ± 0.63% (median 98.50%, range 96.5–99.5%) in the EFE group and 98.20 ± 0.67% (median 98.00%, range 97.0–99.5%) in the placebo group (Table S3).

#### 3.2.3. Analysis Population

All patients in both groups were included in the mITT and safety analysis populations. The PPS included 33 out of 41 patients (80.5%) in the EFE group and 32 out of 40 patients (80.0%) in the placebo group (same hereinafter). Details regarding the acceptance and rejection of patients in the analysis populations are listed in Table S4.

#### 3.2.4. Efficacy

Efficacy analysis was conducted using the mITT population. The non-aggravation rate by Day 15 was 100.0% (95% CI: 90.7–100.0%) in the EFE group (38/38 cases) and 94.6% (95% CI: 81.8–99.3%) in the placebo group (35/37 cases), with no significant between-group difference. The results for the PPS population were similar (refer to Table 2).

**Figure 2:**
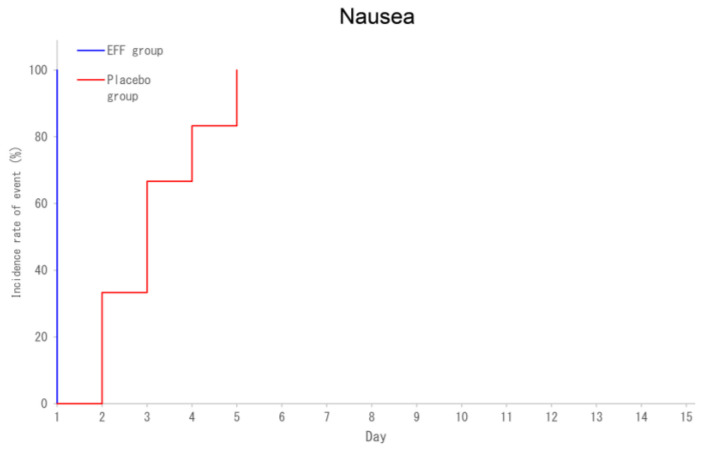
Part 2: secondary endpoint (5): the number of days until the FS score for nausea symptoms becomes 0 or 1 (mITT).

There were no significant between-group differences in the following secondary endpoints: the SARS-CoV-2 negative conversion rate on Day 15, frequency of use of symptomatic relief medication, number of days until the body temperature dropped below 37.0 °C, non-aggravation rate by Day 21, proportion of patients who did not develop severe disease by Days 15 and 21, and disease status scores based on the six-point ordinal scale at each time point. Regarding the improvement in the FS score of symptom/pain symptoms, the number of days until the FS score for nausea symptoms reached 0 or 1 was significantly shorter in the EFE group than in the placebo group (the details of the secondary endpoints are listed in Table S5–S10 and Figure 2).

#### 3.2.5. Safety

Safety analysis was conducted using the safety analysis population. Adverse events occurred in 2 out of 41 cases (4.9%), with 2 incidents in the EFE group, and in 6 out of 40 cases (15.0%), with 6 incidents in the placebo group. In the EFE group, one patient presented a Grade 1 event (abnormal liver function) and another one presented a Grade 2 event (eczema). In the placebo group, there were four Grade 1 events (cystitis, upper respiratory tract infection, pharyngotonsillitis, and abnormal liver function, each occurring once) in 4 out of 40 cases, as well as two Grade 2 events (sinusitis and urticaria, each occurring once) in 2 out of 40 cases. None of the patients showed severe adverse events of Grade 3 or higher. Adverse events leading to trial discontinuation only occurred in one patient (2.5%) in the placebo group. Side effects were reported in two (4.9%) patients (two incidents) in the EFE group and in one (2.5%) patient (one incident) in the placebo group. In the EFE group, there was one Grade 1 side effect (abnormal liver function test) in one patient and one Grade 2 side effect (eczema) in another patient. No side effects of Grade 3 or higher were reported. In the placebo group, there was one Grade 1 side effect (abnormal liver function) in one patient, with no side effects of Grade 2 or higher being observed (Table S11). Side effects leading to trial discontinuation only occurred in one patient (2.5%) in the placebo group. This patient developed Grade 1 abnormal liver function three days after starting the study drug, leading to the discontinuation of the study drug. The concomitant medications were also discontinued, and the adverse event resolved 50 days after its onset.

None of the included patients presented notable changes in physical findings (systolic blood pressure, diastolic blood pressure, body temperature, pulse rate, SpO_2_) or clinical laboratory results (Supplemental Table S12).

## 4. Discussion

Kampo medicines containing Ephedra Herb have been reported to be effective against COVID-19 [21,22]. The antiviral activity of these medicines is thought to be mainly due to Ephedra Herb, and the herb has been reported to be a candidate with high potential for the control and prevention of COVID-19 [23]. However, Ephedra Herb has side effects such as excitation, insomnia, and arrhythmia derived from ephedrine alkaloids. Therefore, Ephedra Herb is contraindicated for the elderly and those with weakened physical strength. On the other hand, EFE has been reported to have anti-SARS-CoV-2 activity [20] and is safer than Ephedra Herb. Therefore, we have conducted the clinical trial using EFE. The dose of the study drug EFE was the dose used in the study for evaluating the clinical safety of EFE [19], and was calculated based on the maximum dosage (6 g/day) of Ephedra Herb administered as medicine in Japan. It is possible that increasing the dose of EFE could increase its effectiveness. However, the Pharmaceuticals and Medical Devices Agency (PMDA) in Japan did not approve the conduct of clinical trials with an increased dose of EFE due to safety concerns, although we thought that EFE was safer than Ephedra Herb itself.

In Part 1, EFE preparation was administered to five patients, which fell short of the target of six patients. This was because shortly after the trial began, the so-called fifth wave of the COVID-19 pandemic started in Japan, which led to a drastic increase in the number of patients with COVID-19 and a lack of available hospital beds. This impeded medical institutions from conducting clinical trials under inpatient conditions. Subsequently, the fifth wave subsided rapidly, with a significant decrease in the number of patients with COVID-19. Therefore, since there were difficulties in enrolling participants, it was determined that the number of cases initially set to sufficiently assess the safety for proceeding to Part 2 did not need to be strictly six. Accordingly, it was considered that the feasibility assessment could be adequately performed with five cases. Since EFE preparation was administered to all five cases, evaluating efficacy was challenging; however, none of the patients progressed to severe disease. Regarding safety, only three minor side effects (elevation of C-reactive protein, increased neutrophil count, increased white blood cell count) were reported. Based on these findings, the safety evaluation committee determined that there were no issues in proceeding to Part 2.

In Part 2, the clinical utility of EFE preparation was evaluated in a double-blind, randomized, placebo-controlled, multicenter comparative trial. There were no significant between-group differences in patient backgrounds.

The primary endpoint, the non-aggravation rate on Day 15, showed no between-group difference in the mITT population, with similar results being observed in the PPS population. Regarding the secondary endpoints, although the EFE group was superior in terms of time to nausea improvement with the FS score of 0 or 1, there were no significant between-group differences in the other measures. Considering reports [20] that EFE and its active component, high-molecular condensed tannin (Ephedra Herb macromolecule condensed-tannin: EMCT) [24], inhibit the proliferation of SARS-CoV-2 and its several variants, EFE was anticipated to exhibit clinical efficacy against COVID-19. However, this trial did not demonstrate the superiority of EFE.

This study has several limitations. First, although the target number of cases for Part 2 was 150, only 81 participants were enrolled. This could be attributed to several factors, including the strain on healthcare facilities due to the COVID-19 pandemic, competition for potential participants with concurrent clinical trials for new antiviral drugs, and funding shortage. This reduced number of cases may have led to insufficient statistical power, which may have affected the findings. Second, at the time this trial was planned, the typical clinical course of COVID-19, as described in the “*Clinical Management of Patients with COVID-19: A Guide for Front-line Healthcare Workers Version 2.1*”, was as follows: among patients who developed symptoms of upper respiratory tract infection, 80% recover with mild symptoms while 20% experience the worsening of pneumonia that requires hospitalization. Additionally, 2–3% of cases may further deteriorate to a fatal condition. Accordingly, this trial set the primary endpoint as the non-aggravation rate and calculated the required sample size accordingly. However, this clinical course was based on SARS-CoV-2 infection prior to the emergence of the Omicron variant and did not account for immunity from vaccines, which was still in development at that time. During the trial course, major circulating variants of SARS-CoV-2 evolved globally, and by the time Part 2 was conducted, the dominant variant in Japan was Omicron. Moreover, vaccines had been developed by the time the trial was underway, and a significant portion of the population had been vaccinated. COVID-19 caused by the Omicron variant has been shown to be less likely to progress to severe disease [25,26], and vaccination has reduced the overall severity of COVID-19 [27,28]. In our study, there were no cases of severe disease in either the placebo or EFE groups, which further suggests that the setting of the evaluation endpoints and the calculation of the required sample size was inadequate. This limitation might have contributed to the lack of observed between-group differences.

Regarding the improvement in the FS score of symptoms, the time required for nausea symptoms to reach an FS score of 0 or 1 was significantly shorter in the EFE group than in the placebo group (Figure 2). Nausea and vomiting have been reported to occur in 10.2% of patients with COVID-19 [29]; further, in the present study, nausea symptoms were observed in five (12%) and six (15%) cases in the EFE and placebo groups, respectively. This is the first study to demonstrate that EFE can improve nausea. Nishi et al. analyzed the effects of oral EFE in a mouse model infected with mouse coronavirus, the murine hepatitis virus (MHV-1, ATCC VR-261), which belongs to the order Nidovirus in the family Coronaviridae, and multiplies in the liver and lung, and exhibits infectious symptoms similar to SARS-CoV-2 such as hepatitis and pneumonia [30,31], and found that a single dose of 700 mg/kg of EFE tended to reduce viral loads in the lungs more effectively than administering 350 mg/kg twice daily. Accordingly, to achieve the antiviral effects of EFE, it may be necessary to increase the dose per administration to temporarily elevate the concentration of the active ingredient in the bloodstream. In this study, EFE equivalent to 6 g of ephedra was divided into two daily doses. However, administering it as a single dose might have enhanced the antiviral effect and yielded more significant results. The study followed the dosage regimen that had been confirmed for safety in healthy adults [19]; however, further studies are warranted to explore dosage regimens that could potentially enhance antiviral effects.

Regarding safety, none of the patients presented serious adverse events, and only one case of an adverse event leading to trial discontinuation was observed in the placebo group. This further demonstrates the high safety profile of EFE. Specifically, although the safety of EFE in healthy adults has already been confirmed [19], our findings further demonstrate that EFE can be used safely in patients with mild COVID-19.

## 5. Conclusions

Although EFE demonstrated safety in patients with mild COVID-19, it did not show superior efficacy compared to placebo for symptoms other than nausea.

## 6. Patents

Patent number: 7214080 (Japan); title of the invention: antiviral agent for preventing or treating new coronavirus infection; applicants: Kitasato Institute, Director of the National Institute of Health Sciences, Matsuyama University, Tokiwa Phytochemical Research Institute, and Tsumura Co., Ltd.; Inventors: Toshihiko Hanawa, Sumiko Hyuga, Hiroshi Odaguchi, Yukihiro Goda, Masashi Hyuga, Masashi Uema, Hiroshi Asakura, Nahoko Uchiyama, Yoshiaki Amakura, Morio Yoshimura, Jinwei Yang, and Kazuomi Mizoguchi.

## Figures and Tables

**Table 1:** Part 1: Adverse events/side effects by symptoms (analyzed population in Part 1).

	EFE Group (N = 5)	
Events	Number of Events	Number of Affected Participants (%)
Adverse events		
All events	6	2 (40.0)
Mental disorder	1	1 (20.0)
Initial insomnia	1	1 (20.0)
Gastrointestinal disorder	2	1 (20.0)
Constipation	1	1 (20.0)
Diarrhea	1	1 (20.0)
Clinical laboratory results	3	1 (20.0)
C-reactive protein increased	1	1 (20.0)
Neutrophil count increased	1	1 (20.0)
White blood cell count increased	1	1 (20.0)
Side effects		
All events	3	1 (20.0)
Clinical laboratory results	3	1 (20.0)
C-reactive protein increased	1	1 (20.0)
Neutrophil count increased	1	1 (20.0)
White blood cell count increased	1	1 (20.0)

**Table 2:** Primary endpoint: non-aggravation rate on Day 15.

Analyzed population: mITT					
Primary analysis					
Time	Group	Target number of participants	Number of participants with non-aggravation (%)	Two-sided 95% CI	Chi-square test
Day 15	EFE group	38	38 (100.0)	90.7–100.0	*p* = 0.146
	Placebo group	37	35 (94.6)	81.8–99.3	
Analyzed population: PPS					
Secondary analysis					
Time	Group	Target number of participants	Number of participants with non-aggravation (%)	Two-sided 95% CI	Chi-square test
Day 15	EFE group	33	33 (100.0)	89.4–100.0	*p* = 0.306
	Placebo group	32	31 (96.9)	83.8–99.9	

Target number of participants: excluding patients who used the prohibited concomitant drugs.

## Data Availability

The original contributions presented in this study are included in the article/Supplementary Materials. Further inquiries can be directed to the corresponding author.

## Supplementary Materials

The following supporting information can be downloaded at: https://www.mdpi.com/article/10.3390/microorganisms13030641/s1, Supplemental Information S1: Exclusion criteria; Supplemental Information S2: Details regarding the disease status score using the 6-point ordinal scale; Supplemental Information S3: Exclusion criteria for the PPS population; Figure S1: Face Scale (FS); Figure S2: Part 2: Secondary endpoint (3): Number of days until the body temperature is below 37.0 °C twice consecutively without using antipyretic analgesics (mITT); Figure S3: Part 2: Secondary endpoint (5): Number of days until the FS score for symptoms/pain symptoms becomes 0 or 1 (mITT); Table S1: Methods to confirm SARS-CoV-2 negative conversion; Table S2: Part 1: Population demographic data and other baseline characteristics (Analyzed population in Part 1); Table S3: Part 2: Population demographic data and other baseline characteristics (mITT); Table S4: Acceptance/rejection to the analysis population (participants included in Part 2); Table S5: Part 2: Secondary endpoint (1): SARS-CoV-2 negative conversation rate on Day 15; Table S6: Part 2: Secondary endpoint (2): Frequency of use of antipyretic analgesics, antitussives, and expectorants; Table S7: Part 2: Secondary endpoint (4): Degree of improvement in the FS scores for symptoms/pain symptoms (FS); Table S8: Part 2: Secondary endpoint (6): Non-aggravation rate on Day 21 (ratio of patients that ended the study without termination from the study due to the symptoms deteriorating and becoming moderate or severe during the study period: termination criterion 5) (mITT); Table S9: Part 2: Secondary endpoint (7): Ratio of patients without severe condition on Day 15, Day 21 (mITT); Table S10: Part 2: Secondary endpoint (8): ”Disease status score on a 6-point ordinal scale” on Day 7, Day 15/at termination, and Day 21/1 week after termination (mITT); Table S11: Adverse events/side effects by severity for each symptom; Table S12: Part 2: Changes in physical findings.
