# Supplementary material for: Safety and Efficacy of Ephedrine Alkaloids-Free Ephedra Herb Extract (EFE) for Mild COVID-19: A Double-Blind, Placebo-Controlled, Randomized Comparative Trial
Source: Microorganisms. 2025 Mar 12;13(3):641. doi: 10.3390/microorganisms13030641 (PMC11946151; doi:10.3390/microorganisms13030641)
Supplement: Supplementary file 1 [file microorganisms-13-00641-s001.zip › microorganisms-3530952-supplementary.pdf]

Supplemental Information S1 Exclusion criteria

(1) Individuals who have participated in other clinical trials within 12 weeks before the start of drug administration, or who plan to participate in clinical trials involving unapproved drugs during the study period.
(2) Individuals who have received COVID-19 treatment within 7 days before the start of drug administration.
(3) Individuals without symptoms of COVID-19 (such as fever or upper respiratory symptoms like cough).
(4) Individuals experiencing a recurrence or reinfection of SARS-CoV-2.
(5) Individuals who cannot discontinue the use of prohibited concomitant medications during the study period.
(6) Individuals with an SpO_2 <96% without oxygen therapy.
(7) Individuals with severe or uncontrolled underlying conditions [chronic obstructive pulmonary disease, chronic kidney disease, liver dysfunction, diabetes, hypertension, cardiovascular disease, malignant tumors, obesity with a body mass index (BMI) ≥30] (except if mild and controlled).
(8) Individuals with impaired consciousness, including disorientation.
(9) Individuals with hypersensitivity to ephedra or ephedra-containing Kampo formulations.
(10) Pregnant or breastfeeding individuals, or those who may be pregnant.
(11) For women of childbearing age, individuals who test positive on a pregnancy test and those who cannot agree to use contraception under the guidance of the clinical trial physician from the time of enrollment until the end of observation, for both men and women.
(12) Individuals with an implanted pacemaker.
(13) Individuals living with a family member who is a participant in this clinical trial, or who have a family member who was previously a participant in this clinical trial.
(14) Individuals considered ineligible by the study doctor for any other reason.

Figure S1: Face scale (FS)

A method for evaluating the participant's symptoms and pain intensity based on facial expressions. The participant is asked to choose the expression that most closely matches their feelings from the seven expressions below in order to assess their symptoms and pain.

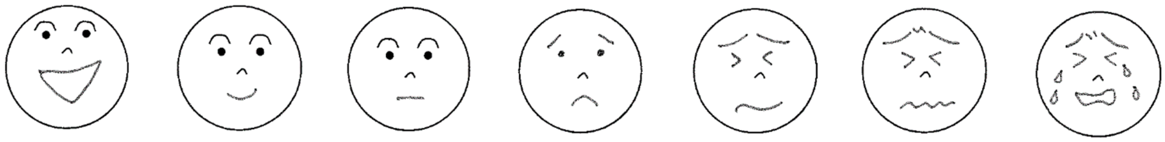

0 1 2 3 4 5 6

Supplemental Information S2 Details regarding the disease status score using the 6-point ordinal scale

1. Not hospitalized
2. Hospitalized, does not require oxygen supplementation
3. Hospitalized, requires oxygen supplementation
4. Hospitalized, with use of noninvasive artificial ventilation or high-flow oxygen therapy device
5. Hospitalized, with use of invasive artificial ventilation or ECMO
6. Fatal

Supplemental Information S3 Exclusion criteria for the PPS population

1) Corresponds to violation of exclusion criteria
2) Corresponds to violation of prohibited concomitant drug use
3) Corresponds to study termination criteria but did not terminate participation
4) Violation of the set dosage and administration/treatment period

Table S1 Methods to confirm SARS-CoV-2 negative conversion

Item name	Marketing authorization holder name	Test method
Loopamp COVID-19（SARS-CoV-2）detection reagent kit	Eiken Chemical Co., Ltd.	Nucleic acid amplification (LAMP)
Xpert Xpress SARS-CoV-2 “Cepheid” /Nucleic acid	Beckman Coulter K.K.	Nucleic acid amplification (RT-PCR)
Takara SARS-CoV-2 Direct PCR Detection Kit	Takara Bio Inc.	Nucleic acid amplification (RT-PCR)
cobas　SARS-CoV-2	Roche Diagnostics K.K.	Nucleic acid amplification (RT-PCR)

Table S2 Part 1：Population demographic data and other baseline characteristics (Analyzed population in Part 1)

Participant demographics	EFE group
Number of participants	5
Sex	
Male (%)	1 (20.0)
Female (%)	4 (80.0)
Age [y.o.]	
Mean (standard deviation)	35.0 (15.7)
Median	28.0
[Minimum, Maximum]	[27, 63]
Height [cm]	
Mean (standard deviation)	159.76 (9.34)
Median	157.20
[Minimum, Maximum]	[149.0, 173.9]
Body weight [kg]	
Mean (standard deviation)	56.10 (10.10)
Median	51.20
[Minimum, Maximum]	[49.0, 73.0]
BMI [kg/m^2]	
Mean (standard deviation)	21.96 (3.28)
Median	20.70
[Minimum, Maximum]	[19.1, 27.5]
Smoking history	
No (%)	5 (100.0)
Yes (%)	0 (0.0)
Drinking history	
No (%)	1 (20.0)
Yes (%)	4 (80.0)
Medical history	
No (%)	4 (80.0)
Yes (%)	1 (20.0)
Complications	
No (%)	1 (20.0)
Yes (%)	4 (80.0)
Allergies	
No (%)	2 (40.0)
Yes (%)	3 (60.0)
Family history	
No (%)	4 (80.0)

Participant demographics	EFE group
Yes (%)	1 (20.0)
Physical findings	
Systolic blood pressure [mmHg]	
Mean (standard deviation)	112.4 (10.2)
Median	115.0
[Minimum, Maximum]	[102, 125]
Diastolic blood pressure [mmHg]	
Mean (standard deviation)	74.0 (11.6)
Median	79.0
[Minimum, Maximum]	[61, 85]
Body temperature [℃]	
Mean (standard deviation)	37.06 (0.56)
Median	37.20
[Minimum, Maximum]	[36.3, 37.7]
Pulse [bpm]	
Mean (standard deviation)	83.5 (12.7)
Median	82.0
[Minimum, Maximum]	[65, 98]
SpO_2 [%]	
Mean (standard deviation)	98.30 (1.15)
Median	98.50
[Minimum, Maximum]	[96.5, 99.5]

The average values of two measurements were used for aggregation for the pulse and SpO_2.

Table S3 Part 2：Population demographic data and other baseline characteristics (mITT)

Participant demographics	EFE group	Placebo group	All
Number of participants	41	40	81
Sex			
Male	13 (31.7)	7 (17.5)	20 (24.7)
Female	28 (68.3)	33 (82.5)	61 (75.3)
Age [y.o.]			
Mean (standard deviation)	42.0 (11.8)	43.2 (11.2)	42.6 (11.4)
Median	42.0	41.5	42.0
[Minimum, Maximum]	[21, 69]	[22, 73]	[21, 73]
Height [cm]			
Mean (standard deviation)	161.74 (8.57)	161.10 (7.93)	161.42 (8.21)
Median	162.00	159.00	161.00
[Minimum, Maximum]	[142.5, 180.0]	[150.0, 180.4]	[142.5, 180.4]
Body weight [kg]			
Mean (standard deviation)	58.58 (11.17)	57.81 (10.20)	58.20 (10.64)
Median	57.00	57.10	57.00
[Minimum, Maximum]	[42.9, 86.4]	[43.2, 82.4]	[42.9, 86.4]
BMI[kg/m^2]			
Mean (standard deviation)	22.33 (3.53)	22.20 (2.98)	22.27 (3.25)
Median	21.40	21.55	21.50
[Minimum, Maximum]	[17.0, 32.4]	[16.8, 28.6]	[16.8, 32.4]
Smoking history			
No	36 (87.8)	32 (80.0)	68 (84.0)
Yes	5 (12.2)	8 (20.0)	13 (16.0)
Drinking history			
No	26 (63.4)	27 (67.5)	53 (65.4)
Yes	15 (36.6)	13 (32.5)	28 (34.6)
Medical history			
No	38 (92.7)	35 (87.5)	73 (90.1)
Yes	3 (7.3)	5 (12.5)	8 (9.9)
Complications			
No	18 (43.9)	19 (47.5)	37 (45.7)
Yes	23 (56.1)	21 (52.5)	44 (54.3)
Allergies			
No	37 (90.2)	39 (97.5)	76 (93.8)
Yes	4 (9.8)	1 (2.5)	5 (6.2)
Family history			
No	27 (65.9)	22 (55.0)	49 (60.5)
Yes	14 (34.1)	18 (45.0)	32 (39.5)
Physical findings			

Participant demographics	EFE group	Placebo group	All
Systolic blood pressure [mmHg]			
Mean (standard deviation)	118.2 (17.9)	122.3 (18.0)	120.2 (18.0)
Median	116.0	118.0	117.0
[Minimum, Maximum]	[84, 173]	[98, 165]	[84, 173]
Diastolic blood pressure [mmHg]			
Mean (standard deviation)	79.2 (12.2)	80.0 (14.8)	79.6 (13.5)
Median	78.0	79.0	78.0
[Minimum, Maximum]	[58, 107]	[57, 115]	[57, 115]
Body temperature [℃]			
Mean (standard deviation)	36.66 (0.35)	36.78 (0.77)	36.72 (0.60)
Median	36.70	36.65	36.70
[Minimum, Maximum]	[35.6, 37.6]	[35.6, 39.9]	[35.6, 39.9]
Pulse [bpm]			
Mean (standard deviation)	81.8 (11.7)	83.8 (12.7)	82.8 (12.2)
Median	80.0	85.0	82.0
[Minimum, Maximum]	[55, 105]	[56, 114]	[55, 114]
SpO_2 [%]			
Mean (standard deviation)	98.23 (0.63)	98.20 (0.67)	98.22 (0.65)
Median	98.50	98.00	98.00
[Minimum, Maximum]	[96.5, 99.5]	[97.0, 99.5]	[96.5, 99.5]

The average values of two measurements were used for aggregation for the pulse and SpO_2.

Table S4 Acceptance/rejection to the analysis population (participants included in Part 2)

	EFE group (N=41) n (%)	Placebo group (N=40) n (%)	All (N=81) n (%)
mITT accepted	41 (100.0)	40 (100.0)	81 (100.0)
mITT rejected	0 (0.0)	0 (0.0)	0 (0.0)
Participants not receiving a single dose of study drug	0	0	0
GCP non-compliant participants	0	0	0
Other	0	0	0
PPS accepted	33 (80.5)	32 (80.0)	65 (80.2)
PPS rejected	8 (19.5)	8 (20.0)	16 (19.8)
Participants not receiving a single dose of the study drug	0	0	0
Participants with "No" as the response to "Have all eligibility criteria been met?" in the EDC "Non-compliance – Inclusion/exclusion criteria"	0	0	0
Participants that used prohibited concomitant drugs	3	3	6
Participants violating the treatment period setting of study drug	5	7	12
Participants deviating from the study schedule (allowance)	0	0	0
Participants who did not terminate study participation despite the condition becoming moderate or more	0	0	0
GCP non-compliant participants	0	0	0
Other	0	1	1
Safety analysis population accepted	41 (100.0)	40 (100.0)	81 (100.0)
Safety analysis population rejected	0 (0.0)	0 (0.0)	0 (0.0)
Participants not receiving a single dose of study drug	0	0	0
GCP non-compliant participants	0	0	0
Other	0	0	0

Reason for rejection are counted double.

Table S5　Part 2：Secondary endpoint (1): SARS-CoV-2 negative conversation rate on Day 15

Time	Group	Target number of participants	Number of participants negative for SARS-CoV-2 (%)	Two-sided 95% confidence interval	Chi-squared test
Day 15	EFE group	38	30 (78.9)	62.7–90.4	p=0.591
	Placebo group	37	31 (83.8)	68.0–93.8	

Target number of participants：Excluding patients who used the prohibited concomitant drugs

Table S6 Part 2 : Secondary endpoint (2): Frequency of use of antipyretic analgesics, antitussives, and expectorants

Item	EFE group (N=41)	Placebo group (N=40)	Test*
Target number of participants	38	37	
Antipyretic analgesics			
Usage rate			
Number of participants	24	21	p=0.572
% [two-sided 95% CI]	63.2 [46.0–78.2]	56.8 [39.5–72.9]	
Total number of uses			
Number of participants	38	37	p=0.591
Mean (standard deviation)	3.4 (4.6)	3.4 (6.0)	
Median	2.0	2.0	
[Minimum, Maximum]	[0, 18]	[0, 29]	
Antitussive agents			
Usage rate			
Number of participants	19	13	p=0.193
% [two-sided 95% CI]	50.0 [33.4–66.6]	35.1 [20.2–52.5]	
Total number of uses			
Number of participants	38	37	p=0.118
Mean (standard deviation)	7.8 (10.9)	4.1 (7.2)	
Median	1.0	0.0	
[Minimum, Maximum]	[0, 41]	[0, 27]	
Expectorant			
Usage rate			
Number of participants	16	14	p=0.706
% [two-sided 95% CI]	42.1 [26.3–59.2]	37.8 [22.5–55.2]	
Total number of uses			
Number of participants	38	37	p=0.684
Mean (standard deviation)	6.7 (10.9)	5.6 (9.3)	
Median	0.0	0.0	
[Minimum, Maximum]	[0, 38]	[0, 39]	

Target number of participants : Excluding patients who used the prohibited concomitant drugs
* : Chi-square test or Wilcoxon rank sum test

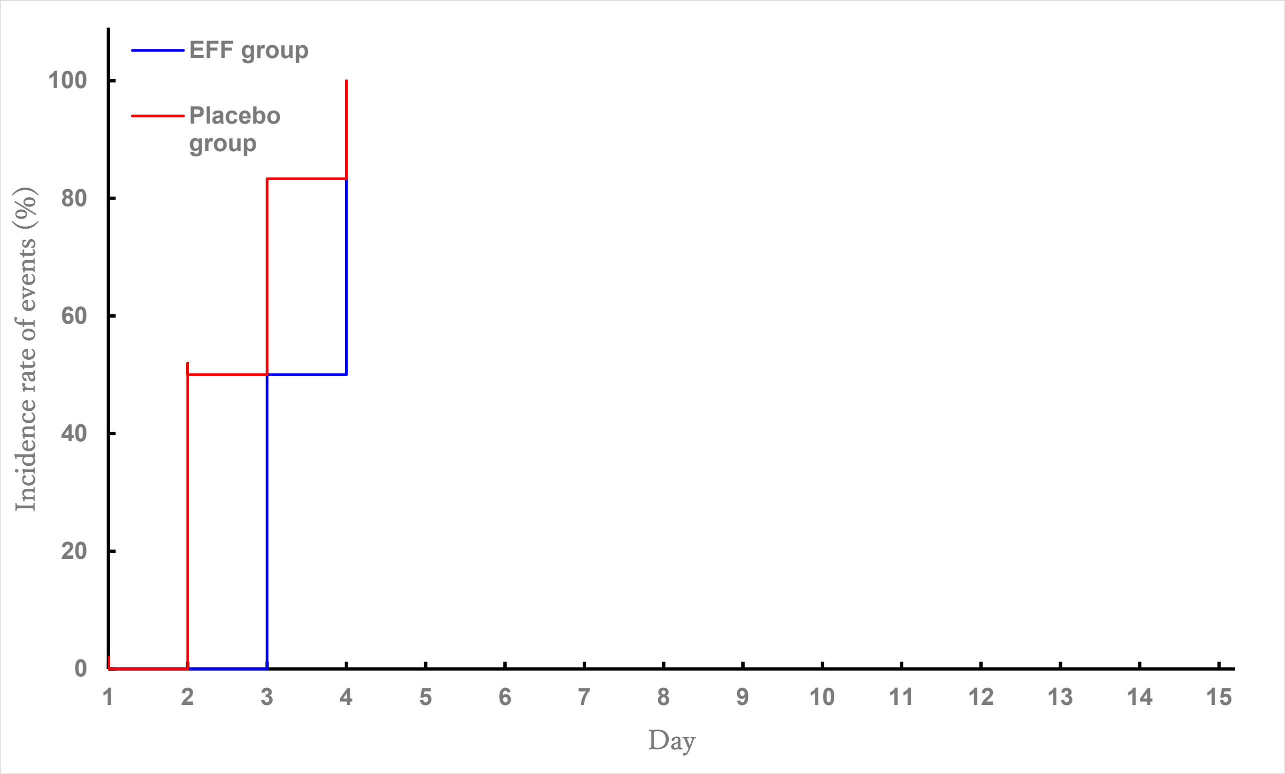

Figure S2 : Part 2 : Secondary endpoint (3) : Number of days until the body temperature is below 37.0°C twice consecutively without using antipyretic analgesics (mITT)

Table S7： Part 2：Secondary endpoint (4): Degree of improvement in the FS scores for symptoms/pain symptoms (FS)

Malaise

Time	EFE group (N=41)		Placebo group (N=40)		Wilcoxon rank sum test
	Measurement	Change	Measurement	Change	
Day 1 First time					
Number of participants	41		37		
Mean (standard deviation)	2.3 (1.4)		3.0 (1.5)		
Median	2.0		3.0		
[Minimum, Maximum]	[0, 5]		[0, 6]		
Day 1 Second time					
Number of participants	41	41	35	35	p=0.504
Mean (standard deviation)	2.1 (1.3)	-0.2 (1.2)	2.3 (1.4)	-0.7 (1.0)	
Median	2.0	0.0	2.0	0.0	
[Minimum, Maximum]	[0, 5]	[-4, 3]	[0, 6]	[-3, 1]	
Day 2 First time					
Number of participants	41	41	38	36	p=0.531
Mean (standard deviation)	1.8 (1.2)	-0.5 (1.1)	2.0 (1.2)	-1.0 (1.1)	
Median	2.0	-1.0	2.0	-1.0	
[Minimum, Maximum]	[0, 5]	[-3, 3]	[0, 5]	[-3, 1]	
Day 2 Second time					
Number of participants	39	39	37	35	p=0.117
Mean (standard deviation)	1.5 (1.2)	-0.8 (1.0)	2.1 (1.5)	-0.9 (1.3)	
Median	1.0	-1.0	2.0	-1.0	
[Minimum, Maximum]	[0, 4]	[-3, 2]	[0, 6]	[-3, 3]	

Malaise

Time	EFE group (N=41)		Placebo group (N=40)		Wilcoxon rank sum test
	Measurement	Change	Measurement	Change	
Day3 First time					
Number of participants	40	40	38	36	p=0.124
Mean (standard deviation)	1.3 (1.2)	-1.1 (1.2)	1.8 (1.4)	-1.2 (1.3)	
Median	1.0	-1.0	1.5	-1.0	
[Minimum, Maximum]	[0, 5]	[-5, 2]	[0, 5]	[-4, 2]	
Day3 Second time					
Number of participants	39	39	36	35	p=0.411
Mean (standard deviation)	1.2 (1.1)	-1.2 (1.2)	1.3 (1.1)	-1.6 (1.4)	
Median	1.0	-1.0	1.0	-2.0	
[Minimum, Maximum]	[0, 3]	[-4, 1]	[0, 4]	[-4, 2]	
Day4 First time					
Number of participants	40	40	39	36	p=0.017
Mean (standard deviation)	0.8 (0.9)	-1.6 (1.2)	1.4 (1.2)	-1.6 (1.5)	
Median	1.0	-1.0	1.0	-2.0	
[Minimum, Maximum]	[0, 4]	[-5, 1]	[0, 4]	[-4, 2]	
Day4 Second time					
Number of participants	39	39	39	36	p=0.025
Mean (standard deviation)	0.7 (0.9)	-1.7 (1.2)	1.2 (1.2)	-1.8 (1.7)	
Median	0.0	-2.0	1.0	-2.0	
[Minimum, Maximum]	[0, 4]	[-5, 0]	[0, 5]	[-6, 3]	

Malaise

Time	EFE group (N=41)		Placebo group (N=40)		Wilcoxon rank sum test
	Measurement	Change	Measurement	Change	
Day5 First time					
Number of participants	40	40	39	36	p=0.047
Mean (standard deviation)	0.7 (0.8)	-1.7 (1.3)	1.2 (1.2)	-1.9 (1.6)	
Median	0.0	-2.0	1.0	-2.0	
[Minimum, Maximum]	[0, 3]	[-5, 1]	[0, 5]	[-5, 3]	
Day5 Second time					
Number of participants	39	39	36	33	p=0.278
Mean (standard deviation)	0.8 (1.0)	-1.6 (1.2)	1.0 (1.0)	-2.0 (1.6)	
Median	0.0	-2.0	1.0	-2.0	
[Minimum, Maximum]	[0, 3]	[-5, 1]	[0, 4]	[-5, 2]	
Day6 First time					
Number of participants	40	40	39	36	p=0.147
Mean (standard deviation)	0.7 (1.0)	-1.7 (1.3)	1.1 (1.2)	-1.9 (1.6)	
Median	0.0	-2.0	1.0	-2.0	
[Minimum, Maximum]	[0, 3]	[-5, 1]	[0, 4]	[-5, 2]	
Day6 Second time					
Number of participants	38	38	36	34	p=0.148
Mean (standard deviation)	0.6 (0.8)	-1.8 (1.3)	0.9 (0.9)	-2.0 (1.6)	
Median	0.0	-2.0	1.0	-2.0	
[Minimum, Maximum]	[0, 2]	[-5, 0]	[0, 4]	[-5, 2]	

Malaise

Time	EFE group (N=41)		Placebo group (N=40)		Wilcoxon rank sum test
	Measurement	Change	Measurement	Change	
Day7 First time					
Number of participants	40	40	39	36	p=0.135
Mean (standard deviation)	0.5 (0.8)	-1.9 (1.2)	0.8 (1.0)	-2.2 (1.6)	
Median	0.0	-2.0	1.0	-2.0	
[Minimum, Maximum]	[0, 3]	[-5, 1]	[0, 4]	[-6, 1]	
Day7 Second time					
Number of participants	40	40	36	33	p=0.499
Mean (standard deviation)	0.5 (0.8)	-1.9 (1.3)	0.6 (0.8)	-2.3 (1.7)	
Median	0.0	-2.0	0.0	-3.0	
[Minimum, Maximum]	[0, 3]	[-5, 1]	[0, 3]	[-6, 1]	
Day8 First time					
Number of participants	39	39	36	33	p=0.395
Mean (standard deviation)	0.6 (0.9)	-1.8 (1.4)	0.7 (0.9)	-2.2 (1.6)	
Median	0.0	-2.0	0.5	-3.0	
[Minimum, Maximum]	[0, 3]	[-5, 2]	[0, 4]	[-5, 1]	
Day8 Second time					
Number of participants	37	37	35	33	p=0.733
Mean (standard deviation)	0.4 (0.7)	-2.0 (1.4)	0.4 (0.7)	-2.5 (1.6)	
Median	0.0	-2.0	0.0	-3.0	
[Minimum, Maximum]	[0, 2]	[-5, 1]	[0, 3]	[-6, 0]	

Malaise

Time	EFE group (N=41)		Placebo group (N=40)		Wilcoxon rank sum test
	Measurement	Change	Measurement	Change	
Day9 First time					
Number of participants	39	39	37	35	p=0.557
Mean (standard deviation)	0.4 (0.7)	-2.0 (1.3)	0.5 (0.9)	-2.4 (1.6)	
Median	0.0	-2.0	0.0	-3.0	
[Minimum, Maximum]	[0, 3]	[-5, 1]	[0, 4]	[-6, 0]	
Day9 Second time					
Number of participants	37	37	37	35	p=0.660
Mean (standard deviation)	0.4 (0.7)	-2.1 (1.3)	0.4 (0.9)	-2.5 (1.7)	
Median	0.0	-2.0	0.0	-3.0	
[Minimum, Maximum]	[0, 2]	[-5, 0]	[0, 4]	[-6, 0]	
Day10 First time					
Number of participants	38	38	38	35	p=0.595
Mean (standard deviation)	0.4 (0.8)	-2.0 (1.3)	0.5 (0.9)	-2.5 (1.7)	
Median	0.0	-2.0	0.0	-3.0	
[Minimum, Maximum]	[0, 3]	[-5, 1]	[0, 3]	[-6, 1]	
Day10 Second time					
Number of participants	36	36	36	33	p=0.964
Mean (standard deviation)	0.4 (0.8)	-2.0 (1.4)	0.4 (0.8)	-2.5 (1.8)	
Median	0.0	-2.0	0.0	-3.0	
[Minimum, Maximum]	[0, 4]	[-5, 1]	[0, 3]	[-6, 1]	

Malaise

Time	EFE group (N=41)		Placebo group (N=40)		Wilcoxon rank sum test
	Measurement	Change	Measurement	Change	
Day11 First time					
Number of participants	39	39	35	33	p=0.708
Mean (standard deviation)	0.4 (0.7)	-2.0 (1.3)	0.4 (0.8)	-2.6 (1.6)	
Median	0.0	-2.0	0.0	-3.0	
[Minimum, Maximum]	[0, 3]	[-5, 0]	[0, 3]	[-6, 0]	
Day11 Second time					
Number of participants	34	34	34	32	p=0.960
Mean (standard deviation)	0.4 (0.8)	-1.9 (1.1)	0.4 (0.7)	-2.6 (1.8)	
Median	0.0	-2.0	0.0	-3.0	
[Minimum, Maximum]	[0, 3]	[-4, 0]	[0, 3]	[-6, 2]	
Day12 First time					
Number of participants	38	38	35	33	p=0.768
Mean (standard deviation)	0.4 (0.8)	-1.9 (1.2)	0.5 (0.9)	-2.5 (1.8)	
Median	0.0	-2.0	0.0	-3.0	
[Minimum, Maximum]	[0, 4]	[-4, 0]	[0, 3]	[-6, 1]	
Day12 Second time					
Number of participants	34	34	33	31	p=0.507
Mean (standard deviation)	0.2 (0.6)	-2.0 (1.3)	0.3 (0.6)	-2.7 (1.6)	
Median	0.0	-2.0	0.0	-3.0	
[Minimum, Maximum]	[0, 2]	[-5, 0]	[0, 2]	[-6, 1]	

Malaise

Time	EFE group (N=41)		Placebo group (N=40)		Wilcoxon rank sum test
	Measurement	Change	Measurement	Change	
Day13 First time					
Number of participants	38	38	36	33	p=0.367
Mean (standard deviation)	0.3 (0.7)	-2.0 (1.3)	0.4 (0.8)	-2.5 (1.6)	
Median	0.0	-2.0	0.0	-3.0	
[Minimum, Maximum]	[0, 3]	[-5, 0]	[0, 3]	[-6, 0]	
Day13 Second time					
Number of participants	35	35	33	31	p=0.411
Mean (standard deviation)	0.2 (0.6)	-2.1 (1.3)	0.4 (0.8)	-2.6 (1.7)	
Median	0.0	-2.0	0.0	-3.0	
[Minimum, Maximum]	[0, 3]	[-5, 0]	[0, 3]	[-6, 1]	
Day14 First time					
Number of participants	39	39	34	32	p=0.331
Mean (standard deviation)	0.2 (0.5)	-2.2 (1.3)	0.4 (0.9)	-2.6 (1.6)	
Median	0.0	-2.0	0.0	-2.5	
[Minimum, Maximum]	[0, 3]	[-5, 0]	[0, 4]	[-6, 0]	
Day14 Second time					
Number of participants	35	35	34	32	p=0.699
Mean (standard deviation)	0.2 (0.6)	-2.0 (1.3)	0.3 (0.7)	-2.6 (1.6)	
Median	0.0	-2.0	0.0	-3.0	
[Minimum, Maximum]	[0, 3]	[-5, 0]	[0, 3]	[-6, 0]	

Malaise

Time	EFE group (N=41)		Placebo group (N=40)		Wilcoxon rank sum test
	Measurement	Change	Measurement	Change	
Day15 First time					
Number of participants	37	37	34	31	p=0.220
Mean (standard deviation)	0.1 (0.3)	-2.2 (1.4)	0.4 (0.8)	-2.6 (1.7)	
Median	0.0	-2.0	0.0	-3.0	
[Minimum, Maximum]	[0, 1]	[-5, 0]	[0, 4]	[-6, 0]	

Chills

Time	EFE group (N=41)		Placebo group (N=40)		Wilcoxon rank sum test
	Measurement	Change	Measurement	Change	
Day1 First time					
Number of participants	41		37		
Mean (standard deviation)	1.2 (1.5)		1.3 (1.7)		
Median	1.0		1.0		
[Minimum, Maximum]	[0, 5]		[0, 6]		
Day1 Second time					
Number of participants	41	41	35	35	p=0.535
Mean (standard deviation)	1.1 (1.3)	-0.1 (1.4)	1.0 (1.5)	-0.3 (0.7)	
Median	1.0	0.0	0.0	0.0	
[Minimum, Maximum]	[0, 5]	[-3, 4]	[0, 6]	[-2, 1]	
Day2 First time					
Number of participants	41	41	38	36	p=0.663
Mean (standard deviation)	0.7 (1.2)	-0.5 (1.4)	0.7 (1.1)	-0.7 (1.4)	
Median	0.0	0.0	0.0	0.0	
[Minimum, Maximum]	[0, 5]	[-3, 3]	[0, 5]	[-6, 1]	
Day2 Second time					
Number of participants	39	39	37	35	p=0.063
Mean (standard deviation)	0.4 (0.9)	-0.8 (1.1)	0.9 (1.2)	-0.5 (1.7)	
Median	0.0	0.0	0.0	0.0	
[Minimum, Maximum]	[0, 3]	[-4, 1]	[0, 4]	[-6, 4]	

Chills

Time	EFE group (N=41)		Placebo group (N=40)		Wilcoxon rank sum test
	Measurement	Change	Measurement	Change	
Day3 First time					
Number of participants	40	40	38	36	p=0.380
Mean (standard deviation)	0.4 (0.8)	-0.8 (1.2)	0.6 (1.0)	-0.8 (1.6)	
Median	0.0	0.0	0.0	0.0	
[Minimum, Maximum]	[0, 3]	[-3, 1]	[0, 4]	[-6, 3]	
Day3 Second time					
Number of participants	39	39	36	35	p=0.984
Mean (standard deviation)	0.4 (0.7)	-0.8 (1.4)	0.4 (0.6)	-0.9 (1.7)	
Median	0.0	0.0	0.0	0.0	
[Minimum, Maximum]	[0, 2]	[-4, 2]	[0, 3]	[-6, 3]	
Day4 First time					
Number of participants	40	40	39	36	p=0.535
Mean (standard deviation)	0.2 (0.6)	-1.0 (1.3)	0.3 (0.8)	-0.9 (1.8)	
Median	0.0	0.0	0.0	-0.5	
[Minimum, Maximum]	[0, 3]	[-4, 1]	[0, 4]	[-6, 3]	
Day4 Second time					
Number of participants	39	39	39	36	p=0.313
Mean (standard deviation)	0.3 (0.7)	-0.9 (1.4)	0.4 (0.8)	-0.9 (1.7)	
Median	0.0	0.0	0.0	0.0	
[Minimum, Maximum]	[0, 3]	[-4, 2]	[0, 4]	[-6, 2]	

Chills

Time	EFE group (N=41)		Placebo group (N=40)		Wilcoxon rank sum test
	Measurement	Change	Measurement	Change	
Day5 First time					
Number of participants	40	40	39	36	p=0.362
Mean (standard deviation)	0.2 (0.5)	-1.0 (1.4)	0.3 (0.7)	-0.9 (1.7)	
Median	0.0	0.0	0.0	0.0	
[Minimum, Maximum]	[0, 2]	[-4, 1]	[0, 3]	[-6, 2]	
Day5 Second time					
Number of participants	39	39	36	33	p=0.111
Mean (standard deviation)	0.2 (0.5)	-1.1 (1.4)	0.3 (0.7)	-0.9 (1.6)	
Median	0.0	0.0	0.0	0.0	
[Minimum, Maximum]	[0, 2]	[-4, 1]	[0, 3]	[-6, 1]	
Day6 First time					
Number of participants	40	40	39	36	p=0.744
Mean (standard deviation)	0.2 (0.5)	-1.0 (1.4)	0.2 (0.5)	-1.1 (1.7)	
Median	0.0	0.0	0.0	0.0	
[Minimum, Maximum]	[0, 2]	[-4, 1]	[0, 2]	[-6, 1]	
Day6 Second time					
Number of participants	38	38	36	34	p=0.741
Mean (standard deviation)	0.2 (0.6)	-1.1 (1.4)	0.1 (0.5)	-1.1 (1.7)	
Median	0.0	0.0	0.0	0.0	
[Minimum, Maximum]	[0, 2]	[-4, 1]	[0, 3]	[-6, 1]	

Chills

Time	EFE group (N=41)		Placebo group (N=40)		Wilcoxon rank sum test
	Measurement	Change	Measurement	Change	
Day7 First time					
Number of participants	40	40	39	36	p=0.129
Mean (standard deviation)	0.2 (0.6)	-1.1 (1.4)	0.2 (0.4)	-1.1 (1.7)	
Median	0.0	0.0	0.0	-0.5	
[Minimum, Maximum]	[0, 3]	[-4, 1]	[0, 1]	[-6, 1]	
Day7 Second time					
Number of participants	40	40	36	33	p=0.735
Mean (standard deviation)	0.1 (0.5)	-1.1 (1.4)	0.1 (0.4)	-1.2 (1.7)	
Median	0.0	0.0	0.0	0.0	
[Minimum, Maximum]	[0, 2]	[-4, 1]	[0, 2]	[-6, 1]	
Day8 First time					
Number of participants	39	39	36	33	p=0.641
Mean (standard deviation)	0.1 (0.6)	-1.1 (1.4)	0.1 (0.3)	-1.1 (1.7)	
Median	0.0	0.0	0.0	0.0	
[Minimum, Maximum]	[0, 3]	[-4, 0]	[0, 1]	[-6, 1]	
Day8 Second time					
Number of participants	37	37	35	33	p=0.250
Mean (standard deviation)	0.1 (0.6)	-1.2 (1.4)	0.2 (0.5)	-1.0 (1.7)	
Median	0.0	-1.0	0.0	0.0	
[Minimum, Maximum]	[0, 3]	[-4, 0]	[0, 2]	[-6, 1]	

Chills

Time	EFE group (N=41)		Placebo group (N=40)		Wilcoxon rank sum test
	Measurement	Change	Measurement	Change	
Day9 First time					
Number of participants	39	39	37	35	p=0.961
Mean (standard deviation)	0.2 (0.6)	-1.1 (1.3)	0.2 (0.5)	-1.1 (1.7)	
Median	0.0	0.0	0.0	0.0	
[Minimum, Maximum]	[0, 3]	[-4, 1]	[0, 2]	[-6, 2]	
Day9 Second time					
Number of participants	37	37	37	35	p=0.508
Mean (standard deviation)	0.1 (0.5)	-1.1 (1.4)	0.2 (0.4)	-1.1 (1.7)	
Median	0.0	0.0	0.0	-1.0	
[Minimum, Maximum]	[0, 2]	[-4, 1]	[0, 2]	[-6, 2]	
Day10 First time					
Number of participants	38	38	38	35	p=0.501
Mean (standard deviation)	0.2 (0.6)	-1.1 (1.4)	0.2 (0.6)	-1.1 (1.7)	
Median	0.0	-0.5	0.0	0.0	
[Minimum, Maximum]	[0, 3]	[-4, 1]	[0, 3]	[-6, 1]	
Day10 Second time					
Number of participants	36	36	36	33	p=0.910
Mean (standard deviation)	0.2 (0.7)	-1.0 (1.4)	0.1 (0.3)	-1.2 (1.8)	
Median	0.0	0.0	0.0	0.0	
[Minimum, Maximum]	[0, 3]	[-4, 1]	[0, 1]	[-6, 1]	

Chills

Time	EFE group (N=41)		Placebo group (N=40)		Wilcoxon rank sum test
	Measurement	Change	Measurement	Change	
Day11 First time					
Number of participants	39	39	35	33	p=0.954
Mean (standard deviation)	0.2 (0.6)	-1.1 (1.3)	0.1 (0.3)	-1.2 (1.8)	
Median	0.0	0.0	0.0	0.0	
[Minimum, Maximum]	[0, 3]	[-4, 0]	[0, 1]	[-6, 1]	
Day11 Second time					
Number of participants	34	34	34	32	p=0.593
Mean (standard deviation)	0.1 (0.3)	-1.1 (1.4)	0.1 (0.2)	-1.2 (1.8)	
Median	0.0	-0.5	0.0	0.0	
[Minimum, Maximum]	[0, 2]	[-4, 0]	[0, 1]	[-6, 1]	
Day12 First time					
Number of participants	38	38	35	33	p=0.605
Mean (standard deviation)	0.1 (0.5)	-1.1 (1.4)	0.1 (0.6)	-1.1 (1.9)	
Median	0.0	0.0	0.0	0.0	
[Minimum, Maximum]	[0, 2]	[-4, 0]	[0, 3]	[-6, 3]	
Day12 Second time					
Number of participants	34	34	33	31	p=0.425
Mean (standard deviation)	0.1 (0.5)	-1.1 (1.4)	0.2 (0.4)	-1.2 (1.7)	
Median	0.0	0.0	0.0	-1.0	
[Minimum, Maximum]	[0, 2]	[-4, 0]	[0, 2]	[-6, 1]	

Chills

Time	EFE group (N=41)		Placebo group (N=40)		Wilcoxon rank sum test
	Measurement	Change	Measurement	Change	
Day13 First time					
Number of participants	38	38	36	33	p=0.246
Mean (standard deviation)	0.1 (0.6)	-1.1 (1.3)	0.2 (0.5)	-1.1 (1.7)	
Median	0.0	0.0	0.0	0.0	
[Minimum, Maximum]	[0, 3]	[-4, 0]	[0, 2]	[-6, 1]	
Day13 Second time					
Number of participants	35	35	33	31	p=0.306
Mean (standard deviation)	0.1 (0.5)	-1.0 (1.3)	0.2 (0.5)	-1.1 (1.8)	
Median	0.0	0.0	0.0	0.0	
[Minimum, Maximum]	[0, 3]	[-4, 0]	[0, 2]	[-6, 2]	
Day14 First time					
Number of participants	39	39	34	32	p=0.268
Mean (standard deviation)	0.1 (0.5)	-1.2 (1.3)	0.1 (0.3)	-1.1 (1.8)	
Median	0.0	-1.0	0.0	0.0	
[Minimum, Maximum]	[0, 3]	[-4, 0]	[0, 1]	[-6, 1]	
Day14 Second time					
Number of participants	35	35	34	32	p=0.321
Mean (standard deviation)	0.1 (0.5)	-1.1 (1.4)	0.1 (0.3)	-1.1 (1.7)	
Median	0.0	0.0	0.0	0.0	
[Minimum, Maximum]	[0, 3]	[-4, 0]	[0, 1]	[-6, 1]	

Chills

Time	EFE group (N=41)		Placebo group (N=40)		Wilcoxon rank sum test
	Measurement	Change	Measurement	Change	
Day15 First time					
Number of participants	37	37	34	31	p=0.143
Mean (standard deviation)	0.0 (0.0)	-1.2 (1.4)	0.1 (0.2)	-1.1 (1.8)	
Median	0.0	-1.0	0.0	0.0	
[Minimum, Maximum]	[0, 0]	[-4, 0]	[0, 1]	[-6, 1]	

Shortness of breath

Time	EFE group (N=41)		Placebo group (N=40)		Wilcoxon rank sum test
	Measurement	Change	Measurement	Change	
Day1 First time					
Number of participants	41		37		
Mean (standard deviation)	1.3 (1.5)		1.1 (1.2)		
Median	1.0		1.0		
[Minimum, Maximum]	[0, 5]		[0, 4]		
Day1 Second time					
Number of participants	41	41	35	35	p=0.899
Mean (standard deviation)	1.1 (1.4)	-0.1 (0.9)	1.1 (1.1)	0.0 (1.1)	
Median	1.0	0.0	1.0	0.0	
[Minimum, Maximum]	[0, 5]	[-2, 2]	[0, 4]	[-4, 2]	
Day2 First time					
Number of participants	41	41	38	36	p=0.792
Mean (standard deviation)	0.9 (1.3)	-0.3 (1.0)	0.7 (0.8)	-0.3 (1.0)	
Median	0.0	0.0	0.5	0.0	
[Minimum, Maximum]	[0, 4]	[-3, 3]	[0, 3]	[-4, 1]	
Day2 Second time					
Number of participants	39	39	37	35	p=0.732
Mean (standard deviation)	0.8 (1.2)	-0.4 (0.8)	0.6 (0.9)	-0.4 (1.2)	
Median	0.0	0.0	0.0	0.0	
[Minimum, Maximum]	[0, 4]	[-3, 1]	[0, 4]	[-4, 3]	

Shortness of breath

Time	EFE group (N=41)		Placebo group (N=40)		Wilcoxon rank sum test
	Measurement	Change	Measurement	Change	
Day3 First time					
Number of participants	40	40	38	36	p=1.000
Mean (standard deviation)	0.6 (1.1)	-0.7 (1.0)	0.6 (1.1)	-0.4 (1.3)	
Median	0.0	0.0	0.0	0.0	
[Minimum, Maximum]	[0, 4]	[-4, 1]	[0, 4]	[-4, 3]	
Day3 Second time					
Number of participants	39	39	36	35	p=0.362
Mean (standard deviation)	0.8 (1.1)	-0.4 (0.9)	0.6 (0.9)	-0.5 (1.2)	
Median	0.0	0.0	0.0	0.0	
[Minimum, Maximum]	[0, 4]	[-3, 1]	[0, 3]	[-4, 2]	
Day4 First time					
Number of participants	40	40	39	36	p=0.878
Mean (standard deviation)	0.6 (0.9)	-0.7 (1.0)	0.5 (0.8)	-0.5 (1.3)	
Median	0.0	0.0	0.0	0.0	
[Minimum, Maximum]	[0, 3]	[-3, 1]	[0, 4]	[-4, 4]	
Day4 Second time					
Number of participants	39	39	39	36	p=0.966
Mean (standard deviation)	0.5 (1.0)	-0.7 (1.1)	0.5 (0.9)	-0.5 (1.3)	
Median	0.0	0.0	0.0	0.0	
[Minimum, Maximum]	[0, 4]	[-3, 1]	[0, 4]	[-4, 3]	

Shortness of breath

Time	EFE group (N=41)		Placebo group (N=40)		Wilcoxon rank sum test
	Measurement	Change	Measurement	Change	
Day5 First time					
Number of participants	40	40	39	36	p=0.939
Mean (standard deviation)	0.5 (0.9)	-0.8 (1.1)	0.5 (0.9)	-0.5 (1.3)	
Median	0.0	0.0	0.0	0.0	
[Minimum, Maximum]	[0, 3]	[-3, 1]	[0, 3]	[-4, 3]	
Day5 Second time					
Number of participants	39	39	36	33	p=0.650
Mean (standard deviation)	0.6 (0.9)	-0.7 (1.1)	0.5 (0.9)	-0.4 (1.3)	
Median	0.0	0.0	0.0	0.0	
[Minimum, Maximum]	[0, 3]	[-4, 1]	[0, 3]	[-4, 3]	
Day6 First time					
Number of participants	40	40	39	36	p=0.968
Mean (standard deviation)	0.6 (0.9)	-0.7 (1.1)	0.6 (1.1)	-0.4 (1.5)	
Median	0.0	0.0	0.0	0.0	
[Minimum, Maximum]	[0, 3]	[-3, 2]	[0, 5]	[-4, 5]	
Day6 Second time					
Number of participants	38	38	36	34	p=0.793
Mean (standard deviation)	0.5 (0.9)	-0.9 (1.3)	0.4 (0.6)	-0.7 (1.2)	
Median	0.0	-0.5	0.0	0.0	
[Minimum, Maximum]	[0, 3]	[-4, 1]	[0, 2]	[-4, 1]	

Shortness of breath

Time	EFE group (N=41)		Placebo group (N=40)		Wilcoxon rank sum test
	Measurement	Change	Measurement	Change	
Day7 First time					
Number of participants	40	40	39	36	p=0.506
Mean (standard deviation)	0.4 (0.8)	-0.9 (1.3)	0.5 (0.9)	-0.5 (1.4)	
Median	0.0	0.0	0.0	0.0	
[Minimum, Maximum]	[0, 3]	[-4, 1]	[0, 4]	[-4, 4]	
Day7 Second time					
Number of participants	40	40	36	33	p=0.575
Mean (standard deviation)	0.5 (0.9)	-0.8 (1.2)	0.4 (0.6)	-0.7 (1.3)	
Median	0.0	0.0	0.0	0.0	
[Minimum, Maximum]	[0, 3]	[-4, 1]	[0, 2]	[-4, 1]	
Day8 First time					
Number of participants	39	39	36	33	p=0.696
Mean (standard deviation)	0.4 (0.8)	-0.8 (1.2)	0.3 (0.6)	-0.8 (1.3)	
Median	0.0	0.0	0.0	0.0	
[Minimum, Maximum]	[0, 3]	[-4, 1]	[0, 2]	[-4, 1]	
Day8 Second time					
Number of participants	37	37	35	33	p=0.608
Mean (standard deviation)	0.5 (0.9)	-0.8 (1.3)	0.3 (0.6)	-0.7 (1.2)	
Median	0.0	0.0	0.0	0.0	
[Minimum, Maximum]	[0, 4]	[-4, 2]	[0, 2]	[-4, 1]	

Shortness of breath

Time	EFE group (N=41)		Placebo group (N=40)		Wilcoxon rank sum test
	Measurement	Change	Measurement	Change	
Day9 First time					
Number of participants	39	39	37	35	p=0.743
Mean (standard deviation)	0.4 (0.8)	-0.8 (1.2)	0.3 (0.6)	-0.7 (1.2)	
Median	0.0	0.0	0.0	0.0	
[Minimum, Maximum]	[0, 3]	[-3, 1]	[0, 2]	[-4, 1]	
Day9 Second time					
Number of participants	37	37	37	35	p=0.746
Mean (standard deviation)	0.4 (0.7)	-0.9 (1.3)	0.3 (0.6)	-0.7 (1.2)	
Median	0.0	0.0	0.0	0.0	
[Minimum, Maximum]	[0, 3]	[-4, 1]	[0, 2]	[-4, 1]	
Day10 First time					
Number of participants	38	38	38	35	p=0.525
Mean (standard deviation)	0.4 (0.8)	-0.8 (1.2)	0.2 (0.5)	-0.8 (1.3)	
Median	0.0	0.0	0.0	0.0	
[Minimum, Maximum]	[0, 3]	[-4, 1]	[0, 2]	[-4, 1]	
Day10 Second time					
Number of participants	36	36	36	33	p=0.738
Mean (standard deviation)	0.3 (0.7)	-0.9 (1.2)	0.2 (0.5)	-0.8 (1.3)	
Median	0.0	0.0	0.0	0.0	
[Minimum, Maximum]	[0, 3]	[-4, 1]	[0, 2]	[-4, 1]	

Shortness of breath

Time	EFE group (N=41)		Placebo group (N=40)		Wilcoxon rank sum test
	Measurement	Change	Measurement	Change	
Day11 First time					
Number of participants	39	39	35	33	p=0.191
Mean (standard deviation)	0.4 (0.8)	-0.8 (1.3)	0.1 (0.4)	-0.9 (1.2)	
Median	0.0	0.0	0.0	0.0	
[Minimum, Maximum]	[0, 3]	[-5, 1]	[0, 1]	[-4, 1]	
Day11 Second time					
Number of participants	34	34	34	32	p=0.278
Mean (standard deviation)	0.4 (0.8)	-0.7 (1.1)	0.1 (0.4)	-0.9 (1.3)	
Median	0.0	0.0	0.0	0.0	
[Minimum, Maximum]	[0, 3]	[-4, 1]	[0, 1]	[-4, 1]	
Day12 First time					
Number of participants	38	38	35	33	p=0.778
Mean (standard deviation)	0.3 (0.8)	-0.9 (1.3)	0.2 (0.4)	-0.8 (1.3)	
Median	0.0	0.0	0.0	0.0	
[Minimum, Maximum]	[0, 4]	[-4, 1]	[0, 1]	[-4, 1]	
Day12 Second time					
Number of participants	34	34	33	31	p=0.755
Mean (standard deviation)	0.3 (0.7)	-0.9 (1.3)	0.2 (0.5)	-0.9 (1.4)	
Median	0.0	0.0	0.0	0.0	
[Minimum, Maximum]	[0, 3]	[-4, 1]	[0, 2]	[-4, 1]	

Shortness of breath

Time	EFE group (N=41)		Placebo group (N=40)		Wilcoxon rank sum test
	Measurement	Change	Measurement	Change	
Day13 First time					
Number of participants	38	38	36	33	p=0.494
Mean (standard deviation)	0.3 (0.8)	-0.9 (1.3)	0.1 (0.4)	-0.9 (1.3)	
Median	0.0	0.0	0.0	0.0	
[Minimum, Maximum]	[0, 4]	[-4, 1]	[0, 1]	[-4, 1]	
Day13 Second time					
Number of participants	35	35	33	31	p=0.347
Mean (standard deviation)	0.3 (0.8)	-1.0 (1.3)	0.1 (0.3)	-0.9 (1.3)	
Median	0.0	0.0	0.0	0.0	
[Minimum, Maximum]	[0, 4]	[-4, 1]	[0, 1]	[-4, 1]	
Day14 First time					
Number of participants	39	39	34	32	p=0.332
Mean (standard deviation)	0.3 (0.7)	-0.9 (1.3)	0.1 (0.4)	-0.8 (1.3)	
Median	0.0	0.0	0.0	0.0	
[Minimum, Maximum]	[0, 3]	[-4, 1]	[0, 1]	[-4, 1]	
Day14 Second time					
Number of participants	35	35	34	32	p=0.382
Mean (standard deviation)	0.3 (0.8)	-0.7 (1.3)	0.2 (0.5)	-0.8 (1.4)	
Median	0.0	0.0	0.0	0.0	
[Minimum, Maximum]	[0, 4]	[-5, 1]	[0, 2]	[-4, 1]	

Shortness of breath

Time	EFE group (N=41)		Placebo group (N=40)		Wilcoxon rank sum test
	Measurement	Change	Measurement	Change	
Day15 First time					
Number of participants	37	37	34	31	p=0.477
Mean (standard deviation)	0.2 (0.5)	-0.9 (1.3)	0.2 (0.5)	-0.7 (1.3)	
Median	0.0	0.0	0.0	0.0	
[Minimum, Maximum]	[0, 2]	[-4, 1]	[0, 2]	[-4, 1]	

Cough

Time	EFE group (N=41)		Placebo group (N=40)		Wilcoxon rank sum test
	Measurement	Change	Measurement	Change	
Day1 First time					
Number of participants	31		31		
Mean (standard deviation)	3.0 (1.5)		2.8 (1.4)		
Median	3.0		3.0		
[Minimum, Maximum]	[0, 5]		[0, 5]		
Day1 Second time					
Number of participants	27	27	24	24	p=0.524
Mean (standard deviation)	2.6 (1.6)	-0.3 (1.0)	2.8 (1.6)	-0.1 (1.0)	
Median	3.0	0.0	3.0	0.0	
[Minimum, Maximum]	[0, 6]	[-2, 3]	[0, 6]	[-2, 3]	
Day2 First time					
Number of participants	25	25	27	25	p=0.532
Mean (standard deviation)	2.6 (1.6)	-0.3 (1.4)	2.3 (1.5)	-0.4 (1.3)	
Median	3.0	0.0	3.0	0.0	
[Minimum, Maximum]	[0, 5]	[-3, 4]	[0, 5]	[-3, 3]	
Day2 Second time					
Number of participants	24	24	27	25	p=0.391
Mean (standard deviation)	1.9 (1.6)	-0.9 (1.3)	2.2 (1.4)	-0.5 (1.4)	
Median	2.0	-1.0	2.0	0.0	
[Minimum, Maximum]	[0, 6]	[-4, 2]	[0, 5]	[-3, 3]	

Cough

Time	EFE group (N=41)		Placebo group (N=40)		Wilcoxon rank sum test
	Measurement	Change	Measurement	Change	
Day3 First time					
Number of participants	21	21	27	25	p=0.881
Mean (standard deviation)	2.1 (1.6)	-0.8 (1.7)	2.0 (1.3)	-0.7 (1.5)	
Median	2.0	-1.0	2.0	-1.0	
[Minimum, Maximum]	[0, 6]	[-4, 3]	[0, 5]	[-4, 3]	
Day3 Second time					
Number of participants	20	20	25	24	p=0.572
Mean (standard deviation)	1.7 (1.3)	-1.1 (1.4)	1.8 (1.3)	-1.0 (1.7)	
Median	1.0	-1.0	2.0	-1.0	
[Minimum, Maximum]	[0, 5]	[-3, 2]	[0, 4]	[-4, 3]	
Day4 First time					
Number of participants	20	20	28	25	p=0.437
Mean (standard deviation)	1.6 (1.2)	-1.2 (1.5)	1.9 (1.3)	-1.0 (1.6)	
Median	1.0	-1.0	1.5	-1.0	
[Minimum, Maximum]	[0, 4]	[-4, 2]	[0, 5]	[-4, 3]	
Day4 Second time					
Number of participants	20	20	27	24	p=0.622
Mean (standard deviation)	1.5 (1.1)	-1.3 (1.6)	1.7 (1.3)	-1.1 (1.6)	
Median	1.0	-1.5	2.0	-1.0	
[Minimum, Maximum]	[0, 4]	[-4, 2]	[0, 6]	[-4, 2]	

Cough

Time	EFE group (N=41)		Placebo group (N=40)		Wilcoxon rank sum test
	Measurement	Change	Measurement	Change	
Day5 First time					
Number of participants	20	20	26	23	p=0.213
Mean (standard deviation)	1.3 (1.2)	-1.5 (1.5)	1.8 (1.4)	-1.1 (1.7)	
Median	1.0	-1.0	1.5	-1.0	
[Minimum, Maximum]	[0, 4]	[-4, 1]	[0, 6]	[-4, 2]	
Day5 Second time					
Number of participants	19	19	24	21	p=0.502
Mean (standard deviation)	1.4 (1.2)	-1.5 (1.4)	1.7 (1.4)	-1.2 (1.5)	
Median	1.0	-2.0	1.0	-1.0	
[Minimum, Maximum]	[0, 4]	[-4, 1]	[0, 6]	[-4, 2]	
Day6 First time					
Number of participants	20	20	26	23	p=0.090
Mean (standard deviation)	1.0 (1.0)	-1.8 (1.4)	1.5 (1.3)	-1.4 (1.7)	
Median	1.0	-2.0	1.0	-1.0	
[Minimum, Maximum]	[0, 4]	[-4, 1]	[0, 6]	[-4, 2]	
Day6 Second time					
Number of participants	19	19	22	20	p=0.485
Mean (standard deviation)	1.0 (1.2)	-1.9 (1.7)	1.2 (1.1)	-1.7 (1.5)	
Median	1.0	-2.0	1.0	-2.0	
[Minimum, Maximum]	[0, 3]	[-5, 2]	[0, 4]	[-5, 2]	

Cough

Time	EFE group (N=41)		Placebo group (N=40)		Wilcoxon rank sum test
	Measurement	Change	Measurement	Change	
Day7 First time					
Number of participants	20	20	25	22	p=0.452
Mean (standard deviation)	1.0 (1.1)	-1.8 (1.5)	1.3 (1.4)	-1.5 (1.8)	
Median	1.0	-2.0	1.0	-2.0	
[Minimum, Maximum]	[0, 4]	[-4, 1]	[0, 6]	[-5, 2]	
Day7 Second time					
Number of participants	20	20	23	20	p=0.246
Mean (standard deviation)	0.9 (1.1)	-1.9 (1.5)	1.2 (1.0)	-1.7 (1.5)	
Median	1.0	-2.0	1.0	-1.5	
[Minimum, Maximum]	[0, 4]	[-4, 1]	[0, 3]	[-5, 1]	
Day8 First time					
Number of participants	20	20	22	19	p=0.267
Mean (standard deviation)	0.7 (0.9)	-2.1 (1.4)	1.0 (0.8)	-1.8 (1.5)	
Median	0.5	-2.0	1.0	-2.0	
[Minimum, Maximum]	[0, 3]	[-4, 1]	[0, 3]	[-5, 1]	
Day8 Second time					
Number of participants	19	19	21	19	p=0.129
Mean (standard deviation)	0.9 (1.1)	-1.9 (1.4)	1.2 (0.8)	-1.6 (1.5)	
Median	1.0	-2.0	1.0	-1.0	
[Minimum, Maximum]	[0, 4]	[-4, 1]	[0, 3]	[-5, 1]	

Cough

Time	EFE group (N=41)		Placebo group (N=40)		Wilcoxon rank sum test
	Measurement	Change	Measurement	Change	
Day9 First time					
Number of participants	20	20	23	21	p=0.241
Mean (standard deviation)	0.9 (1.2)	-1.9 (1.4)	1.0 (0.9)	-1.8 (1.3)	
Median	0.5	-2.0	1.0	-2.0	
[Minimum, Maximum]	[0, 4]	[-4, 1]	[0, 3]	[-5, 0]	
Day9 Second time					
Number of participants	19	19	23	21	p=0.272
Mean (standard deviation)	0.7 (1.1)	-2.2 (1.4)	1.0 (1.0)	-1.8 (1.4)	
Median	0.0	-2.0	1.0	-2.0	
[Minimum, Maximum]	[0, 4]	[-4, 1]	[0, 3]	[-5, 1]	
Day10 First time					
Number of participants	19	19	24	21	p=0.082
Mean (standard deviation)	0.7 (1.2)	-1.9 (1.4)	1.0 (0.9)	-1.7 (1.4)	
Median	0.0	-2.0	1.0	-1.0	
[Minimum, Maximum]	[0, 4]	[-4, 0]	[0, 3]	[-5, 0]	
Day10 Second time					
Number of participants	19	19	23	20	p=0.657
Mean (standard deviation)	0.9 (1.0)	-1.8 (1.5)	0.9 (0.7)	-1.9 (1.3)	
Median	1.0	-2.0	1.0	-2.0	
[Minimum, Maximum]	[0, 3]	[-4, 1]	[0, 2]	[-5, 0]	

Cough

Time	EFE group (N=41)		Placebo group (N=40)		Wilcoxon rank sum test
	Measurement	Change	Measurement	Change	
Day11 First time					
Number of participants	20	20	23	21	p=0.460
Mean (standard deviation)	0.8 (1.1)	-2.0 (1.6)	0.8 (0.7)	-1.9 (1.3)	
Median	0.5	-2.0	1.0	-2.0	
[Minimum, Maximum]	[0, 4]	[-4, 1]	[0, 2]	[-5, 0]	
Day11 Second time					
Number of participants	17	17	22	20	p=0.631
Mean (standard deviation)	0.9 (1.2)	-1.8 (1.4)	1.0 (0.8)	-1.8 (1.3)	
Median	1.0	-2.0	1.0	-1.5	
[Minimum, Maximum]	[0, 4]	[-4, 0]	[0, 2]	[-5, 0]	
Day12 First time					
Number of participants	19	19	23	21	p=0.268
Mean (standard deviation)	0.8 (1.2)	-1.9 (1.6)	1.0 (0.8)	-1.8 (1.3)	
Median	0.0	-2.0	1.0	-2.0	
[Minimum, Maximum]	[0, 4]	[-4, 0]	[0, 2]	[-5, 1]	
Day12 Second time					
Number of participants	17	17	22	20	p=0.988
Mean (standard deviation)	0.9 (1.0)	-2.0 (1.5)	0.9 (0.9)	-2.0 (1.3)	
Median	1.0	-3.0	1.0	-2.0	
[Minimum, Maximum]	[0, 4]	[-4, 1]	[0, 3]	[-5, 0]	

Cough

Time	EFE group (N=41)		Placebo group (N=40)		Wilcoxon rank sum test
	Measurement	Change	Measurement	Change	
Day13 First time					
Number of participants	19	19	24	21	p=0.521
Mean (standard deviation)	0.8 (1.1)	-1.8 (1.4)	0.9 (0.9)	-1.8 (1.3)	
Median	1.0	-2.0	1.0	-2.0	
[Minimum, Maximum]	[0, 4]	[-4, 1]	[0, 3]	[-5, 0]	
Day13 Second time					
Number of participants	18	18	21	19	p=0.517
Mean (standard deviation)	0.7 (1.0)	-2.1 (1.4)	0.8 (0.8)	-2.1 (1.3)	
Median	0.0	-2.5	1.0	-2.0	
[Minimum, Maximum]	[0, 4]	[-4, 0]	[0, 2]	[-5, 0]	
Day14 First time					
Number of participants	20	20	22	20	p=0.474
Mean (standard deviation)	0.7 (1.1)	-2.1 (1.5)	0.8 (0.8)	-1.9 (1.3)	
Median	0.0	-2.0	1.0	-2.0	
[Minimum, Maximum]	[0, 4]	[-4, 1]	[0, 2]	[-5, 0]	
Day14 Second time					
Number of participants	18	18	22	20	p=0.778
Mean (standard deviation)	0.8 (1.1)	-1.9 (1.6)	0.6 (0.7)	-2.1 (1.3)	
Median	0.5	-2.0	0.5	-2.0	
[Minimum, Maximum]	[0, 4]	[-4, 2]	[0, 2]	[-5, 0]	

Cough

Time	EFE group (N=41)		Placebo group (N=40)		Wilcoxon rank sum test
	Measurement	Change	Measurement	Change	
Day15 First time					
Number of participants	18	18	23	20	p=1.000
Mean (standard deviation)	0.6 (0.6)	-2.2 (1.4)	0.7 (0.9)	-2.0 (1.5)	
Median	0.5	-3.0	0.0	-2.0	
[Minimum, Maximum]	[0, 2]	[-4, 1]	[0, 3]	[-5, 1]	

Nasal discharge

Time	EFE group (N=41)		Placebo group (N=40)		Wilcoxon rank sum test
	Measurement	Change	Measurement	Change	
Day1 First time					
Number of participants	41		37		
Mean (standard deviation)	2.6 (1.5)		2.3 (1.5)		
Median	3.0		2.0		
[Minimum, Maximum]	[0, 6]		[0, 6]		
Day1 Second time					
Number of participants	41	41	35	35	p=0.256
Mean (standard deviation)	2.5 (1.6)	-0.1 (1.2)	2.1 (1.4)	-0.3 (0.9)	
Median	2.0	0.0	2.0	0.0	
[Minimum, Maximum]	[0, 6]	[-2, 4]	[0, 6]	[-2, 2]	
Day2 First time					
Number of participants	41	41	38	36	p=0.688
Mean (standard deviation)	2.4 (1.5)	-0.2 (1.6)	2.2 (1.4)	-0.2 (1.5)	
Median	2.0	0.0	2.0	0.0	
[Minimum, Maximum]	[0, 6]	[-3, 4]	[0, 5]	[-3, 3]	
Day2 Second time					
Number of participants	39	39	37	35	p=0.623
Mean (standard deviation)	2.2 (1.5)	-0.4 (1.5)	2.0 (1.5)	-0.4 (1.7)	
Median	2.0	-1.0	2.0	0.0	
[Minimum, Maximum]	[0, 6]	[-3, 3]	[0, 6]	[-3, 5]	

Nasal discharge

Time	EFE group (N=41)		Placebo group (N=40)		Wilcoxon rank sum test
	Measurement	Change	Measurement	Change	
Day3 First time					
Number of participants	40	40	38	36	p=0.430
Mean (standard deviation)	2.1 (1.5)	-0.4 (1.8)	1.9 (1.3)	-0.5 (1.7)	
Median	2.0	-1.0	1.5	-1.0	
[Minimum, Maximum]	[0, 6]	[-3, 3]	[0, 5]	[-3, 3]	
Day3 Second time					
Number of participants	39	39	36	35	p=0.448
Mean (standard deviation)	1.8 (1.4)	-0.7 (1.8)	1.6 (1.1)	-0.9 (1.5)	
Median	2.0	-1.0	1.0	-1.0	
[Minimum, Maximum]	[0, 5]	[-3, 4]	[0, 4]	[-4, 3]	
Day4 First time					
Number of participants	40	40	39	36	p=0.826
Mean (standard deviation)	1.6 (1.3)	-1.0 (1.9)	1.4 (1.0)	-1.0 (1.6)	
Median	1.0	-1.0	2.0	-1.0	
[Minimum, Maximum]	[0, 5]	[-4, 4]	[0, 3]	[-4, 2]	
Day4 Second time					
Number of participants	39	39	39	36	p=0.183
Mean (standard deviation)	1.7 (1.4)	-0.9 (1.9)	1.3 (1.1)	-1.2 (1.8)	
Median	2.0	-1.0	1.0	-1.0	
[Minimum, Maximum]	[0, 6]	[-4, 3]	[0, 5]	[-5, 4]	

Nasal discharge

Time	EFE group (N=41)		Placebo group (N=40)		Wilcoxon rank sum test
	Measurement	Change	Measurement	Change	
Day5 First time					
Number of participants	40	40	39	36	p=0.246
Mean (standard deviation)	1.6 (1.4)	-1.0 (1.9)	1.2 (0.9)	-1.2 (1.6)	
Median	1.5	-1.0	1.0	-1.0	
[Minimum, Maximum]	[0, 6]	[-4, 3]	[0, 4]	[-4, 3]	
Day5 Second time					
Number of participants	39	39	36	33	p=0.106
Mean (standard deviation)	1.4 (1.2)	-1.1 (1.8)	1.0 (0.9)	-1.5 (1.6)	
Median	1.0	-1.0	1.0	-1.0	
[Minimum, Maximum]	[0, 6]	[-4, 3]	[0, 3]	[-5, 1]	
Day6 First time					
Number of participants	40	40	39	36	p=0.712
Mean (standard deviation)	1.2 (1.2)	-1.3 (1.7)	1.1 (1.0)	-1.4 (1.6)	
Median	1.0	-1.0	1.0	-1.0	
[Minimum, Maximum]	[0, 6]	[-4, 3]	[0, 4]	[-6, 1]	
Day6 Second time					
Number of participants	38	38	36	34	p=0.258
Mean (standard deviation)	1.3 (1.2)	-1.2 (1.8)	1.0 (1.0)	-1.4 (1.4)	
Median	1.0	-1.0	1.0	-1.0	
[Minimum, Maximum]	[0, 6]	[-4, 4]	[0, 4]	[-5, 1]	

Nasal discharge

Time	EFE group (N=41)		Placebo group (N=40)		Wilcoxon rank sum test
	Measurement	Change	Measurement	Change	
Day7 First time					
Number of participants	40	40	39	36	p=0.561
Mean (standard deviation)	1.3 (1.1)	-1.3 (1.7)	1.1 (1.0)	-1.3 (1.5)	
Median	1.0	-1.5	1.0	-1.0	
[Minimum, Maximum]	[0, 5]	[-4, 3]	[0, 3]	[-5, 2]	
Day7 Second time					
Number of participants	39	39	36	33	p=0.854
Mean (standard deviation)	1.1 (1.2)	-1.4 (1.7)	1.1 (1.0)	-1.5 (1.6)	
Median	1.0	-2.0	1.0	-1.0	
[Minimum, Maximum]	[0, 6]	[-4, 3]	[0, 3]	[-6, 1]	
Day8 First time					
Number of participants	39	39	36	33	p=0.353
Mean (standard deviation)	1.2 (1.1)	-1.4 (1.9)	0.9 (0.8)	-1.6 (1.5)	
Median	1.0	-2.0	1.0	-2.0	
[Minimum, Maximum]	[0, 5]	[-4, 3]	[0, 3]	[-6, 1]	
Day8 Second time					
Number of participants	37	37	35	33	p=0.558
Mean (standard deviation)	1.1 (1.1)	-1.4 (1.8)	1.0 (1.0)	-1.4 (1.3)	
Median	1.0	-2.0	1.0	-2.0	
[Minimum, Maximum]	[0, 5]	[-4, 3]	[0, 3]	[-4, 2]	

Nasal discharge

Time	EFE group (N=41)		Placebo group (N=40)		Wilcoxon rank sum test
	Measurement	Change	Measurement	Change	
Day9 First time					
Number of participants	39	39	37	35	p=0.499
Mean (standard deviation)	1.1 (1.0)	-1.5 (1.7)	0.9 (0.9)	-1.5 (1.3)	
Median	1.0	-2.0	1.0	-1.0	
[Minimum, Maximum]	[0, 4]	[-4, 2]	[0, 3]	[-5, 1]	
Day9 Second time					
Number of participants	37	37	37	35	p=0.363
Mean (standard deviation)	1.0 (1.0)	-1.6 (1.7)	0.9 (1.0)	-1.5 (1.5)	
Median	1.0	-2.0	1.0	-2.0	
[Minimum, Maximum]	[0, 4]	[-4, 2]	[0, 4]	[-6, 1]	
Day10 First time					
Number of participants	38	38	38	35	p=0.246
Mean (standard deviation)	1.1 (1.1)	-1.5 (1.9)	0.8 (0.9)	-1.7 (1.5)	
Median	1.0	-2.0	1.0	-2.0	
[Minimum, Maximum]	[0, 5]	[-4, 4]	[0, 3]	[-6, 1]	
Day10 Second time					
Number of participants	36	36	36	33	p=0.046
Mean (standard deviation)	1.1 (1.0)	-1.5 (1.7)	0.6 (0.8)	-1.8 (1.6)	
Median	1.0	-2.0	0.5	-2.0	
[Minimum, Maximum]	[0, 4]	[-4, 2]	[0, 3]	[-6, 1]	

Nasal discharge

Time	EFE group (N=41)		Placebo group (N=40)		Wilcoxon rank sum test
	Measurement	Change	Measurement	Change	
Day11 First time					
Number of participants	39	39	35	33	p=0.015
Mean (standard deviation)	1.1 (1.0)	-1.5 (1.8)	0.6 (0.8)	-1.8 (1.5)	
Median	1.0	-2.0	0.0	-2.0	
[Minimum, Maximum]	[0, 4]	[-4, 4]	[0, 3]	[-6, 1]	
Day11 Second time					
Number of participants	34	34	34	32	p=0.053
Mean (standard deviation)	0.9 (0.8)	-1.6 (1.7)	0.6 (0.7)	-1.8 (1.5)	
Median	1.0	-2.0	0.0	-2.0	
[Minimum, Maximum]	[0, 3]	[-4, 1]	[0, 2]	[-6, 1]	
Day12 First time					
Number of participants	38	38	35	33	p=0.027
Mean (standard deviation)	0.9 (0.9)	-1.6 (1.7)	0.5 (0.7)	-1.9 (1.5)	
Median	1.0	-2.0	0.0	-2.0	
[Minimum, Maximum]	[0, 3]	[-4, 2]	[0, 2]	[-6, 1]	
Day12 Second time					
Number of participants	34	34	33	31	p=0.270
Mean (standard deviation)	0.8 (0.9)	-1.8 (1.9)	0.6 (0.8)	-1.8 (1.4)	
Median	1.0	-2.0	0.0	-2.0	
[Minimum, Maximum]	[0, 3]	[-4, 3]	[0, 3]	[-5, 1]	

Nasal discharge

Time	EFE group (N=41)		Placebo group (N=40)		Wilcoxon rank sum test
	Measurement	Change	Measurement	Change	
Day13 First time					
Number of participants	38	38	36	33	p=0.244
Mean (standard deviation)	0.8 (0.9)	-1.7 (1.7)	0.6 (0.8)	-1.8 (1.3)	
Median	1.0	-2.0	0.0	-2.0	
[Minimum, Maximum]	[0, 3]	[-4, 3]	[0, 3]	[-5, 1]	
Day13 Second time					
Number of participants	35	35	33	31	p=0.419
Mean (standard deviation)	0.7 (0.9)	-1.8 (1.8)	0.5 (0.7)	-1.9 (1.4)	
Median	1.0	-2.0	0.0	-2.0	
[Minimum, Maximum]	[0, 4]	[-4, 2]	[0, 2]	[-5, 1]	
Day14 First time					
Number of participants	39	39	34	32	p=0.334
Mean (standard deviation)	0.7 (1.0)	-1.8 (1.8)	0.5 (0.7)	-1.9 (1.5)	
Median	0.0	-2.0	0.0	-2.0	
[Minimum, Maximum]	[0, 4]	[-5, 4]	[0, 2]	[-6, 0]	
Day14 Second time					
Number of participants	35	35	34	32	p=0.113
Mean (standard deviation)	0.7 (1.0)	-1.8 (1.9)	0.4 (0.7)	-2.1 (1.5)	
Median	1.0	-2.0	0.0	-2.0	
[Minimum, Maximum]	[0, 4]	[-5, 3]	[0, 2]	[-6, 0]	

Nasal discharge

Time	EFE group (N=41)		Placebo group (N=40)		Wilcoxon rank sum test
	Measurement	Change	Measurement	Change	
Day15 First time					
Number of participants	37	37	34	31	p=0.687
Mean (standard deviation)	0.5 (0.6)	-2.0 (1.6)	0.4 (0.7)	-2.0 (1.4)	
Median	0.0	-2.0	0.0	-2.0	
[Minimum, Maximum]	[0, 2]	[-4, 2]	[0, 2]	[-6, 0]	

Nausea

Time	EFE group (N=41)		Placebo group (N=40)		Wilcoxon rank sum test
	Measurement	Change	Measurement	Change	
Day1 First time					
Number of participants	41		37		
Mean (standard deviation)	0.3 (0.9)		0.4 (1.0)		
Median	0.0		0.0		
[Minimum, Maximum]	[0, 4]		[0, 4]		
Day1 Second time					
Number of participants	41	41	35	35	p=0.603
Mean (standard deviation)	0.3 (0.6)	0.0 (0.9)	0.4 (0.9)	0.0 (0.7)	
Median	0.0	0.0	0.0	0.0	
[Minimum, Maximum]	[0, 2]	[-3, 2]	[0, 4]	[-2, 3]	
Day2 First time					
Number of participants	41	41	38	36	p=0.791
Mean (standard deviation)	0.2 (0.6)	-0.1 (0.6)	0.4 (1.0)	-0.1 (0.6)	
Median	0.0	0.0	0.0	0.0	
[Minimum, Maximum]	[0, 2]	[-2, 1]	[0, 4]	[-2, 2]	
Day2 Second time					
Number of participants	39	39	37	35	p=0.575
Mean (standard deviation)	0.2 (0.6)	-0.1 (0.9)	0.4 (1.0)	-0.1 (0.6)	
Median	0.0	0.0	0.0	0.0	
[Minimum, Maximum]	[0, 3]	[-3, 3]	[0, 3]	[-2, 2]	

Nausea

Time	EFE group (N=41)		Placebo group (N=40)		Wilcoxon rank sum test
	Measurement	Change	Measurement	Change	
Day3 First time					
Number of participants	40	40	38	36	p=0.063
Mean (standard deviation)	0.2 (0.5)	-0.2 (0.9)	0.5 (1.1)	0.1 (0.9)	
Median	0.0	0.0	0.0	0.0	
[Minimum, Maximum]	[0, 2]	[-4, 1]	[0, 5]	[-2, 4]	
Day3 Second time					
Number of participants	39	39	36	35	p=0.148
Mean (standard deviation)	0.1 (0.5)	-0.2 (0.8)	0.3 (0.7)	-0.1 (1.0)	
Median	0.0	0.0	0.0	0.0	
[Minimum, Maximum]	[0, 2]	[-4, 1]	[0, 3]	[-3, 3]	
Day4 First time					
Number of participants	40	40	39	36	p=0.154
Mean (standard deviation)	0.1 (0.4)	-0.3 (0.9)	0.3 (0.8)	-0.1 (0.8)	
Median	0.0	0.0	0.0	0.0	
[Minimum, Maximum]	[0, 2]	[-4, 1]	[0, 4]	[-2, 3]	
Day4 Second time					
Number of participants	39	39	39	36	p=0.199
Mean (standard deviation)	0.1 (0.4)	-0.2 (0.8)	0.3 (0.8)	-0.1 (0.9)	
Median	0.0	0.0	0.0	0.0	
[Minimum, Maximum]	[0, 2]	[-4, 1]	[0, 3]	[-3, 2]	

Nausea

Time	EFE group (N=41)		Placebo group (N=40)		Wilcoxon rank sum test
	Measurement	Change	Measurement	Change	
Day5 First time					
Number of participants	40	40	39	36	p=0.043
Mean (standard deviation)	0.1 (0.3)	-0.3 (0.9)	0.3 (0.8)	-0.1 (1.0)	
Median	0.0	0.0	0.0	0.0	
[Minimum, Maximum]	[0, 2]	[-4, 1]	[0, 4]	[-3, 3]	
Day5 Second time					
Number of participants	39	39	36	33	p=0.225
Mean (standard deviation)	0.1 (0.4)	-0.3 (0.9)	0.3 (0.7)	-0.1 (1.1)	
Median	0.0	0.0	0.0	0.0	
[Minimum, Maximum]	[0, 2]	[-4, 1]	[0, 3]	[-4, 2]	
Day6 First time					
Number of participants	40	40	39	36	p=0.166
Mean (standard deviation)	0.1 (0.4)	-0.3 (0.9)	0.3 (0.9)	-0.1 (1.3)	
Median	0.0	0.0	0.0	0.0	
[Minimum, Maximum]	[0, 2]	[-4, 1]	[0, 4]	[-4, 4]	
Day6 Second time					
Number of participants	38	38	36	34	p=0.391
Mean (standard deviation)	0.1 (0.4)	-0.3 (0.9)	0.1 (0.3)	-0.3 (0.9)	
Median	0.0	0.0	0.0	0.0	
[Minimum, Maximum]	[0, 2]	[-4, 1]	[0, 1]	[-4, 1]	

Nausea

Time	EFE group (N=41)		Placebo group (N=40)		Wilcoxon rank sum test
	Measurement	Change	Measurement	Change	
Day7 First time					
Number of participants	40	40	39	36	p=0.448
Mean (standard deviation)	0.1 (0.4)	-0.3 (0.9)	0.2 (0.6)	-0.2 (1.0)	
Median	0.0	0.0	0.0	0.0	
[Minimum, Maximum]	[0, 2]	[-4, 1]	[0, 3]	[-4, 2]	
Day7 Second time					
Number of participants	39	39	36	33	p=0.373
Mean (standard deviation)	0.1 (0.4)	-0.3 (0.9)	0.1 (0.3)	-0.3 (0.9)	
Median	0.0	0.0	0.0	0.0	
[Minimum, Maximum]	[0, 2]	[-4, 1]	[0, 1]	[-4, 1]	
Day8 First time					
Number of participants	39	39	36	33	p=0.542
Mean (standard deviation)	0.1 (0.3)	-0.3 (0.9)	0.1 (0.2)	-0.3 (0.9)	
Median	0.0	0.0	0.0	0.0	
[Minimum, Maximum]	[0, 2]	[-4, 0]	[0, 1]	[-4, 0]	
Day8 Second time					
Number of participants	37	37	35	33	p=0.307
Mean (standard deviation)	0.1 (0.3)	-0.3 (0.9)	0.1 (0.3)	-0.3 (1.0)	
Median	0.0	0.0	0.0	0.0	
[Minimum, Maximum]	[0, 2]	[-4, 0]	[0, 1]	[-4, 1]	

Nausea

Time	EFE group (N=41)		Placebo group (N=40)		Wilcoxon rank sum test
	Measurement	Change	Measurement	Change	
Day9 First time					
Number of participants	39	39	37	35	p=0.327
Mean (standard deviation)	0.2 (0.6)	-0.2 (1.1)	0.0 (0.2)	-0.3 (0.9)	
Median	0.0	0.0	0.0	0.0	
[Minimum, Maximum]	[0, 3]	[-4, 3]	[0, 1]	[-4, 0]	
Day9 Second time					
Number of participants	37	37	37	35	p=0.591
Mean (standard deviation)	0.1 (0.3)	-0.3 (0.9)	0.1 (0.2)	-0.3 (0.9)	
Median	0.0	0.0	0.0	0.0	
[Minimum, Maximum]	[0, 2]	[-4, 0]	[0, 1]	[-4, 0]	
Day10 First time					
Number of participants	38	38	38	35	p=0.334
Mean (standard deviation)	0.1 (0.3)	-0.3 (0.9)	0.1 (0.3)	-0.3 (0.9)	
Median	0.0	0.0	0.0	0.0	
[Minimum, Maximum]	[0, 2]	[-4, 0]	[0, 1]	[-4, 1]	
Day10 Second time					
Number of participants	36	36	36	33	p=1.000
Mean (standard deviation)	0.1 (0.3)	-0.3 (0.9)	0.0 (0.2)	-0.4 (0.9)	
Median	0.0	0.0	0.0	0.0	
[Minimum, Maximum]	[0, 2]	[-4, 0]	[0, 1]	[-4, 0]	

Nausea

Time	EFE group (N=41)		Placebo group (N=40)		Wilcoxon rank sum test
	Measurement	Change	Measurement	Change	
Day11 First time					
Number of participants	39	39	35	33	p=0.969
Mean (standard deviation)	0.1 (0.3)	-0.3 (0.9)	0.0 (0.2)	-0.3 (0.9)	
Median	0.0	0.0	0.0	0.0	
[Minimum, Maximum]	[0, 2]	[-4, 0]	[0, 1]	[-4, 0]	
Day11 Second time					
Number of participants	34	34	34	32	p=0.332
Mean (standard deviation)	0.0 (0.0)	-0.4 (0.9)	0.0 (0.2)	-0.3 (0.9)	
Median	0.0	0.0	0.0	0.0	
[Minimum, Maximum]	[0, 0]	[-4, 0]	[0, 1]	[-4, 0]	
Day12 First time					
Number of participants	38	38	35	33	p=0.984
Mean (standard deviation)	0.1 (0.3)	-0.3 (0.9)	0.0 (0.2)	-0.3 (0.9)	
Median	0.0	0.0	0.0	0.0	
[Minimum, Maximum]	[0, 2]	[-4, 0]	[0, 1]	[-4, 0]	
Day12 Second time					
Number of participants	34	34	33	31	p=1.000
Mean (standard deviation)	0.1 (0.3)	-0.3 (0.9)	0.0 (0.2)	-0.4 (1.0)	
Median	0.0	0.0	0.0	0.0	
[Minimum, Maximum]	[0, 2]	[-4, 0]	[0, 1]	[-4, 0]	

Nausea

Time	EFE group (N=41)		Placebo group (N=40)		Wilcoxon rank sum test
	Measurement	Change	Measurement	Change	
Day13 First time					
Number of participants	38	38	36	33	p=0.558
Mean (standard deviation)	0.1 (0.3)	-0.3 (0.9)	0.1 (0.2)	-0.3 (1.0)	
Median	0.0	0.0	0.0	0.0	
[Minimum, Maximum]	[0, 2]	[-4, 0]	[0, 1]	[-4, 1]	
Day13 Second time					
Number of participants	35	35	33	31	p=0.346
Mean (standard deviation)	0.0 (0.2)	-0.4 (1.0)	0.0 (0.0)	-0.3 (0.8)	
Median	0.0	0.0	0.0	0.0	
[Minimum, Maximum]	[0, 1]	[-4, 1]	[0, 0]	[-4, 0]	
Day14 First time					
Number of participants	39	39	34	32	p=0.297
Mean (standard deviation)	0.0 (0.0)	-0.4 (0.9)	0.0 (0.2)	-0.3 (0.9)	
Median	0.0	0.0	0.0	0.0	
[Minimum, Maximum]	[0, 0]	[-4, 0]	[0, 1]	[-4, 0]	
Day14 Second time					
Number of participants	35	35	34	32	p=0.324
Mean (standard deviation)	0.0 (0.0)	-0.3 (0.9)	0.0 (0.2)	-0.3 (0.9)	
Median	0.0	0.0	0.0	0.0	
[Minimum, Maximum]	[0, 0]	[-4, 0]	[0, 1]	[-4, 0]	

Nausea

Time	EFE group (N=41)		Placebo group (N=40)		Wilcoxon rank sum test
	Measurement	Change	Measurement	Change	
Day15 First time					
Number of participants	37	37	34	31	p=0.310
Mean (standard deviation)	0.0 (0.0)	-0.3 (0.7)	0.0 (0.2)	-0.3 (0.9)	
Median	0.0	0.0	0.0	0.0	
[Minimum, Maximum]	[0, 0]	[-3, 0]	[0, 1]	[-4, 0]	

Loss of appetite

Time	EFE group (N=41)		Placebo group (N=40)		Wilcoxon rank sum test
	Measurement	Change	Measurement	Change	
Day1 First time					
Number of participants	41		37		
Mean (standard deviation)	1.0 (1.1)		1.7 (1.4)		
Median	1.0		2.0		
[Minimum, Maximum]	[0, 4]		[0, 5]		
Day1 Second time					
Number of participants	41	41	35	35	p=0.003
Mean (standard deviation)	0.5 (0.9)	-0.5 (1.0)	1.2 (1.1)	-0.5 (1.0)	
Median	0.0	0.0	1.0	-1.0	
[Minimum, Maximum]	[0, 3]	[-3, 2]	[0, 4]	[-2, 2]	
Day2 First time					
Number of participants	41	41	38	36	p=0.002
Mean (standard deviation)	0.5 (0.7)	-0.5 (0.9)	1.3 (1.3)	-0.3 (1.1)	
Median	0.0	0.0	1.0	0.0	
[Minimum, Maximum]	[0, 2]	[-3, 1]	[0, 4]	[-4, 2]	
Day2 Second time					
Number of participants	39	39	37	35	p=0.015
Mean (standard deviation)	0.5 (0.8)	-0.5 (1.1)	1.1 (1.3)	-0.5 (1.2)	
Median	0.0	0.0	1.0	0.0	
[Minimum, Maximum]	[0, 3]	[-3, 1]	[0, 6]	[-2, 4]	

Loss of appetite

Time	EFE group (N=41)		Placebo group (N=40)		Wilcoxon rank sum test
	Measurement	Change	Measurement	Change	
Day3 First time					
Number of participants	40	40	38	36	p=0.003
Mean (standard deviation)	0.4 (0.6)	-0.7 (1.0)	1.2 (1.3)	-0.4 (1.3)	
Median	0.0	0.0	1.0	0.0	
[Minimum, Maximum]	[0, 2]	[-3, 1]	[0, 5]	[-3, 3]	
Day3 Second time					
Number of participants	39	39	36	35	p<0.001
Mean (standard deviation)	0.3 (0.6)	-0.7 (1.1)	0.8 (0.9)	-0.8 (1.2)	
Median	0.0	0.0	1.0	-1.0	
[Minimum, Maximum]	[0, 3]	[-3, 1]	[0, 3]	[-4, 2]	
Day4 First time					
Number of participants	40	40	39	36	p<0.001
Mean (standard deviation)	0.3 (0.6)	-0.8 (1.0)	0.8 (0.9)	-0.9 (1.3)	
Median	0.0	0.0	1.0	-1.0	
[Minimum, Maximum]	[0, 3]	[-3, 0]	[0, 4]	[-5, 2]	
Day4 Second time					
Number of participants	39	39	39	36	p=0.008
Mean (standard deviation)	0.3 (0.7)	-0.7 (1.0)	0.7 (0.9)	-0.9 (1.4)	
Median	0.0	0.0	0.0	-0.5	
[Minimum, Maximum]	[0, 3]	[-3, 1]	[0, 3]	[-5, 1]	

Loss of appetite

Time	EFE group (N=41)		Placebo group (N=40)		Wilcoxon rank sum test
	Measurement	Change	Measurement	Change	
Day5 First time					
Number of participants	40	40	39	36	p=0.162
Mean (standard deviation)	0.3 (0.7)	-0.7 (1.1)	0.6 (0.9)	-1.0 (1.5)	
Median	0.0	0.0	0.0	-1.0	
[Minimum, Maximum]	[0, 3]	[-3, 2]	[0, 3]	[-5, 1]	
Day5 Second time					
Number of participants	39	39	36	33	p=0.051
Mean (standard deviation)	0.2 (0.5)	-0.9 (1.1)	0.6 (0.9)	-1.0 (1.5)	
Median	0.0	-1.0	0.0	-1.0	
[Minimum, Maximum]	[0, 2]	[-3, 1]	[0, 3]	[-5, 1]	
Day6 First time					
Number of participants	40	40	39	36	p=0.052
Mean (standard deviation)	0.3 (0.6)	-0.8 (1.0)	0.6 (1.0)	-1.0 (1.6)	
Median	0.0	-0.5	0.0	-1.0	
[Minimum, Maximum]	[0, 3]	[-3, 1]	[0, 4]	[-5, 2]	
Day6 Second time					
Number of participants	38	38	36	34	p=0.096
Mean (standard deviation)	0.3 (0.6)	-0.8 (1.1)	0.5 (0.8)	-1.1 (1.4)	
Median	0.0	-0.5	0.0	-1.0	
[Minimum, Maximum]	[0, 2]	[-3, 2]	[0, 3]	[-5, 1]	

Loss of appetite

Time	EFE group (N=41)		Placebo group (N=40)		Wilcoxon rank sum test
	Measurement	Change	Measurement	Change	
Day7 First time					
Number of participants	40	40	39	36	p=0.015
Mean (standard deviation)	0.2 (0.6)	-0.9 (1.1)	0.5 (0.7)	-1.1 (1.4)	
Median	0.0	-1.0	0.0	-1.0	
[Minimum, Maximum]	[0, 2]	[-3, 2]	[0, 2]	[-5, 1]	
Day7 Second time					
Number of participants	39	39	36	33	p=0.081
Mean (standard deviation)	0.2 (0.5)	-0.9 (1.1)	0.4 (0.6)	-1.2 (1.4)	
Median	0.0	-1.0	0.0	-1.0	
[Minimum, Maximum]	[0, 2]	[-3, 2]	[0, 2]	[-5, 1]	
Day8 First time					
Number of participants	39	39	36	33	p=0.104
Mean (standard deviation)	0.2 (0.6)	-0.8 (1.2)	0.4 (0.8)	-1.2 (1.4)	
Median	0.0	-1.0	0.0	-1.0	
[Minimum, Maximum]	[0, 3]	[-3, 3]	[0, 3]	[-5, 1]	
Day8 Second time					
Number of participants	37	37	35	33	p=0.071
Mean (standard deviation)	0.2 (0.5)	-0.8 (1.1)	0.4 (0.6)	-1.2 (1.4)	
Median	0.0	-1.0	0.0	-1.0	
[Minimum, Maximum]	[0, 2]	[-3, 2]	[0, 2]	[-5, 1]	

Loss of appetite

Time	EFE group (N=41)		Placebo group (N=40)		Wilcoxon rank sum test
	Measurement	Change	Measurement	Change	
Day9 First time					
Number of participants	39	39	37	35	p=0.868
Mean (standard deviation)	0.3 (0.7)	-0.7 (1.3)	0.3 (0.7)	-1.3 (1.5)	
Median	0.0	-1.0	0.0	-1.0	
[Minimum, Maximum]	[0, 3]	[-3, 3]	[0, 3]	[-5, 1]	
Day9 Second time					
Number of participants	37	37	37	35	p=0.124
Mean (standard deviation)	0.1 (0.5)	-0.9 (1.2)	0.3 (0.7)	-1.3 (1.4)	
Median	0.0	-1.0	0.0	-1.0	
[Minimum, Maximum]	[0, 2]	[-4, 2]	[0, 3]	[-5, 1]	
Day10 First time					
Number of participants	38	38	38	35	p=0.212
Mean (standard deviation)	0.2 (0.6)	-0.9 (1.3)	0.2 (0.5)	-1.4 (1.4)	
Median	0.0	-1.0	0.0	-1.0	
[Minimum, Maximum]	[0, 3]	[-4, 3]	[0, 2]	[-5, 0]	
Day10 Second time					
Number of participants	36	36	36	33	p=0.234
Mean (standard deviation)	0.2 (0.5)	-0.8 (1.3)	0.3 (0.8)	-1.3 (1.6)	
Median	0.0	-0.5	0.0	-1.0	
[Minimum, Maximum]	[0, 2]	[-4, 2]	[0, 4]	[-5, 2]	

Loss of appetite

Time	EFE group (N=41)		Placebo group (N=40)		Wilcoxon rank sum test
	Measurement	Change	Measurement	Change	
Day11 First time					
Number of participants	39	39	35	33	p=0.145
Mean (standard deviation)	0.2 (0.6)	-0.9 (1.3)	0.3 (0.8)	-1.3 (1.6)	
Median	0.0	-1.0	0.0	-1.0	
[Minimum, Maximum]	[0, 3]	[-4, 3]	[0, 4]	[-5, 2]	
Day11 Second time					
Number of participants	34	34	34	32	p=0.145
Mean (standard deviation)	0.1 (0.5)	-0.8 (1.3)	0.3 (0.7)	-1.3 (1.6)	
Median	0.0	-0.5	0.0	-1.0	
[Minimum, Maximum]	[0, 3]	[-4, 3]	[0, 3]	[-5, 1]	
Day12 First time					
Number of participants	38	38	35	33	p=0.160
Mean (standard deviation)	0.2 (0.6)	-0.8 (1.3)	0.3 (0.7)	-1.3 (1.6)	
Median	0.0	-1.0	0.0	-1.0	
[Minimum, Maximum]	[0, 3]	[-4, 3]	[0, 3]	[-5, 1]	
Day12 Second time					
Number of participants	34	34	33	31	p=0.396
Mean (standard deviation)	0.1 (0.4)	-0.9 (1.0)	0.2 (0.4)	-1.5 (1.5)	
Median	0.0	-1.0	0.0	-1.0	
[Minimum, Maximum]	[0, 2]	[-3, 0]	[0, 2]	[-5, 0]	

Loss of appetite

Time	EFE group (N=41)		Placebo group (N=40)		Wilcoxon rank sum test
	Measurement	Change	Measurement	Change	
Day13 First time					
Number of participants	38	38	36	33	p=0.155
Mean (standard deviation)	0.1 (0.4)	-0.9 (1.1)	0.3 (0.6)	-1.3 (1.5)	
Median	0.0	-1.0	0.0	-1.0	
[Minimum, Maximum]	[0, 2]	[-4, 1]	[0, 2]	[-5, 1]	
Day13 Second time					
Number of participants	35	35	33	31	p=0.107
Mean (standard deviation)	0.1 (0.2)	-1.0 (1.2)	0.2 (0.6)	-1.3 (1.5)	
Median	0.0	-1.0	0.0	-1.0	
[Minimum, Maximum]	[0, 1]	[-4, 1]	[0, 2]	[-5, 0]	
Day14 First time					
Number of participants	39	39	34	32	p=0.159
Mean (standard deviation)	0.1 (0.2)	-1.0 (1.2)	0.2 (0.5)	-1.3 (1.5)	
Median	0.0	-1.0	0.0	-1.0	
[Minimum, Maximum]	[0, 1]	[-4, 1]	[0, 2]	[-5, 0]	
Day14 Second time					
Number of participants	35	35	34	32	p=0.357
Mean (standard deviation)	0.1 (0.2)	-0.8 (1.1)	0.2 (0.5)	-1.4 (1.5)	
Median	0.0	0.0	0.0	-1.0	
[Minimum, Maximum]	[0, 1]	[-4, 1]	[0, 2]	[-5, 0]	

Loss of appetite

Time	EFE group (N=41)		Placebo group (N=40)		Wilcoxon rank sum test
	Measurement	Change	Measurement	Change	
Day15 First time					
Number of participants	37	37	34	31	p=0.099
Mean (standard deviation)	0.1 (0.2)	-1.0 (1.2)	0.2 (0.6)	-1.3 (1.5)	
Median	0.0	-1.0	0.0	-1.0	
[Minimum, Maximum]	[0, 1]	[-4, 1]	[0, 2]	[-5, 0]	

Diarrhea

Time	EFE group (N=41)		Placebo group (N=40)		Wilcoxon rank sum test
	Measurement	Change	Measurement	Change	
Day1 First time					
Number of participants	41		37		
Mean (standard deviation)	0.5 (0.9)		0.4 (0.8)		
Median	0.0		0.0		
[Minimum, Maximum]	[0, 3]		[0, 3]		
Day1 Second time					
Number of participants	41	41	35	35	p=0.742
Mean (standard deviation)	0.2 (0.7)	-0.2 (0.6)	0.3 (0.8)	-0.1 (0.7)	
Median	0.0	0.0	0.0	0.0	
[Minimum, Maximum]	[0, 3]	[-2, 1]	[0, 3]	[-2, 2]	
Day2 First time					
Number of participants	41	41	38	36	p=0.046
Mean (standard deviation)	0.1 (0.5)	-0.3 (0.8)	0.6 (1.1)	0.0 (0.9)	
Median	0.0	0.0	0.0	0.0	
[Minimum, Maximum]	[0, 2]	[-3, 1]	[0, 4]	[-2, 3]	
Day2 Second time					
Number of participants	39	39	37	35	p=0.010
Mean (standard deviation)	0.3 (0.8)	-0.2 (1.1)	0.8 (1.4)	0.3 (1.5)	
Median	0.0	0.0	0.0	0.0	
[Minimum, Maximum]	[0, 3]	[-3, 3]	[0, 6]	[-2, 6]	

Diarrhea

Time	EFE group (N=41)		Placebo group (N=40)		Wilcoxon rank sum test
	Measurement	Change	Measurement	Change	
Day3 First time					
Number of participants	40	40	38	36	p=0.102
Mean (standard deviation)	0.2 (0.5)	-0.3 (0.9)	0.6 (1.4)	0.1 (1.4)	
Median	0.0	0.0	0.0	0.0	
[Minimum, Maximum]	[0, 2]	[-3, 2]	[0, 6]	[-3, 4]	
Day3 Second time					
Number of participants	39	39	36	35	p=0.415
Mean (standard deviation)	0.3 (0.7)	-0.2 (0.8)	0.5 (1.1)	0.0 (1.2)	
Median	0.0	0.0	0.0	0.0	
[Minimum, Maximum]	[0, 2]	[-2, 2]	[0, 4]	[-3, 4]	
Day4 First time					
Number of participants	40	40	39	36	p=0.256
Mean (standard deviation)	0.3 (0.7)	-0.2 (1.0)	0.5 (0.9)	-0.1 (0.8)	
Median	0.0	0.0	0.0	0.0	
[Minimum, Maximum]	[0, 3]	[-3, 3]	[0, 4]	[-3, 2]	
Day4 Second time					
Number of participants	39	39	39	36	p=0.620
Mean (standard deviation)	0.4 (1.0)	-0.1 (1.2)	0.4 (0.9)	-0.1 (0.7)	
Median	0.0	0.0	0.0	0.0	
[Minimum, Maximum]	[0, 4]	[-3, 4]	[0, 4]	[-2, 2]	

Diarrhea

Time	EFE group (N=41)		Placebo group (N=40)		Wilcoxon rank sum test
	Measurement	Change	Measurement	Change	
Day5 First time					
Number of participants	40	40	39	36	p=0.153
Mean (standard deviation)	0.2 (0.6)	-0.3 (0.9)	0.3 (0.7)	-0.2 (0.7)	
Median	0.0	0.0	0.0	0.0	
[Minimum, Maximum]	[0, 3]	[-3, 2]	[0, 3]	[-3, 1]	
Day5 Second time					
Number of participants	39	39	35	32	p=0.418
Mean (standard deviation)	0.3 (0.8)	-0.2 (1.0)	0.4 (0.7)	-0.2 (0.9)	
Median	0.0	0.0	0.0	0.0	
[Minimum, Maximum]	[0, 4]	[-3, 3]	[0, 2]	[-3, 2]	
Day6 First time					
Number of participants	40	40	39	36	p=0.146
Mean (standard deviation)	0.2 (0.7)	-0.3 (1.0)	0.3 (0.6)	-0.2 (0.8)	
Median	0.0	0.0	0.0	0.0	
[Minimum, Maximum]	[0, 4]	[-3, 3]	[0, 2]	[-3, 2]	
Day6 Second time					
Number of participants	38	38	36	34	p=0.701
Mean (standard deviation)	0.3 (0.8)	-0.2 (1.1)	0.3 (0.8)	-0.2 (0.8)	
Median	0.0	0.0	0.0	0.0	
[Minimum, Maximum]	[0, 3]	[-3, 3]	[0, 3]	[-3, 2]	

Diarrhea

Time	EFE group (N=41)		Placebo group (N=40)		Wilcoxon rank sum test
	Measurement	Change	Measurement	Change	
Day7 First time					
Number of participants	40	40	39	36	p=0.140
Mean (standard deviation)	0.2 (0.6)	-0.3 (0.9)	0.3 (0.6)	-0.3 (0.8)	
Median	0.0	0.0	0.0	0.0	
[Minimum, Maximum]	[0, 3]	[-3, 2]	[0, 2]	[-3, 2]	
Day7 Second time					
Number of participants	39	39	36	33	p=0.343
Mean (standard deviation)	0.2 (0.7)	-0.3 (0.9)	0.2 (0.5)	-0.3 (0.8)	
Median	0.0	0.0	0.0	0.0	
[Minimum, Maximum]	[0, 3]	[-3, 2]	[0, 2]	[-3, 1]	
Day8 First time					
Number of participants	39	39	36	33	p=0.984
Mean (standard deviation)	0.2 (0.7)	-0.2 (0.9)	0.1 (0.4)	-0.4 (0.8)	
Median	0.0	0.0	0.0	0.0	
[Minimum, Maximum]	[0, 3]	[-3, 2]	[0, 2]	[-3, 1]	
Day8 Second time					
Number of participants	37	37	35	33	p=0.298
Mean (standard deviation)	0.2 (0.6)	-0.3 (0.8)	0.2 (0.5)	-0.2 (0.6)	
Median	0.0	0.0	0.0	0.0	
[Minimum, Maximum]	[0, 3]	[-3, 2]	[0, 2]	[-2, 1]	

Diarrhea

Time	EFE group (N=41)		Placebo group (N=40)		Wilcoxon rank sum test
	Measurement	Change	Measurement	Change	
Day9 First time					
Number of participants	39	39	37	35	p=0.690
Mean (standard deviation)	0.2 (0.7)	-0.2 (0.9)	0.2 (0.6)	-0.3 (1.0)	
Median	0.0	0.0	0.0	0.0	
[Minimum, Maximum]	[0, 3]	[-3, 2]	[0, 2]	[-3, 2]	
Day9 Second time					
Number of participants	37	37	37	35	p=0.444
Mean (standard deviation)	0.1 (0.5)	-0.4 (0.8)	0.1 (0.4)	-0.4 (0.7)	
Median	0.0	0.0	0.0	0.0	
[Minimum, Maximum]	[0, 2]	[-3, 1]	[0, 2]	[-3, 0]	
Day10 First time					
Number of participants	38	38	38	35	p=0.977
Mean (standard deviation)	0.2 (0.5)	-0.3 (0.8)	0.1 (0.4)	-0.3 (0.9)	
Median	0.0	0.0	0.0	0.0	
[Minimum, Maximum]	[0, 2]	[-3, 1]	[0, 2]	[-3, 2]	
Day10 Second time					
Number of participants	36	36	36	33	p=0.953
Mean (standard deviation)	0.2 (0.6)	-0.3 (0.9)	0.1 (0.3)	-0.4 (0.8)	
Median	0.0	0.0	0.0	0.0	
[Minimum, Maximum]	[0, 3]	[-3, 2]	[0, 1]	[-3, 1]	

Diarrhea

Time	EFE group (N=41)		Placebo group (N=40)		Wilcoxon rank sum test
	Measurement	Change	Measurement	Change	
Day11 First time					
Number of participants	39	39	35	33	p=0.654
Mean (standard deviation)	0.1 (0.5)	-0.3 (0.8)	0.1 (0.3)	-0.3 (0.7)	
Median	0.0	0.0	0.0	0.0	
[Minimum, Maximum]	[0, 2]	[-3, 1]	[0, 1]	[-3, 1]	
Day11 Second time					
Number of participants	34	34	34	32	p=0.770
Mean (standard deviation)	0.2 (0.7)	-0.2 (1.0)	0.1 (0.4)	-0.3 (0.7)	
Median	0.0	0.0	0.0	0.0	
[Minimum, Maximum]	[0, 4]	[-3, 3]	[0, 1]	[-3, 1]	
Day12 First time					
Number of participants	38	38	35	33	p=1.000
Mean (standard deviation)	0.2 (0.8)	-0.2 (0.9)	0.1 (0.3)	-0.3 (0.7)	
Median	0.0	0.0	0.0	0.0	
[Minimum, Maximum]	[0, 4]	[-3, 3]	[0, 1]	[-3, 1]	
Day12 Second time					
Number of participants	34	34	33	31	p=0.714
Mean (standard deviation)	0.1 (0.5)	-0.3 (0.8)	0.2 (0.4)	-0.3 (0.8)	
Median	0.0	0.0	0.0	0.0	
[Minimum, Maximum]	[0, 2]	[-3, 1]	[0, 2]	[-3, 1]	

Diarrhea

Time	EFE group (N=41)		Placebo group (N=40)		Wilcoxon rank sum test
	Measurement	Change	Measurement	Change	
Day13 First time					
Number of participants	38	38	36	33	p=0.417
Mean (standard deviation)	0.1 (0.5)	-0.3 (0.7)	0.1 (0.3)	-0.3 (0.8)	
Median	0.0	0.0	0.0	0.0	
[Minimum, Maximum]	[0, 2]	[-3, 1]	[0, 1]	[-3, 1]	
Day13 Second time					
Number of participants	35	35	33	31	p=0.158
Mean (standard deviation)	0.1 (0.5)	-0.3 (0.9)	0.2 (0.7)	-0.2 (1.1)	
Median	0.0	0.0	0.0	0.0	
[Minimum, Maximum]	[0, 3]	[-3, 2]	[0, 4]	[-3, 4]	
Day14 First time					
Number of participants	39	39	34	32	p=0.312
Mean (standard deviation)	0.1 (0.4)	-0.4 (0.8)	0.2 (0.5)	-0.3 (0.9)	
Median	0.0	0.0	0.0	0.0	
[Minimum, Maximum]	[0, 2]	[-3, 1]	[0, 2]	[-3, 2]	
Day14 Second time					
Number of participants	35	35	34	32	p=0.659
Mean (standard deviation)	0.1 (0.4)	-0.3 (0.8)	0.1 (0.3)	-0.4 (0.9)	
Median	0.0	0.0	0.0	0.0	
[Minimum, Maximum]	[0, 2]	[-3, 1]	[0, 1]	[-3, 1]	

Diarrhea

Time	EFE group (N=41)		Placebo group (N=40)		Wilcoxon rank sum test
	Measurement	Change	Measurement	Change	
Day15 First time					
Number of participants	37	37	34	31	p=0.273
Mean (standard deviation)	0.0 (0.2)	-0.4 (0.8)	0.1 (0.3)	-0.4 (0.8)	
Median	0.0	0.0	0.0	0.0	
[Minimum, Maximum]	[0, 1]	[-3, 1]	[0, 1]	[-3, 0]	

Dysgeusia/dysosmia

Time	EFE group (N=41)		Placebo group (N=40)		Wilcoxon rank sum test
	Measurement	Change	Measurement	Change	
Day1 First time					
Number of participants	41		37		
Mean (standard deviation)	1.0 (1.9)		1.1 (2.1)		
Median	0.0		0.0		
[Minimum, Maximum]	[0, 6]		[0, 6]		
Day1 Second time					
Number of participants	41	41	35	35	p=0.984
Mean (standard deviation)	0.9 (1.8)	-0.1 (0.4)	0.9 (1.8)	-0.1 (0.3)	
Median	0.0	0.0	0.0	0.0	
[Minimum, Maximum]	[0, 6]	[-1, 1]	[0, 6]	[-1, 0]	
Day2 First time					
Number of participants	41	41	38	36	p=0.977
Mean (standard deviation)	1.0 (1.7)	0.0 (0.5)	1.0 (1.8)	-0.1 (0.5)	
Median	0.0	0.0	0.0	0.0	
[Minimum, Maximum]	[0, 6]	[-1, 1]	[0, 6]	[-2, 1]	
Day2 Second time					
Number of participants	39	39	37	35	p=0.658
Mean (standard deviation)	1.0 (1.8)	0.0 (0.8)	0.9 (1.7)	-0.3 (0.7)	
Median	0.0	0.0	0.0	0.0	
[Minimum, Maximum]	[0, 6]	[-2, 3]	[0, 6]	[-3, 0]	

Dysgeusia/dysosmia

Time	EFE group (N=41)		Placebo group (N=40)		Wilcoxon rank sum test
	Measurement	Change	Measurement	Change	
Day3 First time					
Number of participants	40	40	38	36	p=0.658
Mean (standard deviation)	1.1 (1.7)	0.0 (0.9)	0.9 (1.7)	-0.3 (0.8)	
Median	0.0	0.0	0.0	0.0	
[Minimum, Maximum]	[0, 6]	[-2, 4]	[0, 6]	[-3, 1]	
Day3 Second time					
Number of participants	39	39	36	35	p=0.735
Mean (standard deviation)	0.9 (1.7)	0.0 (1.3)	0.9 (1.6)	-0.1 (1.3)	
Median	0.0	0.0	0.0	0.0	
[Minimum, Maximum]	[0, 6]	[-4, 4]	[0, 5]	[-3, 5]	
Day4 First time					
Number of participants	40	40	39	36	p=0.841
Mean (standard deviation)	1.1 (1.7)	0.0 (1.1)	1.1 (1.8)	-0.1 (1.3)	
Median	0.0	0.0	0.0	0.0	
[Minimum, Maximum]	[0, 5]	[-3, 4]	[0, 6]	[-3, 6]	
Day4 Second time					
Number of participants	39	39	39	36	p=0.683
Mean (standard deviation)	1.0 (1.5)	-0.1 (1.2)	0.9 (1.6)	-0.2 (1.5)	
Median	0.0	0.0	0.0	0.0	
[Minimum, Maximum]	[0, 5]	[-4, 4]	[0, 6]	[-3, 6]	

Dysgeusia/dysosmia

Time	EFE group (N=41)		Placebo group (N=40)		Wilcoxon rank sum test
	Measurement	Change	Measurement	Change	
Day5 First time					
Number of participants	40	40	39	36	p=0.433
Mean (standard deviation)	1.1 (1.6)	0.1 (1.6)	0.9 (1.6)	-0.3 (1.5)	
Median	0.0	0.0	0.0	0.0	
[Minimum, Maximum]	[0, 6]	[-4, 6]	[0, 6]	[-4, 6]	
Day5 Second time					
Number of participants	39	39	36	33	p=0.532
Mean (standard deviation)	1.0 (1.6)	0.1 (1.7)	0.9 (1.7)	-0.2 (1.4)	
Median	0.0	0.0	0.0	0.0	
[Minimum, Maximum]	[0, 6]	[-4, 6]	[0, 6]	[-3, 6]	
Day6 First time					
Number of participants	40	40	39	36	p=0.311
Mean (standard deviation)	1.1 (1.6)	0.1 (1.8)	0.8 (1.5)	-0.3 (1.5)	
Median	0.0	0.0	0.0	0.0	
[Minimum, Maximum]	[0, 6]	[-5, 6]	[0, 6]	[-3, 6]	
Day6 Second time					
Number of participants	38	38	36	34	p=0.238
Mean (standard deviation)	0.9 (1.5)	-0.1 (1.9)	0.7 (1.5)	-0.3 (1.6)	
Median	0.0	0.0	0.0	0.0	
[Minimum, Maximum]	[0, 6]	[-6, 6]	[0, 6]	[-4, 6]	

Dysgeusia/dysosmia

Time	EFE group (N=41)		Placebo group (N=40)		Wilcoxon rank sum test
	Measurement	Change	Measurement	Change	
Day7 First time					
Number of participants	40	40	39	36	p=0.232
Mean (standard deviation)	1.0 (1.5)	-0.1 (1.8)	0.7 (1.5)	-0.4 (1.6)	
Median	0.0	0.0	0.0	0.0	
[Minimum, Maximum]	[0, 6]	[-5, 6]	[0, 6]	[-4, 6]	
Day7 Second time					
Number of participants	39	39	36	33	p=0.466
Mean (standard deviation)	0.8 (1.3)	-0.1 (1.9)	0.8 (1.5)	-0.4 (1.6)	
Median	0.0	0.0	0.0	0.0	
[Minimum, Maximum]	[0, 6]	[-6, 6]	[0, 6]	[-4, 6]	
Day8 First time					
Number of participants	39	39	36	33	p=0.298
Mean (standard deviation)	0.8 (1.5)	-0.2 (1.8)	0.7 (1.4)	-0.3 (1.5)	
Median	0.0	0.0	0.0	0.0	
[Minimum, Maximum]	[0, 6]	[-5, 6]	[0, 6]	[-3, 6]	
Day8 Second time					
Number of participants	37	37	35	33	p=0.737
Mean (standard deviation)	0.8 (1.4)	-0.2 (1.9)	0.7 (1.4)	-0.3 (1.6)	
Median	0.0	0.0	0.0	0.0	
[Minimum, Maximum]	[0, 6]	[-6, 6]	[0, 6]	[-3, 6]	

Dysgeusia/dysosmia

Time	EFE group (N=41)		Placebo group (N=40)		Wilcoxon rank sum test
	Measurement	Change	Measurement	Change	
Day9 First time					
Number of participants	39	39	37	35	p=0.886
Mean (standard deviation)	0.7 (1.4)	-0.3 (2.0)	0.8 (1.5)	-0.3 (1.6)	
Median	0.0	0.0	0.0	0.0	
[Minimum, Maximum]	[0, 6]	[-6, 6]	[0, 6]	[-4, 6]	
Day9 Second time					
Number of participants	37	37	37	35	p=0.698
Mean (standard deviation)	0.6 (1.5)	-0.3 (1.9)	0.6 (1.3)	-0.5 (1.6)	
Median	0.0	0.0	0.0	0.0	
[Minimum, Maximum]	[0, 6]	[-6, 6]	[0, 5]	[-4, 5]	
Day10 First time					
Number of participants	38	38	38	35	p=0.833
Mean (standard deviation)	0.7 (1.4)	-0.4 (1.9)	0.6 (1.3)	-0.5 (1.6)	
Median	0.0	0.0	0.0	0.0	
[Minimum, Maximum]	[0, 6]	[-5, 6]	[0, 5]	[-4, 5]	
Day10 Second time					
Number of participants	36	36	36	33	p=0.994
Mean (standard deviation)	0.7 (1.5)	-0.4 (2.0)	0.6 (1.3)	-0.5 (1.7)	
Median	0.0	0.0	0.0	0.0	
[Minimum, Maximum]	[0, 6]	[-6, 6]	[0, 5]	[-5, 5]	

Dysgeusia/dysosmia

Time	EFE group (N=41)		Placebo group (N=40)		Wilcoxon rank sum test
	Measurement	Change	Measurement	Change	
Day11 First time					
Number of participants	39	39	35	33	p=0.972
Mean (standard deviation)	0.6 (1.4)	-0.4 (1.9)	0.7 (1.3)	-0.5 (1.7)	
Median	0.0	0.0	0.0	0.0	
[Minimum, Maximum]	[0, 6]	[-5, 6]	[0, 5]	[-5, 5]	
Day11 Second time					
Number of participants	34	34	34	32	p=0.761
Mean (standard deviation)	0.5 (1.3)	-0.5 (2.1)	0.6 (1.3)	-0.6 (1.8)	
Median	0.0	0.0	0.0	0.0	
[Minimum, Maximum]	[0, 6]	[-6, 6]	[0, 5]	[-5, 5]	
Day12 First time					
Number of participants	38	38	35	33	p=0.798
Mean (standard deviation)	0.6 (1.4)	-0.4 (2.0)	0.6 (1.4)	-0.6 (1.8)	
Median	0.0	0.0	0.0	0.0	
[Minimum, Maximum]	[0, 6]	[-5, 6]	[0, 5]	[-5, 5]	
Day12 Second time					
Number of participants	34	34	33	31	p=0.455
Mean (standard deviation)	0.4 (0.9)	-0.4 (1.7)	0.7 (1.4)	-0.6 (1.7)	
Median	0.0	0.0	0.0	0.0	
[Minimum, Maximum]	[0, 3]	[-6, 3]	[0, 5]	[-5, 5]	

Dysgeusia/dysosmia

Time	EFE group (N=41)		Placebo group (N=40)		Wilcoxon rank sum test
	Measurement	Change	Measurement	Change	
Day13 First time					
Number of participants	38	38	36	33	p=0.752
Mean (standard deviation)	0.4 (0.9)	-0.7 (1.9)	0.6 (1.4)	-0.5 (1.7)	
Median	0.0	0.0	0.0	0.0	
[Minimum, Maximum]	[0, 3]	[-6, 3]	[0, 5]	[-5, 5]	
Day13 Second time					
Number of participants	35	35	33	31	p=0.754
Mean (standard deviation)	0.3 (0.8)	-0.8 (1.9)	0.6 (1.4)	-0.6 (1.9)	
Median	0.0	0.0	0.0	0.0	
[Minimum, Maximum]	[0, 3]	[-6, 3]	[0, 5]	[-5, 5]	
Day14 First time					
Number of participants	39	39	34	32	p=0.608
Mean (standard deviation)	0.3 (0.7)	-0.7 (1.7)	0.6 (1.3)	-0.5 (1.6)	
Median	0.0	0.0	0.0	0.0	
[Minimum, Maximum]	[0, 3]	[-5, 3]	[0, 5]	[-5, 4]	
Day14 Second time					
Number of participants	35	35	34	32	p=0.594
Mean (standard deviation)	0.3 (0.8)	-0.8 (1.9)	0.6 (1.3)	-0.5 (1.6)	
Median	0.0	0.0	0.0	0.0	
[Minimum, Maximum]	[0, 3]	[-6, 3]	[0, 5]	[-5, 4]	

Dysgeusia/dysosmia

Time	EFE group (N=41)		Placebo group (N=40)		Wilcoxon rank sum test
	Measurement	Change	Measurement	Change	
Day15 First time					
Number of participants	37	37	34	31	p=0.699
Mean (standard deviation)	0.3 (0.7)	-0.6 (1.8)	0.6 (1.3)	-0.5 (1.7)	
Median	0.0	0.0	0.0	0.0	
[Minimum, Maximum]	[0, 3]	[-6, 3]	[0, 5]	[-5, 4]	

Headaches

Time	EFE group (N=41)		Placebo group (N=40)		Wilcoxon rank sum test
	Measurement	Change	Measurement	Change	
Day1 First time					
Number of participants	29		30		
Mean (standard deviation)	1.4 (1.2)		1.2 (1.4)		
Median	1.0		1.0		
[Minimum, Maximum]	[0, 4]		[0, 4]		
Day1 Second time					
Number of participants	24	24	22	22	p=0.927
Mean (standard deviation)	1.2 (1.2)	-0.3 (0.7)	1.2 (1.3)	0.0 (1.1)	
Median	1.0	0.0	1.0	0.0	
[Minimum, Maximum]	[0, 4]	[-2, 1]	[0, 3]	[-1, 3]	
Day2 First time					
Number of participants	20	20	20	19	p=0.498
Mean (standard deviation)	1.2 (1.3)	-0.3 (1.0)	1.7 (1.8)	0.4 (1.7)	
Median	1.0	-0.5	1.0	0.0	
[Minimum, Maximum]	[0, 4]	[-2, 2]	[0, 5]	[-2, 5]	
Day2 Second time					
Number of participants	19	19	19	18	p=0.058
Mean (standard deviation)	0.5 (0.7)	-0.9 (1.0)	1.3 (1.4)	0.2 (1.8)	
Median	0.0	-1.0	1.0	-0.5	
[Minimum, Maximum]	[0, 2]	[-3, 1]	[0, 5]	[-2, 5]	

Headaches

Time	EFE group (N=41)		Placebo group (N=40)		Wilcoxon rank sum test
	Measurement	Change	Measurement	Change	
Day3 First time					
Number of participants	19	19	19	18	p=0.755
Mean (standard deviation)	0.6 (1.0)	-0.7 (1.1)	0.8 (1.2)	-0.3 (1.7)	
Median	0.0	-1.0	0.0	0.0	
[Minimum, Maximum]	[0, 3]	[-3, 1]	[0, 4]	[-4, 4]	
Day3 Second time					
Number of participants	19	19	19	18	p=0.393
Mean (standard deviation)	0.7 (0.9)	-0.7 (1.3)	0.9 (1.0)	-0.2 (1.6)	
Median	0.0	-1.0	1.0	0.0	
[Minimum, Maximum]	[0, 3]	[-3, 3]	[0, 3]	[-3, 3]	
Day4 First time					
Number of participants	19	19	19	18	p=0.071
Mean (standard deviation)	0.4 (0.8)	-1.0 (1.2)	0.8 (1.0)	-0.4 (1.5)	
Median	0.0	-1.0	1.0	0.0	
[Minimum, Maximum]	[0, 3]	[-3, 1]	[0, 3]	[-3, 3]	
Day4 Second time					
Number of participants	18	18	19	18	p=0.056
Mean (standard deviation)	0.3 (0.6)	-0.9 (1.2)	1.1 (1.4)	-0.1 (2.2)	
Median	0.0	-1.0	1.0	0.0	
[Minimum, Maximum]	[0, 2]	[-3, 1]	[0, 5]	[-4, 5]	

Headaches

Time	EFE group (N=41)		Placebo group (N=40)		Wilcoxon rank sum test
	Measurement	Change	Measurement	Change	
Day5 First time					
Number of participants	19	19	19	18	p=0.383
Mean (standard deviation)	0.6 (1.0)	-0.7 (1.2)	0.9 (1.0)	-0.3 (1.6)	
Median	0.0	-1.0	1.0	0.0	
[Minimum, Maximum]	[0, 3]	[-3, 1]	[0, 3]	[-3, 3]	
Day5 Second time					
Number of participants	18	18	17	16	p=0.153
Mean (standard deviation)	0.3 (0.7)	-0.9 (1.2)	0.9 (1.3)	-0.3 (2.2)	
Median	0.0	-1.0	0.0	0.0	
[Minimum, Maximum]	[0, 2]	[-3, 1]	[0, 4]	[-4, 4]	
Day6 First time					
Number of participants	18	18	18	17	p=0.057
Mean (standard deviation)	0.2 (0.5)	-1.1 (1.1)	0.9 (1.4)	-0.2 (2.1)	
Median	0.0	-1.0	0.0	0.0	
[Minimum, Maximum]	[0, 2]	[-3, 1]	[0, 5]	[-4, 5]	
Day6 Second time					
Number of participants	17	17	17	16	p=0.138
Mean (standard deviation)	0.4 (0.6)	-0.9 (1.1)	1.1 (1.3)	-0.2 (2.0)	
Median	0.0	-1.0	0.0	0.0	
[Minimum, Maximum]	[0, 2]	[-3, 1]	[0, 4]	[-4, 4]	

Headaches

Time	EFE group (N=41)		Placebo group (N=40)		Wilcoxon rank sum test
	Measurement	Change	Measurement	Change	
Day7 First time					
Number of participants	17	17	18	17	p=0.443
Mean (standard deviation)	0.4 (0.7)	-0.9 (1.1)	0.7 (1.2)	-0.5 (2.0)	
Median	0.0	-1.0	0.0	0.0	
[Minimum, Maximum]	[0, 2]	[-3, 1]	[0, 4]	[-4, 4]	
Day7 Second time					
Number of participants	17	17	17	16	p=0.235
Mean (standard deviation)	0.3 (0.6)	-0.9 (1.1)	0.7 (1.0)	-0.6 (1.8)	
Median	0.0	-1.0	0.0	0.0	
[Minimum, Maximum]	[0, 2]	[-3, 1]	[0, 3]	[-4, 3]	
Day8 First time					
Number of participants	16	16	17	16	p=0.073
Mean (standard deviation)	0.2 (0.5)	-1.1 (1.0)	0.7 (1.0)	-0.6 (1.8)	
Median	0.0	-1.0	0.0	0.0	
[Minimum, Maximum]	[0, 2]	[-3, 0]	[0, 3]	[-4, 3]	
Day8 Second time					
Number of participants	15	15	16	16	p=0.059
Mean (standard deviation)	0.2 (0.8)	-1.1 (1.1)	0.8 (1.2)	-0.4 (1.8)	
Median	0.0	-1.0	0.0	0.0	
[Minimum, Maximum]	[0, 3]	[-3, 0]	[0, 4]	[-4, 4]	

Headaches

Time	EFE group (N=41)		Placebo group (N=40)		Wilcoxon rank sum test
	Measurement	Change	Measurement	Change	
Day9 First time					
Number of participants	16	16	17	17	p=0.449
Mean (standard deviation)	0.3 (0.6)	-1.0 (1.0)	0.6 (1.1)	-0.6 (1.9)	
Median	0.0	-1.0	0.0	0.0	
[Minimum, Maximum]	[0, 2]	[-3, 0]	[0, 4]	[-4, 4]	
Day9 Second time					
Number of participants	16	16	17	17	p=0.082
Mean (standard deviation)	0.1 (0.3)	-1.3 (1.0)	0.6 (1.1)	-0.6 (1.8)	
Median	0.0	-1.0	0.0	0.0	
[Minimum, Maximum]	[0, 1]	[-3, 0]	[0, 4]	[-4, 4]	
Day10 First time					
Number of participants	15	15	18	17	p=0.312
Mean (standard deviation)	0.4 (0.6)	-1.0 (0.9)	1.0 (1.5)	-0.2 (2.3)	
Median	0.0	-1.0	0.0	0.0	
[Minimum, Maximum]	[0, 2]	[-3, 0]	[0, 5]	[-4, 5]	
Day10 Second time					
Number of participants	14	14	16	15	p=0.095
Mean (standard deviation)	0.1 (0.3)	-1.2 (1.1)	0.6 (1.0)	-0.8 (1.8)	
Median	0.0	-1.0	0.0	0.0	
[Minimum, Maximum]	[0, 1]	[-3, 0]	[0, 3]	[-4, 3]	

Headaches

Time	EFE group (N=41)		Placebo group (N=40)		Wilcoxon rank sum test
	Measurement	Change	Measurement	Change	
Day11 First time					
Number of participants	16	16	16	16	p=0.639
Mean (standard deviation)	0.3 (0.6)	-1.1 (1.0)	0.4 (0.9)	-0.9 (1.8)	
Median	0.0	-1.0	0.0	-0.5	
[Minimum, Maximum]	[0, 2]	[-3, 0]	[0, 3]	[-4, 3]	
Day11 Second time					
Number of participants	15	15	15	15	p=0.161
Mean (standard deviation)	0.1 (0.4)	-1.3 (1.0)	0.5 (0.8)	-0.8 (1.9)	
Median	0.0	-1.0	0.0	0.0	
[Minimum, Maximum]	[0, 1]	[-3, 0]	[0, 2]	[-4, 2]	
Day12 First time					
Number of participants	16	16	16	16	p=0.421
Mean (standard deviation)	0.2 (0.5)	-1.1 (1.1)	0.3 (0.6)	-1.0 (1.7)	
Median	0.0	-1.0	0.0	-1.0	
[Minimum, Maximum]	[0, 2]	[-3, 1]	[0, 2]	[-4, 2]	
Day12 Second time					
Number of participants	13	13	15	15	p=0.053
Mean (standard deviation)	0.0 (0.0)	-1.2 (1.1)	0.4 (0.7)	-1.0 (1.8)	
Median	0.0	-1.0	0.0	-1.0	
[Minimum, Maximum]	[0, 0]	[-3, 0]	[0, 2]	[-4, 2]	

Headaches

Time	EFE group (N=41)		Placebo group (N=40)		Wilcoxon rank sum test
	Measurement	Change	Measurement	Change	
Day13 First time					
Number of participants	15	15	16	15	p=0.378
Mean (standard deviation)	0.1 (0.5)	-1.2 (1.0)	0.3 (0.6)	-1.1 (1.6)	
Median	0.0	-1.0	0.0	-1.0	
[Minimum, Maximum]	[0, 2]	[-3, 0]	[0, 2]	[-4, 2]	
Day13 Second time					
Number of participants	14	14	14	14	p=0.970
Mean (standard deviation)	0.1 (0.4)	-1.1 (0.9)	0.3 (0.8)	-1.2 (1.8)	
Median	0.0	-1.0	0.0	-1.0	
[Minimum, Maximum]	[0, 1]	[-3, 0]	[0, 3]	[-4, 3]	
Day14 First time					
Number of participants	15	15	15	15	p=0.074
Mean (standard deviation)	0.1 (0.3)	-1.3 (1.0)	0.5 (0.8)	-0.9 (1.9)	
Median	0.0	-1.0	0.0	-1.0	
[Minimum, Maximum]	[0, 1]	[-3, 0]	[0, 3]	[-4, 3]	
Day14 Second time					
Number of participants	14	14	15	15	p=0.740
Mean (standard deviation)	0.3 (0.8)	-1.1 (1.2)	0.3 (0.7)	-1.1 (1.7)	
Median	0.0	-1.0	0.0	-1.0	
[Minimum, Maximum]	[0, 3]	[-3, 1]	[0, 2]	[-4, 2]	

Headaches

Time	EFE group (N=41)		Placebo group (N=40)		Wilcoxon rank sum test
	Measurement	Change	Measurement	Change	
Day15 First time					
Number of participants	13	13	14	13	p=0.594
Mean (standard deviation)	0.1 (0.3)	-1.2 (1.1)	0.2 (0.6)	-1.2 (1.7)	
Median	0.0	-1.0	0.0	-1.0	
[Minimum, Maximum]	[0, 1]	[-3, 0]	[0, 2]	[-4, 2]	

Sore throat

Time	EFE group (N=41)		Placebo group (N=40)		Wilcoxon rank sum test
	Measurement	Change	Measurement	Change	
Day1 First time					
Number of participants	29		30		
Mean (standard deviation)	3.0 (1.7)		2.6 (1.8)		
Median	3.0		3.0		
[Minimum, Maximum]	[0, 6]		[0, 6]		
Day1 Second time					
Number of participants	24	24	22	22	p=0.490
Mean (standard deviation)	2.6 (2.0)	-0.3 (0.9)	2.2 (2.0)	-0.5 (0.9)	
Median	2.0	0.0	2.0	0.0	
[Minimum, Maximum]	[0, 6]	[-3, 2]	[0, 6]	[-2, 2]	
Day2 First time					
Number of participants	20	20	20	19	p=0.132
Mean (standard deviation)	2.3 (1.6)	-0.5 (1.1)	1.5 (1.6)	-0.6 (0.9)	
Median	2.0	-0.5	1.0	-1.0	
[Minimum, Maximum]	[0, 5]	[-3, 1]	[0, 4]	[-3, 1]	
Day2 Second time					
Number of participants	19	19	19	18	p=0.159
Mean (standard deviation)	1.7 (1.7)	-0.9 (1.1)	1.0 (1.2)	-1.1 (0.9)	
Median	1.0	-1.0	1.0	-1.0	
[Minimum, Maximum]	[0, 6]	[-3, 0]	[0, 3]	[-3, 0]	

Sore throat

Time	EFE group (N=41)		Placebo group (N=40)		Wilcoxon rank sum test
	Measurement	Change	Measurement	Change	
Day3 First time					
Number of participants	19	19	19	18	p=0.131
Mean (standard deviation)	1.7 (1.4)	-1.1 (1.5)	1.1 (1.2)	-1.0 (1.4)	
Median	2.0	-1.0	1.0	-1.0	
[Minimum, Maximum]	[0, 5]	[-4, 2]	[0, 3]	[-3, 3]	
Day3 Second time					
Number of participants	19	19	19	18	p=0.070
Mean (standard deviation)	1.4 (1.4)	-1.4 (1.5)	0.8 (1.1)	-1.3 (1.4)	
Median	1.0	-1.0	0.0	-1.0	
[Minimum, Maximum]	[0, 5]	[-4, 1]	[0, 4]	[-3, 2]	
Day4 First time					
Number of participants	19	19	19	18	p=0.267
Mean (standard deviation)	1.3 (1.3)	-1.5 (1.6)	0.8 (1.0)	-1.2 (1.4)	
Median	1.0	-1.0	1.0	-1.0	
[Minimum, Maximum]	[0, 5]	[-5, 1]	[0, 3]	[-3, 2]	
Day4 Second time					
Number of participants	18	18	19	18	p=0.044
Mean (standard deviation)	1.2 (1.2)	-1.7 (1.5)	0.6 (1.0)	-1.5 (1.2)	
Median	1.0	-1.5	0.0	-1.5	
[Minimum, Maximum]	[0, 4]	[-4, 1]	[0, 3]	[-3, 1]	

Sore throat

Time	EFE group (N=41)		Placebo group (N=40)		Wilcoxon rank sum test
	Measurement	Change	Measurement	Change	
Day5 First time					
Number of participants	19	19	19	18	p=0.095
Mean (standard deviation)	1.2 (1.3)	-1.7 (1.6)	0.6 (1.0)	-1.5 (1.2)	
Median	1.0	-1.0	0.0	-1.5	
[Minimum, Maximum]	[0, 4]	[-5, 2]	[0, 3]	[-3, 1]	
Day5 Second time					
Number of participants	18	18	17	16	p=0.055
Mean (standard deviation)	0.9 (1.3)	-1.9 (1.6)	0.4 (0.9)	-1.6 (1.4)	
Median	1.0	-1.0	0.0	-1.5	
[Minimum, Maximum]	[0, 5]	[-5, 0]	[0, 3]	[-3, 1]	
Day6 First time					
Number of participants	18	18	18	17	p=0.749
Mean (standard deviation)	0.8 (1.1)	-2.1 (1.6)	0.7 (1.0)	-1.5 (1.2)	
Median	0.0	-2.0	0.0	-2.0	
[Minimum, Maximum]	[0, 4]	[-5, 0]	[0, 3]	[-3, 1]	
Day6 Second time					
Number of participants	17	17	17	16	p=0.722
Mean (standard deviation)	0.4 (0.9)	-2.3 (1.6)	0.5 (1.0)	-1.6 (1.4)	
Median	0.0	-2.0	0.0	-2.0	
[Minimum, Maximum]	[0, 3]	[-5, 0]	[0, 3]	[-3, 1]	

Sore throat

Time	EFE group (N=41)		Placebo group (N=40)		Wilcoxon rank sum test
	Measurement	Change	Measurement	Change	
Day7 First time					
Number of participants	17	17	18	17	p=0.508
Mean (standard deviation)	0.6 (0.7)	-2.1 (1.7)	0.5 (0.9)	-1.6 (1.3)	
Median	0.0	-2.0	0.0	-2.0	
[Minimum, Maximum]	[0, 2]	[-5, 0]	[0, 3]	[-3, 1]	
Day7 Second time					
Number of participants	16	16	17	16	p=0.544
Mean (standard deviation)	0.7 (0.8)	-2.0 (1.8)	0.6 (0.9)	-1.6 (1.5)	
Median	0.5	-2.0	0.0	-1.5	
[Minimum, Maximum]	[0, 2]	[-5, 0]	[0, 3]	[-3, 1]	
Day8 First time					
Number of participants	16	16	17	16	p=0.962
Mean (standard deviation)	0.3 (0.6)	-2.3 (1.7)	0.4 (0.8)	-1.8 (1.5)	
Median	0.0	-2.0	0.0	-2.0	
[Minimum, Maximum]	[0, 2]	[-5, 0]	[0, 3]	[-4, 1]	
Day8 Second time					
Number of participants	15	15	16	16	p=1.000
Mean (standard deviation)	0.5 (0.6)	-2.3 (1.6)	0.6 (0.9)	-1.6 (1.7)	
Median	0.0	-2.0	0.0	-1.5	
[Minimum, Maximum]	[0, 2]	[-5, 0]	[0, 3]	[-4, 1]	

Sore throat

Time	EFE group (N=41)		Placebo group (N=40)		Wilcoxon rank sum test
	Measurement	Change	Measurement	Change	
Day9 First time					
Number of participants	16	16	17	17	p=0.849
Mean (standard deviation)	0.5 (0.7)	-2.1 (1.7)	0.5 (1.0)	-1.6 (1.7)	
Median	0.0	-2.0	0.0	-2.0	
[Minimum, Maximum]	[0, 2]	[-5, 0]	[0, 4]	[-4, 2]	
Day9 Second time					
Number of participants	16	16	17	17	p=0.275
Mean (standard deviation)	0.2 (0.4)	-2.4 (1.8)	0.5 (1.0)	-1.6 (1.7)	
Median	0.0	-2.0	0.0	-2.0	
[Minimum, Maximum]	[0, 1]	[-6, 0]	[0, 4]	[-4, 2]	
Day10 First time					
Number of participants	15	15	18	17	p=0.948
Mean (standard deviation)	0.4 (0.6)	-2.1 (1.8)	0.4 (0.7)	-1.7 (1.4)	
Median	0.0	-2.0	0.0	-2.0	
[Minimum, Maximum]	[0, 2]	[-5, 0]	[0, 2]	[-4, 1]	
Day10 Second time					
Number of participants	14	14	16	15	p=0.920
Mean (standard deviation)	0.4 (0.5)	-2.2 (1.6)	0.4 (0.6)	-1.7 (1.7)	
Median	0.0	-2.0	0.0	-2.0	
[Minimum, Maximum]	[0, 1]	[-5, 0]	[0, 2]	[-4, 1]	

Sore throat

Time	EFE group (N=41)		Placebo group (N=40)		Wilcoxon rank sum test
	Measurement	Change	Measurement	Change	
Day11 First time					
Number of participants	16	16	16	16	p=0.182
Mean (standard deviation)	0.4 (0.7)	-2.2 (1.7)	0.1 (0.3)	-1.9 (1.5)	
Median	0.0	-2.0	0.0	-2.0	
[Minimum, Maximum]	[0, 2]	[-5, 1]	[0, 1]	[-4, 1]	
Day11 Second time					
Number of participants	15	15	14	14	p=0.957
Mean (standard deviation)	0.4 (0.6)	-2.1 (1.6)	0.5 (0.9)	-1.6 (1.9)	
Median	0.0	-2.0	0.0	-2.0	
[Minimum, Maximum]	[0, 2]	[-5, 0]	[0, 3]	[-4, 2]	
Day12 First time					
Number of participants	16	16	16	16	p=0.830
Mean (standard deviation)	0.4 (0.6)	-2.3 (1.6)	0.4 (0.7)	-1.7 (1.8)	
Median	0.0	-2.0	0.0	-1.5	
[Minimum, Maximum]	[0, 2]	[-5, 0]	[0, 2]	[-5, 2]	
Day12 Second time					
Number of participants	13	13	15	15	p=0.303
Mean (standard deviation)	0.5 (1.1)	-2.4 (1.4)	0.2 (0.6)	-1.9 (1.8)	
Median	0.0	-2.0	0.0	-2.0	
[Minimum, Maximum]	[0, 4]	[-5, 0]	[0, 2]	[-5, 2]	

Sore throat

Time	EFE group (N=41)		Placebo group (N=40)		Wilcoxon rank sum test
	Measurement	Change	Measurement	Change	
Day13 First time					
Number of participants	15	15	16	15	p=0.408
Mean (standard deviation)	0.4 (0.6)	-2.3 (1.5)	0.3 (0.6)	-1.9 (1.4)	
Median	0.0	-2.0	0.0	-2.0	
[Minimum, Maximum]	[0, 2]	[-5, 0]	[0, 2]	[-4, 0]	
Day13 Second time					
Number of participants	14	14	14	14	p=0.351
Mean (standard deviation)	0.4 (0.6)	-2.1 (1.5)	0.1 (0.4)	-2.1 (1.3)	
Median	0.0	-2.0	0.0	-2.0	
[Minimum, Maximum]	[0, 2]	[-5, 0]	[0, 1]	[-4, 0]	
Day14 First time					
Number of participants	15	15	15	15	p=0.348
Mean (standard deviation)	0.5 (0.7)	-2.2 (1.7)	0.2 (0.4)	-2.0 (1.3)	
Median	0.0	-2.0	0.0	-2.0	
[Minimum, Maximum]	[0, 2]	[-5, 1]	[0, 1]	[-4, 0]	
Day14 Second time					
Number of participants	14	14	15	15	p=0.370
Mean (standard deviation)	0.4 (0.6)	-2.4 (1.5)	0.2 (0.6)	-2.0 (1.4)	
Median	0.0	-2.0	0.0	-2.0	
[Minimum, Maximum]	[0, 2]	[-5, 0]	[0, 2]	[-4, 0]	

Sore throat

Time	EFE group (N=41)		Placebo group (N=40)		Wilcoxon rank sum test
	Measurement	Change	Measurement	Change	
Day15 First time					
Number of participants	13	13	14	13	p=0.635
Mean (standard deviation)	0.4 (0.7)	-2.5 (1.4)	0.3 (0.6)	-1.8 (1.2)	
Median	0.0	-2.0	0.0	-2.0	
[Minimum, Maximum]	[0, 2]	[-5, -1]	[0, 2]	[-3, 0]	

Muscle pain/Joint pain

Time	EFE group (N=41)		Placebo group (N=40)		Wilcoxon rank sum test
	Measurement	Change	Measurement	Change	
Day1 First time					
Number of participants	29		30		
Mean (standard deviation)	1.2 (1.6)		1.7 (1.7)		
Median	0.0		2.0		
[Minimum, Maximum]	[0, 5]		[0, 5]		
Day1 Second time					
Number of participants	24	24	22	22	p=0.049
Mean (standard deviation)	0.9 (1.4)	-0.3 (0.8)	1.5 (1.3)	-0.3 (0.8)	
Median	0.0	0.0	1.5	0.0	
[Minimum, Maximum]	[0, 5]	[-3, 1]	[0, 4]	[-2, 1]	
Day2 First time					
Number of participants	20	20	20	19	p=0.105
Mean (standard deviation)	0.8 (1.2)	-0.3 (0.9)	1.4 (1.4)	-0.6 (1.0)	
Median	0.0	0.0	1.0	0.0	
[Minimum, Maximum]	[0, 4]	[-2, 1]	[0, 6]	[-2, 1]	
Day2 Second time					
Number of participants	19	19	19	18	p=0.069
Mean (standard deviation)	0.5 (1.0)	-0.7 (1.2)	0.7 (0.7)	-1.1 (1.2)	
Median	0.0	0.0	1.0	-1.0	
[Minimum, Maximum]	[0, 4]	[-3, 1]	[0, 2]	[-4, 0]	

Muscle pain/Joint pain

Time	EFE group (N=41)		Placebo group (N=40)		Wilcoxon rank sum test
	Measurement	Change	Measurement	Change	
Day3 First time					
Number of participants	19	19	19	18	p=0.021
Mean (standard deviation)	0.5 (1.0)	-0.7 (1.1)	1.0 (0.9)	-0.8 (1.2)	
Median	0.0	0.0	1.0	0.0	
[Minimum, Maximum]	[0, 4]	[-3, 1]	[0, 3]	[-3, 1]	
Day3 Second time					
Number of participants	19	19	19	18	p=0.261
Mean (standard deviation)	0.5 (1.0)	-0.7 (1.2)	0.6 (0.8)	-1.2 (1.5)	
Median	0.0	0.0	0.0	-1.0	
[Minimum, Maximum]	[0, 3]	[-3, 2]	[0, 2]	[-5, 1]	
Day4 First time					
Number of participants	19	19	19	18	p=0.395
Mean (standard deviation)	0.5 (1.1)	-0.6 (1.2)	0.6 (0.8)	-1.2 (1.4)	
Median	0.0	0.0	0.0	-1.0	
[Minimum, Maximum]	[0, 4]	[-3, 2]	[0, 2]	[-5, 0]	
Day4 Second time					
Number of participants	18	18	19	18	p=0.612
Mean (standard deviation)	0.3 (0.8)	-0.9 (1.3)	0.3 (0.6)	-1.5 (1.4)	
Median	0.0	0.0	0.0	-1.0	
[Minimum, Maximum]	[0, 3]	[-4, 1]	[0, 2]	[-5, 0]	

Muscle pain/Joint pain

Time	EFE group (N=41)		Placebo group (N=40)		Wilcoxon rank sum test
	Measurement	Change	Measurement	Change	
Day5 First time					
Number of participants	19	19	19	18	p=0.226
Mean (standard deviation)	0.4 (0.8)	-0.8 (1.4)	0.6 (0.8)	-1.2 (1.5)	
Median	0.0	0.0	0.0	-1.0	
[Minimum, Maximum]	[0, 3]	[-4, 2]	[0, 2]	[-5, 1]	
Day5 Second time					
Number of participants	18	18	17	16	p=0.032
Mean (standard deviation)	0.2 (0.7)	-0.9 (1.3)	0.6 (0.7)	-1.4 (1.4)	
Median	0.0	0.0	0.0	-1.0	
[Minimum, Maximum]	[0, 3]	[-4, 0]	[0, 2]	[-5, 0]	
Day6 First time					
Number of participants	18	18	18	17	p=0.144
Mean (standard deviation)	0.2 (0.5)	-1.0 (1.3)	0.4 (0.6)	-1.3 (1.4)	
Median	0.0	0.0	0.0	-1.0	
[Minimum, Maximum]	[0, 2]	[-4, 0]	[0, 2]	[-5, 0]	
Day6 Second time					
Number of participants	17	17	17	16	p=0.270
Mean (standard deviation)	0.2 (0.8)	-1.0 (1.3)	0.3 (0.5)	-1.5 (1.3)	
Median	0.0	0.0	0.0	-1.0	
[Minimum, Maximum]	[0, 3]	[-4, 0]	[0, 1]	[-5, 0]	

Muscle pain/Joint pain

Time	EFE group (N=41)		Placebo group (N=40)		Wilcoxon rank sum test
	Measurement	Change	Measurement	Change	
Day7 First time					
Number of participants	17	17	18	17	p=0.940
Mean (standard deviation)	0.3 (0.8)	-0.9 (1.2)	0.2 (0.5)	-1.5 (1.4)	
Median	0.0	0.0	0.0	-1.0	
[Minimum, Maximum]	[0, 3]	[-4, 0]	[0, 2]	[-5, 0]	
Day7 Second time					
Number of participants	16	16	17	16	p=0.839
Mean (standard deviation)	0.3 (0.8)	-1.0 (1.3)	0.2 (0.4)	-1.6 (1.2)	
Median	0.0	-0.5	0.0	-1.0	
[Minimum, Maximum]	[0, 3]	[-4, 0]	[0, 1]	[-5, 0]	
Day8 First time					
Number of participants	16	16	17	16	p=0.090
Mean (standard deviation)	0.0 (0.0)	-1.0 (1.3)	0.3 (0.7)	-1.5 (1.3)	
Median	0.0	0.0	0.0	-1.0	
[Minimum, Maximum]	[0, 0]	[-4, 0]	[0, 2]	[-5, 0]	
Day8 Second time					
Number of participants	15	15	16	16	p=0.366
Mean (standard deviation)	0.0 (0.0)	-1.1 (1.3)	0.1 (0.5)	-1.6 (1.4)	
Median	0.0	0.0	0.0	-1.5	
[Minimum, Maximum]	[0, 0]	[-4, 0]	[0, 2]	[-5, 0]	

Muscle pain/Joint pain

Time	EFE group (N=41)		Placebo group (N=40)		Wilcoxon rank sum test
	Measurement	Change	Measurement	Change	
Day9 First time					
Number of participants	16	16	17	17	p=1.000
Mean (standard deviation)	0.0 (0.0)	-1.0 (1.3)	0.0 (0.0)	-1.7 (1.3)	
Median	0.0	0.0	0.0	-2.0	
[Minimum, Maximum]	[0, 0]	[-4, 0]	[0, 0]	[-5, 0]	
Day9 Second time					
Number of participants	16	16	17	17	p=0.363
Mean (standard deviation)	0.0 (0.0)	-1.0 (1.3)	0.1 (0.5)	-1.6 (1.3)	
Median	0.0	0.0	0.0	-2.0	
[Minimum, Maximum]	[0, 0]	[-4, 0]	[0, 2]	[-5, 0]	
Day10 First time					
Number of participants	15	15	18	17	p=1.000
Mean (standard deviation)	0.0 (0.0)	-1.1 (1.3)	0.0 (0.0)	-1.7 (1.3)	
Median	0.0	0.0	0.0	-2.0	
[Minimum, Maximum]	[0, 0]	[-4, 0]	[0, 0]	[-5, 0]	
Day10 Second time					
Number of participants	14	14	16	15	p=0.385
Mean (standard deviation)	0.0 (0.0)	-1.1 (1.4)	0.1 (0.5)	-1.7 (1.3)	
Median	0.0	-0.5	0.0	-2.0	
[Minimum, Maximum]	[0, 0]	[-4, 0]	[0, 2]	[-5, 0]	

Muscle pain/Joint pain

Time	EFE group (N=41)		Placebo group (N=40)		Wilcoxon rank sum test
	Measurement	Change	Measurement	Change	
Day11 First time					
Number of participants	16	16	16	16	p=0.349
Mean (standard deviation)	0.0 (0.0)	-1.0 (1.3)	0.1 (0.5)	-1.6 (1.4)	
Median	0.0	0.0	0.0	-2.0	
[Minimum, Maximum]	[0, 0]	[-4, 0]	[0, 2]	[-5, 0]	
Day11 Second time					
Number of participants	15	15	15	15	p=1.000
Mean (standard deviation)	0.0 (0.0)	-1.1 (1.3)	0.0 (0.0)	-1.7 (1.3)	
Median	0.0	0.0	0.0	-2.0	
[Minimum, Maximum]	[0, 0]	[-4, 0]	[0, 0]	[-5, 0]	
Day12 First time					
Number of participants	16	16	16	16	p=0.164
Mean (standard deviation)	0.0 (0.0)	-1.0 (1.3)	0.2 (0.5)	-1.6 (1.4)	
Median	0.0	0.0	0.0	-1.5	
[Minimum, Maximum]	[0, 0]	[-4, 0]	[0, 2]	[-5, 0]	
Day12 Second time					
Number of participants	13	13	15	15	p=0.390
Mean (standard deviation)	0.0 (0.0)	-1.2 (1.4)	0.1 (0.3)	-1.7 (1.3)	
Median	0.0	-1.0	0.0	-2.0	
[Minimum, Maximum]	[0, 0]	[-4, 0]	[0, 1]	[-5, 0]	

Muscle pain/Joint pain

Time	EFE group (N=41)		Placebo group (N=40)		Wilcoxon rank sum test
	Measurement	Change	Measurement	Change	
Day13 First time					
Number of participants	15	15	16	15	p=0.366
Mean (standard deviation)	0.0 (0.0)	-1.1 (1.3)	0.1 (0.5)	-1.5 (1.4)	
Median	0.0	0.0	0.0	-2.0	
[Minimum, Maximum]	[0, 0]	[-4, 0]	[0, 2]	[-5, 0]	
Day13 Second time					
Number of participants	14	14	14	14	p=0.165
Mean (standard deviation)	0.0 (0.0)	-0.9 (1.3)	0.2 (0.6)	-1.6 (1.3)	
Median	0.0	0.0	0.0	-2.0	
[Minimum, Maximum]	[0, 0]	[-4, 0]	[0, 2]	[-5, 0]	
Day14 First time					
Number of participants	15	15	15	15	p=0.164
Mean (standard deviation)	0.0 (0.0)	-1.1 (1.3)	0.1 (0.4)	-1.5 (1.2)	
Median	0.0	0.0	0.0	-2.0	
[Minimum, Maximum]	[0, 0]	[-4, 0]	[0, 1]	[-5, 0]	
Day14 Second time					
Number of participants	14	14	15	15	p=0.370
Mean (standard deviation)	0.0 (0.0)	-1.1 (1.4)	0.1 (0.5)	-1.5 (1.4)	
Median	0.0	0.0	0.0	-2.0	
[Minimum, Maximum]	[0, 0]	[-4, 0]	[0, 2]	[-5, 0]	

Muscle pain/Joint pain

Time	EFE group (N=41)		Placebo group (N=40)		Wilcoxon rank sum test
	Measurement	Change	Measurement	Change	
Day15 First time					
Number of participants	13	13	14	13	p=1.000
Mean (standard deviation)	0.0 (0.0)	-1.1 (1.4)	0.0 (0.0)	-1.7 (1.4)	
Median	0.0	0.0	0.0	-2.0	
[Minimum, Maximum]	[0, 0]	[-4, 0]	[0, 0]	[-5, 0]	

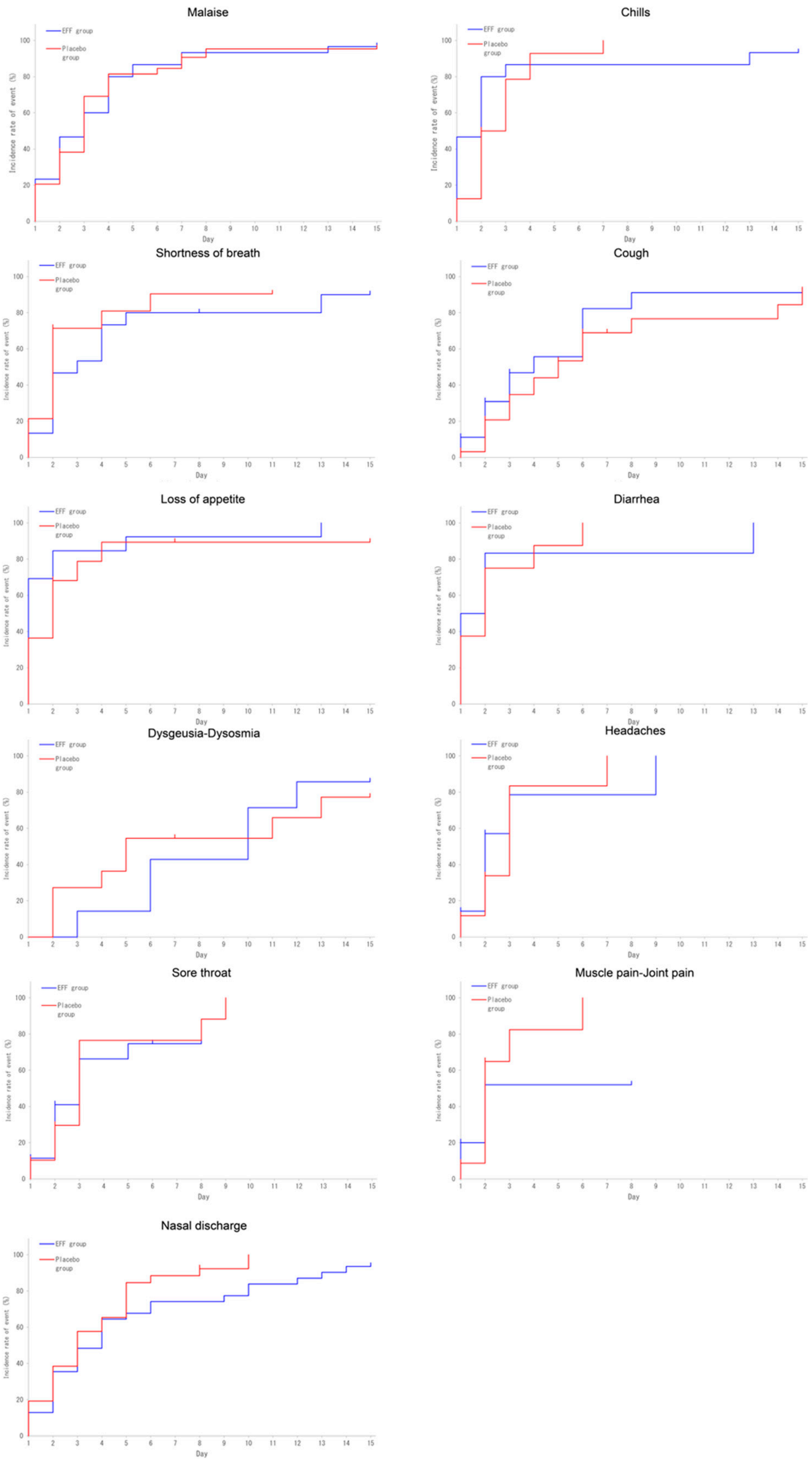

Figure S3: Part 2：Secondary endpoint (5)：Number of days until the FS score for symptoms/pain symptoms becomes 0 or 1 (mITT)

Table S8 Part 2：Secondary endpoint (6) : Non-aggravation rate on Day 21 (Ratio of patients that ended the study without termination from the study due to the symptoms deteriorating and becoming moderate or severe during the study period : Termination criterion 5) »
（mITT）

Time	Group	Target number of participants	Number of participants with non-aggravation (%)	Two-sided 95% confidence interval	Chi-square test
Day 21	EFE group	38	38 (100.0)	90.7–100.0	p=0.146
	Placebo group	37	35 (94.6)	81.8–99.3	

Target number of participants：Excluding patients who used the prohibited concomitant drugs

Table S9 Part 2：Secondary endpoint (7): Ratio of patients without severe condition on Day 15, Day 21 (mITT)

Time	Group	Target number of participants	Number of participants with non-aggravation (%)	Two-sided 95% confidence interval	Chi-square test
Day15	EFE group	38	38 (100.0)	90.7–100.0	p=0.146
	Placebo group	37	35 (94.6)	81.8–99.3	
Day21	EFE group	38	38 (100.0)	90.7–100.0	p=0.146
	Placebo group	37	35 (94.6)	81.8–99.3	

Target number of participants：Excluding patients who used the prohibited concomitant drugs

Severe: Patients who require artificial ventilation (including ECMO)
Source：Table 4.3.5.2 (7)

Table S10 Part 2：Secondary endpoint (8): ”Disease status score on a 6-point ordinal scale” on Day 7, Day 15／At termination and Day 21／1 week after termination（mITT）

Time	EFE group (N=41)<br>Number of participants(%)	Placebo group (N=40)<br>Number of participants(%)	Wilcoxon rank sum test
Day7			
Target number of participants	40	38	
1. Not hospitalized	38 (95.0)	38 (100.0)	p=0.171
2. Hospitalized, does not require oxygen supplementation	2 (5.0)	0 (0.0)	
3. Hospitalized, requires oxygen supplementation	0 (0.0)	0 (0.0)	
4. Hospitalized, with use of noninvasive artificial ventilation or high-flow oxygen therapy device	0 (0.0)	0 (0.0)	
5. Hospitalized, with use of invasive artificial ventilation or ECMO	0 (0.0)	0 (0.0)	
6. Fatal	0 (0.0)	0 (0.0)	
Day15			
Target number of participants	38	35	
1. Not hospitalized	38 (100.0)	35 (100.0)	p=1.000
2. Hospitalized, does not require oxygen supplementation	0 (0.0)	0 (0.0)	
3. Hospitalized, requires oxygen supplementation	0 (0.0)	0 (0.0)	
4. Hospitalized, with use of noninvasive artificial ventilation or high-flow oxygen therapy device	0 (0.0)	0 (0.0)	
5. Hospitalized, with use of invasive artificial ventilation or ECMO	0 (0.0)	0 (0.0)	
6. Fatal	0 (0.0)	0 (0.0)	
Day21			
Target number of participants	38	35	
1. Not hospitalized	38 (100.0)	35 (100.0)	p=1.000
2. Hospitalized, does not require oxygen supplementation	0 (0.0)	0 (0.0)	
3. Hospitalized, requires oxygen supplementation	0 (0.0)	0 (0.0)	
4. Hospitalized, with use of noninvasive artificial ventilation or high-flow oxygen therapy device	0 (0.0)	0 (0.0)	
5. Hospitalized, with use of invasive artificial ventilation or ECMO	0 (0.0)	0 (0.0)	
6. Fatal	0 (0.0)	0 (0.0)	

Time	EFE group (N=41)	Placebo group (N=40)	Wilcoxon rank sum test
	Number of participants(%)	Number of participants(%)	
At termination			
Target number of participants	0	2	
1. Not hospitalized	0 (-)	2 (100.0)	p<0.001
2. Hospitalized, does not require oxygen supplementation	0 (-)	0 (0.0)	
3. Hospitalized, requires oxygen supplementation	0 (-)	0 (0.0)	
4. Hospitalized, with use of noninvasive artificial ventilation or high-flow oxygen therapy device	0 (-)	0 (0.0)	
5. Hospitalized, with use of invasive artificial ventilation or ECMO	0 (-)	0 (0.0)	
6. Fatal	0 (-)	0 (0.0)	
Day15/At termination			
Target number of participants	38	37	
1. Not hospitalized	38 (100.0)	37 (100.0)	p=1.000
2. Hospitalized, does not require oxygen supplementation	0 (0.0)	0 (0.0)	
3. Hospitalized, requires oxygen supplementation	0 (0.0)	0 (0.0)	
4. Hospitalized, with use of noninvasive artificial ventilation or high-flow oxygen therapy device	0 (0.0)	0 (0.0)	
5. Hospitalized, with use of invasive artificial ventilation or ECMO	0 (0.0)	0 (0.0)	
6. Fatal	0 (0.0)	0 (0.0)	
1 week after termination			
Target number of participants	0	2	
1. Not hospitalized	0 (-)	2 (100.0)	p<0.001
2. Hospitalized, does not require oxygen supplementation	0 (-)	0 (0.0)	
3. Hospitalized, requires oxygen supplementation	0 (-)	0 (0.0)	
4. Hospitalized, with use of noninvasive artificial ventilation or high-flow oxygen therapy device	0 (-)	0 (0.0)	
5. Hospitalized, with use of invasive artificial ventilation or ECMO	0 (-)	0 (0.0)	
6. Fatal	0 (-)	0 (0.0)	

Time	EFE group (N=41)	Placebo group (N=40)	Wilcoxon rank sum test
	Number of participants(%)	Number of participants(%)	
Day21/1 week after termination			
Target number of participants	38	37	
1. Not hospitalized	38 (100.0)	37 (100.0)	p=1.000
2. Hospitalized, does not require oxygen supplementation	0 (0.0)	0 (0.0)	
3. Hospitalized, requires oxygen supplementation	0 (0.0)	0 (0.0)	
4. Hospitalized, with use of noninvasive artificial ventilation or high-flow oxygen therapy device	0 (0.0)	0 (0.0)	
5. Hospitalized, with use of invasive artificial ventilation or ECMO	0 (0.0)	0 (0.0)	
6. Fatal	0 (0.0)	0 (0.0)	

Target number of participants：Excluding patients who used the prohibited concomitant drugs
Source：Table 4.3.5.2 (8)

Table S11 Adverse events/side effects by severity for each symptom

	Adverse events									
	EFE group (N=41)									
	Grade 1		Grade 2		Grade 3		Grade 4		Grade 5	
	Number of events	Number of affected participants (%)	Number of events	Number of affected participants (%)	Number of events	Number of affected participants (%)	Number of events	Number of affected participants (%)	Number of events	Number of affected participants (%)
All events	1	1 (2.4)	1	1 (2.4)	0	0 (0.0)	0	0 (0.0)	0	0 (0.0)
Infections and infestations	0	0 (0.0)	0	0 (0.0)	0	0 (0.0)	0	0 (0.0)	0	0 (0.0)
Cystitis	0	0 (0.0)	0	0 (0.0)	0	0 (0.0)	0	0 (0.0)	0	0 (0.0)
Sinusitis	0	0 (0.0)	0	0 (0.0)	0	0 (0.0)	0	0 (0.0)	0	0 (0.0)
Upper respiratory tract infection	0	0 (0.0)	0	0 (0.0)	0	0 (0.0)	0	0 (0.0)	0	0 (0.0)
Pharyngotonsillitis	0	0 (0.0)	0	0 (0.0)	0	0 (0.0)	0	0 (0.0)	0	0 (0.0)
Skin and subcutaneous tissue disorders	0	0 (0.0)	1	1 (2.4)	0	0 (0.0)	0	0 (0.0)	0	0 (0.0)
Eczema	0	0 (0.0)	1	1 (2.4)	0	0 (0.0)	0	0 (0.0)	0	0 (0.0)
Urticaria	0	0 (0.0)	0	0 (0.0)	0	0 (0.0)	0	0 (0.0)	0	0 (0.0)
Laboratory test	1	1 (2.4)	0	0 (0.0)	0	0 (0.0)	0	0 (0.0)	0	0 (0.0)
Abnormal liver function	1	1 (2.4)	0	0 (0.0)	0	0 (0.0)	0	0 (0.0)	0	0 (0.0)

Adverse events

	Placebo group (N=40)									
	Grade 1		Grade 2		Grade 3		Grade 4		Grade 5	
	Number of events	Number of affected participants (%)	Number of events	Number of affected participants (%)	Number of events	Number of affected participants (%)	Number of events	Number of affected participants (%)	Number of events	Number of affected participants (%)
All events	4	4 (10.0)	2	2 (5.0)	0	0 (0.0)	0	0 (0.0)	0	0 (0.0)
Infections and infestations	3	3 (7.5)	1	1 (2.5)	0	0 (0.0)	0	0 (0.0)	0	0 (0.0)
Cystitis	1	1 (2.5)	0	0 (0.0)	0	0 (0.0)	0	0 (0.0)	0	0 (0.0)
Sinusitis	0	0 (0.0)	1	1 (2.5)	0	0 (0.0)	0	0 (0.0)	0	0 (0.0)
Upper respiratory tract infection	1	1 (2.5)	0	0 (0.0)	0	0 (0.0)	0	0 (0.0)	0	0 (0.0)
Pharyngotonsillitis	1	1 (2.5)	0	0 (0.0)	0	0 (0.0)	0	0 (0.0)	0	0 (0.0)
Skin and subcutaneous tissue disorders	0	0 (0.0)	1	1 (2.5)	0	0 (0.0)	0	0 (0.0)	0	0 (0.0)
Eczema	0	0 (0.0)	0	0 (0.0)	0	0 (0.0)	0	0 (0.0)	0	0 (0.0)
Urticaria	0	0 (0.0)	1	1 (2.5)	0	0 (0.0)	0	0 (0.0)	0	0 (0.0)
Laboratory test	1	1 (2.5)	0	0 (0.0)	0	0 (0.0)	0	0 (0.0)	0	0 (0.0)
Abnormal liver function	1	1 (2.5)	0	0 (0.0)	0	0 (0.0)	0	0 (0.0)	0	0 (0.0)

Adverse events										
	All (N=81)									
	Grade 1		Grade 2		Grade 3		Grade 4		Grade 5	
	Number of events	Number of affected participants (%)	Number of events	Number of affected participants (%)	Number of events	Number of affected participants (%)	Number of events	Number of affected participants (%)	Number of events	Number of affected participants (%)
All events	5	5 (6.2)	3	3 (3.7)	0	0 (0.0)	0	0 (0.0)	0	0 (0.0)
Infections and infestations	3	3 (3.7)	1	1 (1.2)	0	0 (0.0)	0	0 (0.0)	0	0 (0.0)
Cystitis	1	1 (1.2)	0	0 (0.0)	0	0 (0.0)	0	0 (0.0)	0	0 (0.0)
Sinusitis	0	0 (0.0)	1	1 (1.2)	0	0 (0.0)	0	0 (0.0)	0	0 (0.0)
Upper respiratory tract infection	1	1 (1.2)	0	0 (0.0)	0	0 (0.0)	0	0 (0.0)	0	0 (0.0)
Pharyngotonsillitis	1	1 (1.2)	0	0 (0.0)	0	0 (0.0)	0	0 (0.0)	0	0 (0.0)
Skin and subcutaneous tissue disorders	0	0 (0.0)	2	2 (2.5)	0	0 (0.0)	0	0 (0.0)	0	0 (0.0)
Eczema	0	0 (0.0)	1	1 (1.2)	0	0 (0.0)	0	0 (0.0)	0	0 (0.0)
Urticaria	0	0 (0.0)	1	1 (1.2)	0	0 (0.0)	0	0 (0.0)	0	0 (0.0)
Laboratory test	2	2 (2.5)	0	0 (0.0)	0	0 (0.0)	0	0 (0.0)	0	0 (0.0)
Abnormal liver function	2	2 (2.5)	0	0 (0.0)	0	0 (0.0)	0	0 (0.0)	0	0 (0.0)

Side effects

	EFE group (N=41)									
	Grade 1		Grade 2		Grade 3		Grade 4		Grade 5	
	Number of events	Number of affected participants (%)	Number of events	Number of affected participants (%)	Number of events	Number of affected participants (%)	Number of events	Number of affected participants (%)	Number of events	Number of affected participants (%)
All events	1	1 (2.4)	1	1 (2.4)	0	0 (0.0)	0	0 (0.0)	0	0 (0.0)
Skin and subcutaneous tissue disorders	0	0 (0.0)	1	1 (2.4)	0	0 (0.0)	0	0 (0.0)	0	0 (0.0)
Eczema	0	0 (0.0)	1	1 (2.4)	0	0 (0.0)	0	0 (0.0)	0	0 (0.0)
Laboratory test	1	1 (2.4)	0	0 (0.0)	0	0 (0.0)	0	0 (0.0)	0	0 (0.0)
Abnormal liver function	1	1 (2.4)	0	0 (0.0)	0	0 (0.0)	0	0 (0.0)	0	0 (0.0)

Side effects

	Placebo group (N=40)									
	Grade 1		Grade 2		Grade 3		Grade 4		Grade 5	
	Number of events	Number of affected participants (%)	Number of events	Number of affected participants (%)	Number of events	Number of affected participants (%)	Number of events	Number of affected participants (%)	Number of events	Number of affected participants (%)
All events	1	1 (2.5)	0	0 (0.0)	0	0 (0.0)	0	0 (0.0)	0	0 (0.0)
Skin and subcutaneous tissue disorders	0	0 (0.0)	0	0 (0.0)	0	0 (0.0)	0	0 (0.0)	0	0 (0.0)
Eczema	0	0 (0.0)	0	0 (0.0)	0	0 (0.0)	0	0 (0.0)	0	0 (0.0)
Laboratory test	1	1 (2.5)	0	0 (0.0)	0	0 (0.0)	0	0 (0.0)	0	0 (0.0)
Abnormal liver function	1	1 (2.5)	0	0 (0.0)	0	0 (0.0)	0	0 (0.0)	0	0 (0.0)

Side effects

	All (N=81)									
	Grade 1		Grade 2		Grade 3		Grade 4		Grade 5	
	Number of events	Number of affected participants (%)	Number of events	Number of affected participants (%)	Number of events	Number of affected participants (%)	Number of events	Number of affected participants (%)	Number of events	Number of affected participants (%)
All events	2	2 (2.5)	1	1 (1.2)	0	0 (0.0)	0	0 (0.0)	0	0 (0.0)
Skin and subcutaneous tissue disorders	0	0 (0.0)	1	1 (1.2)	0	0 (0.0)	0	0 (0.0)	0	0 (0.0)
Eczema	0	0 (0.0)	1	1 (1.2)	0	0 (0.0)	0	0 (0.0)	0	0 (0.0)
Laboratory test	2	2 (2.5)	0	0 (0.0)	0	0 (0.0)	0	0 (0.0)	0	0 (0.0)
Abnormal liver function	2	2 (2.5)	0	0 (0.0)	0	0 (0.0)	0	0 (0.0)	0	0 (0.0)

Table S12 Part 2: changes in physical findings

Systolic pressure [mmHg]

	EFE group (N=41)		Placebo group (N=40)	
	Measurement	Change	Measurement	Change
Pre-screening test/Day1				
Number of participants	41		40	
Mean (standard deviation)	118.2 (17.9)		122.3 (18.0)	
Median	116.0		118.0	
[Minimum, Maximum]	[84, 173]		[98, 165]	
Day3				
Number of participants	41	41	40	40
Mean (standard deviation)	115.3 (15.2)	-2.9 (10.9)	117.9 (17.4)	-4.3 (11.9)
Median	110.0	-5.0	115.0	-6.0
[Minimum, Maximum]	[98, 171]	[-20, 26]	[89, 155]	[-28, 23]
Day7				
Number of participants	41	41	39	39
Mean (standard deviation)	112.1 (13.5)	-6.1 (13.3)	116.8 (17.8)	-4.5 (11.3)
Median	110.0	-6.0	116.0	-6.0
[Minimum, Maximum]	[84, 157]	[-52, 23]	[83, 148]	[-30, 26]
Day15/At termination				
Number of participants	41	41	40	40
Mean (standard deviation)	117.1 (16.0)	-1.1 (15.7)	122.1 (18.0)	-0.2 (17.7)
Median	116.0	-1.0	118.0	-1.0
[Minimum, Maximum]	[89, 155]	[-33, 37]	[93, 178]	[-40, 42]

Diastolic pressure[mmHg]

	EFE group (N=41)		Placebo group (N=40)	
	Measurement	Change	Measurement	Change
Pre-screening test/Day1				
Number of participants	41		40	
Mean (standard deviation)	79.2 (12.2)		80.0 (14.8)	
Median	78.0		79.0	
[Minimum, Maximum]	[58, 107]		[57, 115]	
Day3				
Number of participants	41	41	40	40
Mean (standard deviation)	75.5 (11.4)	-3.7 (10.6)	77.2 (15.1)	-2.8 (9.5)
Median	74.0	-4.0	76.5	-1.5
[Minimum, Maximum]	[58, 107]	[-28, 24]	[43, 104]	[-24, 15]
Day7				
Number of participants	41	41	39	39
Mean (standard deviation)	69.6 (10.8)	-9.6 (7.5)	75.6 (13.8)	-3.7 (8.8)
Median	68.0	-12.0	74.0	-3.0
[Minimum, Maximum]	[52, 100]	[-26, 4]	[51, 101]	[-25, 17]
Day15/At termination				
Number of participants	41	41	40	40
Mean (standard deviation)	73.0 (12.5)	-6.2 (13.9)	74.4 (11.4)	-5.6 (13.2)
Median	72.0	-6.0	75.0	-4.5
[Minimum, Maximum]	[51, 98]	[-41, 32]	[54, 99]	[-39, 22]

Body temperature [℃]

	EFE group (N=41)		Placebo group (N=40)	
	Measurement	Change	Measurement	Change
Pre-screening test/Day1				
Number of participants	41		40	
Mean (standard deviation)	36.66 (0.35)		36.78 (0.77)	
Median	36.70		36.65	
[Minimum, Maximum]	[35.6, 37.6]		[35.6, 39.9]	
Day3				
Number of participants	41	41	40	40
Mean (standard deviation)	36.44 (0.35)	-0.22 (0.40)	36.40 (0.39)	-0.38 (0.69)
Median	36.50	-0.20	36.40	-0.30
[Minimum, Maximum]	[35.3, 37.0]	[-1.5, 0.5]	[35.7, 37.4]	[-3.4, 0.9]
Day7				
Number of participants	41	41	39	39
Mean (standard deviation)	36.53 (0.41)	-0.13 (0.48)	36.41 (0.41)	-0.39 (0.81)
Median	36.60	0.00	36.40	-0.20
[Minimum, Maximum]	[35.1, 37.3]	[-1.4, 0.6]	[35.2, 37.2]	[-3.2, 0.9]
Day15/At termination				
Number of participants	41	41	40	40
Mean (standard deviation)	36.47 (0.39)	-0.19 (0.58)	36.50 (0.30)	-0.28 (0.77)
Median	36.50	-0.20	36.50	-0.20
[Minimum, Maximum]	[35.0, 37.1]	[-1.9, 0.9]	[35.6, 37.1]	[-3.3, 1.1]

Pulse [bpm]

	EFE group (N=41)		Placebo group (N=40)	
	Measurement	Change	Measurement	Change
Pre-screening test/Day1				
Number of participants	41		40	
Mean (standard deviation)	81.8 (11.7)		83.8 (12.7)	
Median	80.0		85.0	
[Minimum, Maximum]	[55, 105]		[56, 114]	
Day3				
Number of participants	41	41	40	40
Mean (standard deviation)	78.8 (12.2)	-3.0 (10.4)	76.4 (10.2)	-7.4 (10.8)
Median	79.0	-4.0	77.3	-5.3
[Minimum, Maximum]	[60, 122]	[-19, 21]	[53, 92]	[-41, 14]
Day7				
Number of participants	41	41	39	39
Mean (standard deviation)	78.4 (11.4)	-3.4 (11.0)	78.8 (10.4)	-4.8 (11.6)
Median	77.0	-1.5	78.5	-3.0
[Minimum, Maximum]	[56, 101]	[-29, 20]	[57, 100]	[-36, 19]
Day15/At termination				
Number of participants	41	41	40	40
Mean (standard deviation)	79.8 (12.3)	-1.9 (11.4)	79.3 (10.4)	-4.4 (11.9)
Median	78.5	-2.0	78.0	-3.8
[Minimum, Maximum]	[47, 110]	[-21, 21]	[57, 98]	[-28, 24]

SpO$_2$[%]

	EFE group (N=41)		Placebo group (N=40)	
	Measurement	Change	Measurement	Change
Pre-screening test/Day1				
Number of participants	41		40	
Mean (standard deviation)	98.23 (0.63)		98.20 (0.67)	
Median	98.50		98.00	
[Minimum, Maximum]	[96.5, 99.5]		[97.0, 99.5]	
Day3				
Number of participants	41	41	40	40
Mean (standard deviation)	97.80 (2.07)	-0.43 (2.18)	98.13 (0.50)	-0.08 (0.72)
Median	98.00	0.00	98.00	0.00
[Minimum, Maximum]	[85.5, 99.5]	[-13.0, 1.5]	[97.0, 99.0]	[-1.5, 1.5]
Day7				
Number of participants	41	41	39	39
Mean (standard deviation)	98.16 (0.82)	-0.07 (1.05)	98.35 (0.63)	0.14 (0.79)
Median	98.00	0.00	98.50	0.00
[Minimum, Maximum]	[97.0, 100.0]	[-2.0, 2.0]	[97.0, 100.0]	[-1.5, 2.0]
Day15/At termination				
Number of participants	41	41	40	40
Mean (standard deviation)	98.27 (0.90)	0.04 (0.90)	98.41 (0.72)	0.21 (0.86)
Median	98.00	0.00	98.50	0.00
[Minimum, Maximum]	[96.0, 100.0]	[-2.0, 2.0]	[97.0, 100.0]	[-1.5, 2.0]
